# Safety after extended repeated use of ulipristal acetate for uterine fibroids

**DOI:** 10.1371/journal.pone.0173523

**Published:** 2017-03-07

**Authors:** Bart C. J. M. Fauser, Jacques Donnez, Philippe Bouchard, David H. Barlow, Francisco Vázquez, Pablo Arriagada, Sven O. Skouby, Santiago Palacios, Janusz Tomaszewski, Boguslaw Lemieszczuk, Alistair R. W. William

**Affiliations:** 1 Department of Reproductive Medicine & Gynecology, University Medical Center Utrecht, Utrecht, The Netherlands; 2 Société de Recherche pour l'Infertilité, Brussels, Belgium; 3 Endocrinology Unit, AP-HP Hospital Saint-Antoine, Paris, France; 4 College of Medical, Veterinary, and Life Sciences, University of Glasgow, Glasgow, Scotland, UK and Hamad Medical Corporation, Doha, Qatar; 5 Centro de Estudios de Obstetricia y Ginecología Asociado, Lugo, Spain; 6 Gedeon Richter/PregLem S.A., Geneva, Switzerland; 7 Division of Reproductive Endocrinology, Dept OB/GYN, Herlev-Gentofte Hospital, Faculty of Health and Medical Sciences, University of Copenhagen, Copenhagen, Denmark; 8 Palacios Institute of women’s Health, Madrid, Spain; 9 Prywatna Klinika Polozniczo-Ginekologiczna, Bialystok, Poland; 10 Gabinet Lekarski Specjalistyczny Sonus, Warsaw, Poland; 11 University of Edinburgh, Department of Pathology Royal Infirmary of Edinburgh, Edinburgh, United Kingdom; Duke University, UNITED STATES

## Abstract

**Objective:**

To assess long term safety of extended repeated 3-month courses of ulipristal acetate (UPA) 10 mg/day, for up to 8 courses, with focus on endometrial and laboratory safety parameters.

**Methods:**

This long-term, multi-center, open-label cohort, follow up study consisted of up to 8 consecutive 3-month courses of daily UPA 10 mg, each separated by a drug free period of 2 spontaneous menstrual bleeds. Sixty-four pre-menopausal women, with moderate to severe symptomatic uterine myoma(s) and heavy bleeding were enrolled and were studied for approximately 4 years. The main outcome measures were endometrial histology, laboratory parameters and general safety.

**Results:**

All data was reported in a descriptive manner with no formal statistical comparisons. In the 64 women, non-physiological changes (mostly cyst formation, epithelial and vascular changes) in endometrial histology at screening and after treatment courses 4 and 8 were observed in 18.0%, 21.4% and 16.3% of biopsies, respectively. After treatment cessation, such changes were observed in 9.1% of biopsies. All endometrial biopsies were benign after course 8. The median endometrial thickness was 7.0 mm, 10–18 days after the start of menses following treatment courses 5–8, compared to 9.0 mm at screening (before UPA treatment). No changes in the number and type of laboratory results outside the normal ranges were observed with the increasing treatment courses. In total, adverse events were reported in 10 (16%), 12 (19%), 8 (14%) and 5 (9%) subjects, during treatment courses 5, 6, 7 and 8, respectively of which the most frequent adverse events were headache and hot flush.

**Conclusion:**

The results of this study further support the safety profile of extended repeated 3 months treatment of symptomatic fibroids with ulipristal acetate 10 mg/day. Repeated UPA treatment courses did not result in any changes of concern in endometrial histology, endometrial thickness, or laboratory safety measures.

## Introduction

Selective progesterone receptor modulators (SPRMs) offer novel and unique medical treatment options in gynecology [1] and the potential of SPRM for the treatment of uterine fibroids has been well established [2]. Ulipristal acetate (UPA) represents a SPRM which has been demonstrated to effectively control excessive bleeding due to uterine fibroids and may lead to fibroid shrinkage [3] [4]. After treatment cessation, this reduction in fibroid volume may be sustained for up to 6 months [3] [4] with menstruation usually returning within 4 weeks. In addition, treatment with UPA corrects hemoglobin and hematocrit levels, reduces fibroid-associated pain and improves quality of life [3] [4].

SPRMs demonstrate predominant anti-progesterone activity and their administration can lead to a pattern of benign, non-physiological, endometrial histological features referred to as Progesterone receptor modulator Associated Endometrial Changes (PAEC). These changes are characterized by cystic glandular dilatation, apoptosis, low mitotic activity in the glands and stroma, absence of stromal breakdown, and glandular crowding [5] [6]. The histological diagnosis of PAEC is made by the observation of simultaneous presence of a variable range of non-physiological endometrial changes, none of which is sufficient for the diagnosis on its own. Previous clinical studies illustrated the reversibility of PAEC induced by UPA on endometrial biopsies [3] [4]. Moreover, the occurrence of non-physiological changes of the endometrium does not increase with up to 4 treatment courses and returns to pretreatment levels within 3 months of completion of treatment, as recently reported [7].

UPA (5 mg/day, for 3 months) is currently licensed for pre-operative and intermittent treatment of moderate to severe symptoms of uterine fibroids in adult women of reproductive age. The effect of repeated intermittent use of UPA 5 or 10 mg daily (up to 4 courses) have been previously studied [7]; However long term safety of up to 8 courses of treatment separated by off treatment periods of 2 menstrual cycles, for up to 4 years of treatment and subject follow up in total, considering the long term safety implications, is believed to be of special interest for clinicians. Under such circumstances, UPA may be employed in women of more advanced reproductive age approaching menopause avoiding the need for any surgery.

## Materials and methods

### Study design

PEARL extension 2 was an optional, long-term, open-label extension, phase III study, available to all subjects who had previously completed 4 treatment courses with UPA 10 mg as part of the PEARL III studies, previously published [8]. The study consisted of 4 additional 3-month courses of UPA 10 mg once daily with drug free periods of 2 spontaneous menstrual bleedings, bringing the total number of UPA treatment courses since inclusion to 8. The treatment satisfaction of subjects receiving repeated intermittent ulipristal acetate treatment courses was also assessed but this report focuses on the safety aspects of the study.

This study was conducted at 16 investigation centers in four countries in Europe from July 2010 (screening) until March 2015 (last subject out). The study was approved by the independent ethics committee of each participating site and were conducted in accordance with the International Conference on Harmonisation-Good Clinical Practice guidelines. Names and addresses of Central Ethics Committees by country:

**Austria**
Ethik-Kommission der Medizinischen Universität Wien und des Allgemeinen
Krankehauses der Stadt Wien
Borschkegasse 8b/E 06
(Dienstzimmergebäude, BT 68)
A–1090 Wien

**Belgium**
Comité d'Ethique Hospitalo-Facultaire Saint-Luc-UCL
AVENUE HIPPOCRATE, 55/14 Tour Harvey—Niveau 0
1200 BRUXELLES-20

**Poland**
Komisja Bioetyczna
przy Okręgowej Izbie Lekarskiej
ul. Świętojańska 7
15–082 Białystok

**Spain**
CEIC Área 2—Hospital Universitario de La Princesa
Cecilia López / D.Francisco Abad
C/ Diego de León, 62
28006 Madrid

The study was designed by the sponsor (Gedeon Richter/PregLem S.A.). Clinical trial registration: ClinicalTrials.gov; NCT01642472.

### Study population

Subjects enrolled in the study were women of reproductive age, with moderate to severe symptomatic uterine myoma(s) with at least one fibroid ≥ 3cm in diameter and none>10cm, heavy menstrual bleeding, and uterine size <16 weeks of gestation. Eligible women were aged 18–48 years, with body mass index 18–40 (kg/m2) and regular menstrual cycles of 22–35 days with FSH<20 IU/L. Written informed consent was obtained from all subjects.

### Intervention

After finalizing treatment course 4, women could either leave the study and attend a final follow-up visit 3 months later (PEARL III extension) or, enroll in this optional PEARL extension 2 study to obtain up to four further courses of UPA 10 mg, each separated by an off-treatment period including a full menstrual cycle up to the start of the second menstruation. Screening (baseline values) for each subject were those obtained before initiating treatment previous to the first treatment course. Treatment course 5 was started on the first day of the first menstruation following the first visit in the study i.e. at least 3 months after the end of treatment course 4. The subsequent treatment courses 6, 7 and 8 were started on the first day of the second menses after end of the previous treatment course. The follow-up visit was conducted approximately 3 months after the final treatment course.

### Assessments

#### Endometrial histology

Endometrial biopsy samples were obtained 10–18 days after menstruation start at screening and post-treatment courses 4 and 8, and at 3 months post-treatment course 8 if required (biopsies were to be performed only if 1 or more pathologists had provided a diagnosis other than “benign physiologic endometrium” for the biopsy after treatment course 8 or if the biopsy was judged to be not adequate for histology review). The sample collection of endometrial tissue was performed by a gynecologist using a Pipelle de Cornier. All samples were assessed in a blinded manner by three independent pathologists experienced in PAEC. A consensus diagnosis was considered if 2 out of the 3 pathologists were in agreement of the histological assessment for each sample.

#### Laboratory parameters

Clinical laboratory testing (hemoglobin, creatinine, total bilirubin, alanine transaminase [ALT], aspartate transaminase [AST], total cholesterol, high density lipoprotein [HDL], low density lipoprotein [LDL] and triglycerides) was analyzed in a central clinical laboratory. Blood samples for laboratory safety examinations were collected at screening, after treatment course 4, at 3 months post-treatment course 4 and after treatment course 8. Subjects were asked to fast overnight before blood sampling for lipids were assessed. Laboratory results were reported and summarized using standard international units and were assessed for clinical significance by the investigator or one of the co-investigators.

#### Endometrial thickness

Endometrial thickness was assessed by transvaginal ultrasound 10–18 days after start of menses following treatment courses 6 and 8 and at the 3-month follow-up visit. When possible, the US was performed by the same assessor at each visit.

### Statistical analysis

This study was a single-arm, open-label cohort, follow-up study and it was therefore impossible to estimate how many subjects would elect to participate in the additional 4 course (i.e. 2 years of treatment) and as such no formal statistical sample size or hypothesis testing were planned. The main aim of the study was to accumulate extended long term safety information and patient satisfaction with treatment, not to compare any of the results to either other treatments or to historical data. As a result, no formal statistical testing and therefore p-values were calculated for any of the parameters in the study and only descriptive statistics were created, to allow any potential safety signals to be identified, and to ensure the patient satisfaction with this type of long term intermittent treatment. Of the 99 subjects who had previously completed 4 treatment courses, 64 subjects opted to continue in to this study. Safety analysis was conducted on the Full Analysis Set (FAS). The population of primary interest was the FAS, which was defined as all enrolled subjects who receive study medication at least once during the study.

The statistical analysis was performed using Version 9.3 of the SAS System for Windows ([2002–2010] SAS Institute Inc).

## Results

The demographic characteristics of all subjects (FAS) are provided in Table 1; demographic data for subjects in this study were collected at baseline/screening before the first UPA treatment course 1.

**Table 1:** Baseline demographic characteristics.

Characteristics		Full Analysis Set (N = 64)
Age (*)	Mean ± SD	41.5 ± 4.8
Ethnic Origin	White	60 (93.8%)
	Black	3 (4.7%)
	Asian	0
	Hispanic	1 (1.6%)
	Other	0
Weight (kg)	Mean ± SD	67 ± 13.16
Body Mass Index (kg/m^2^)	Mean ± SD	24.89 ± 4.71

(*) Age at last birthday in whole years, using the date of informed consent (PEARL III study, started in July 2010).

Note: Only month and year of birth were recorded. The 1st of the month is used for the day of birth.

Demographic data was collected at Screening / Baseline of the PEARL III study (before the first UPA treatment course 1).

SD: Standard Deviations

A total of 64 (100%), 62 (97%), 56 (88%) and 54 (84%) subjects started UPA 10 mg treatment courses 5, 6, 7 and 8, respectively. A total of 11 (17%) subjects discontinued the study at any time. The reasons for discontinuation were: lack of efficacy (N = 2), subject request (N = 2), surgery or procedure for fibroids (N = 2), pregnancy (N = 2), adverse event (not UPA 10 mg-related) (N = 1), missing return of menstruation (N = 1) and ‘other reason’ (N = 1).

**Fig 1:**
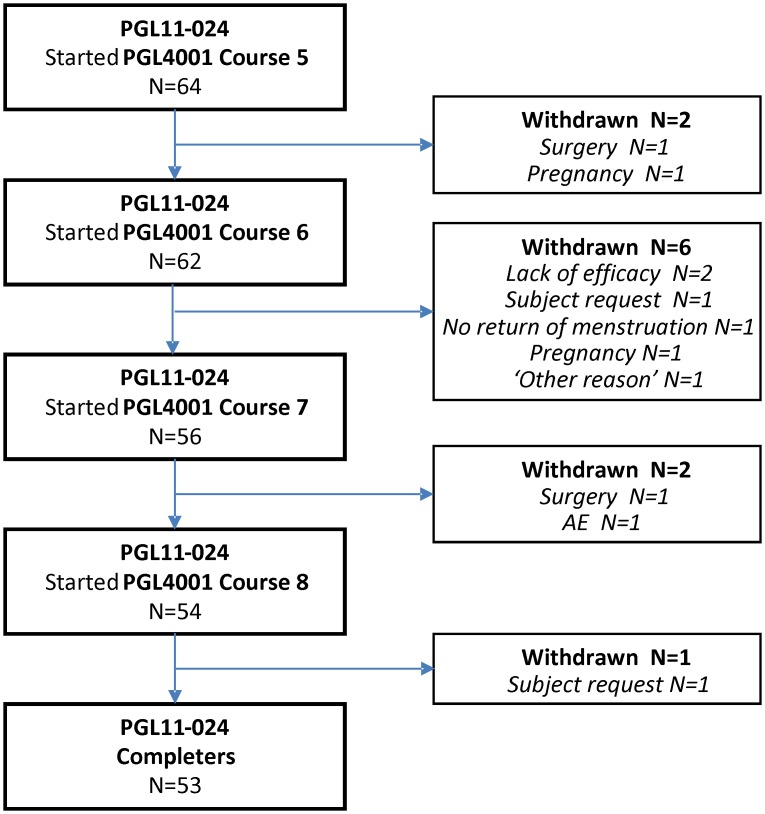
Flow chart of subject disposition during PEARL extension 2. Withdrawals are presented according to the timeframes in which they occurred, either during treatment or after treatment completion for each course.

The 3-month follow-up visit after last dose of course 8 was attended by 53 (83%) subjects. The majority of subjects who completed the study were study participants for approximately 4 years from July 2010 until March 2015. Subject disposition is shown in Fig 1. Fourteen (22%) of the 64 subjects who continued in to this extension study started the study on the same day as the 3-month follow-up visit post-treatment course 4. Fifty (78%) of the 64 subjects had completed treatment course 4 prior to starting this study (due to delayed study start-up), and for these subjects the mean (median) number of days between the 3-month follow-up visit post-treatment course 4 and the first visit in this study was 103.9 (91.0) days which translates to a 3-month longer variable off drug period.

### Safety results

#### Endometrial thickness

The median endometrial thickness remained below that seen at screening, with no evidence of an increase in the number of subjects with a thickness >16 mm as the number of treatment courses increased. At screening, for the 62 subjects with data available, the median endometrial thickness was 9.0 mm (range 3–21 mm), and 2 (3.2%) subjects had thickness >16 mm. After the start of menses following treatment course 8, for the 49 subjects with data available (four subjects did not perform this visit due to a delay in restart of menstruation following the end of treatment course 8), the median endometrium thickness was 7.0 mm (range 3–23 mm), and 1 (2.0%) subject had thickness >16 mm. At the 3-month follow-up visit post-treatment course 8 for the 53 subjects with data available, the median endometrial thickness remained at 7.0 mm (range 1–16 mm), and no subjects had thickness >16 mm.

#### Endometrial histology

Endometrial biopsy samples were evaluated by the same 3 independent pathologists during treatment courses 1–8. A consensus diagnosis of benign endometrium was made for all (100%) endometrial biopsy samples adequate for histology review, taken from screening (96.2% adequate biopsies) through to the 3-month follow-up visit post-treatment course 8 (91.7% adequate biopsies). Results are summarized in Table 2. All 3 individual pathologists provided a diagnosis of benign endometrium in all evaluations with the exception of one single individual observation of complex, non-atypical, hyperplasia reported by one pathologist only and seen in one biopsy taken following treatment course 4. No intervention was performed, and a subsequent biopsy revealed a normal endometrium confirmed by 3 pathologists before starting course 5, which demonstrates that spontaneous resolution of hyperplasia is in line with published literature [9] [10].

**Table 2:** Summary of endometrium biopsy consensus and endometrium biopsy non-physiological descriptions (PAEC) (Full analysis set, N = 64).

	Screening	After course 4	After Course 8	3-month after course 8
Total Biopsies	52	61	48	24
Adequate Biopsies (^1^*)	50 (96.2%)	56 (91.8%)	43 (89.6%)	22 (91.7%)
Benign (^2^**)	50 (100%)	56 (100%)	43 (100%)	22 (100%)
Hyperplasia (^2^**)	0	0	0	0
Malignant neoplasm (^2^**)	0	0	0	0
Non-physiological changes observed by two or three pathologists**	9 (18.0%)	12 (21.4%)	7 (16.3%)	2 (9.1%)

^1^ Endometrium biopsy performed and assessed as adequate by at least one pathologist.

^2^ Of those who deem specimen adequate, at least two assessors have the same opinion; otherwise the most severe is used.

* Denominator of percentage is the number of subjects that have endometrium biopsy performed.

** Denominator of percentage is the number of subjects with an adequate specimen.

#### PAEC

Summaries of non-physiological changes (made up of the following non-physiological descriptions: epithelial changes, extensive cyst formation, unusual vascular changes and “other” non-physiologic changes) observed by at least 2 of the 3 pathologists suggested that after screening, when non-physiological changes were observed in 18.0% of biopsies, the incidence in the full study population was highest in biopsies taken following treatment course 1, seen in 35.0% of biopsies, and then subsequently decreased. Following treatment course 4, at least 2 pathologists observed non-physiological changes in 21.4% of biopsies. After course 8, non-physiological changes were observed in 16.3% of biopsies, which is comparable to baseline frequency. The majority of the non-physiological diagnosis (71.4%) was achieved by 2 of the 3 pathologists. In all biopsies, the 3rd diagnosis (not in consensus) was of a benign endometrium.

No additional observations were reported for biopsy samples during this study except the presence of polyps, seen for 2 subjects prior to treatment course 5 and 2 subjects after treatment course 8. Before treatment course 5, one hyperplastic polyp was diagnosed for one subject and benign polyp for one subject; for both of these a biopsy was not done after treatment course 6, but the polyps were absent after treatment course 8. Benign polyp was diagnosed for 2 subjects after treatment course 8 but was absent at the 3-month follow-up visit.

#### General safety

Adverse events were collected according to standardized method predefined in the study protocol. Adverse events were reported in 10 (16%), 12 (19%), 8 (14%) and 5 (9%) subjects, during treatment courses 5, 6, 7 and 8, respectively. No serious adverse events (SAEs) were reported during this study. One subject was diagnosed as pregnant during treatment course 5 and was already published [11]. One subject withdrew approximately 2 months following the end of treatment course 7, as she chose to undergo an abdominal hysterectomy. Overall, during treatment courses 1–8, the majority (98.4%) of all on-treatment adverse events (AEs) were rated as being of mild or moderate intensity.

During treatment courses 1–8, the most commonly reported on-treatment AEs was headache, with the greatest incidence reported by 7 (10.9%) subjects during the first treatment course. A total of 20 (31.3%) subjects experienced at least one on-treatment AEs considered to be UPA 10 mg-related. Overall, there was no evidence that any specific AEs increased in frequency or intensity as the number of UPA 10 mg treatment courses increased, nor were any unexpected AEs reported during this study.

#### Laboratory parameters

Evaluation of laboratory parameters, including changes from screening was undertaken as part of the review of safety. No changes in the number and type of laboratory results falling outside of the normal ranges were observed as the number of treatment courses increased. A summary of laboratory parameters is found in Table 3

**Table 3:** Summary of laboratory parameters (Full analysis set, N = 64).

	Screening		After course 4		3 months post treatment course 4		After course 8	
Parameter (unit), normal range	N	Mean ± SD	N	Mean ± SD	N	Mean ± SD	N	Mean ± SD
Hemoglobin (g/dL), 11.5–15.5	63	12.8 ± 1.57	64	13.0 ± 1.36	63	12.8 ± 1.34	48	13.3 ± 0.95
Creatinine (umol/L), 45–84	63	61.4 ± 8.5	64	60.8 ± 8.6	63	63.0 ± 10.0	48	64.3 ± 9.8
Total bilirubin (umol/L), 0–19	63	6.5 ± 3.3	64	7.0 ± 3.6	63	7.1 ± 3.9	47	7.3 ± 3.0
AST (U/L), 0–37	63	21.3 ± 5.0	64	20.5 ± 4.5	63	21.1 ± 6.6	48	19.5 ± 4.8
ALT (U/L), 0–47	63	18.6 ± 6.7	64	16.0 ± 6.1	63	17.2 ± 10.6	48	16.8 ± 6.6
Total Cholesterol (mmol/L), 0–5.17	63	5.3 ± 0.79	60*	5.5 ± 0.82*	63	5.3 ± 0.72	49	5.3 ± 0.94
HDL (mmol/L), 1.04–25.88	63	1.7 ± 0.36	60*	1.7 ± 0.41*	63	1.7 ± 0.38	49	1.7 ± 0.35
LDL (mmol/L), 0–2.58	62	3.1 ± 0.73	60*	3.3 ± 0.79*	63	3.1 ± 0.70	49	3.1 ± 0.84
Triglycerides (mmol/L), 0–1.69	63	1.3 ± 0.88	60*	1.2 ± 0.73*	63	1.1 ± 0.70	49	1.3 ±0.82

SD: Standard Deviations

Measurements after treatment courses 4 and 8:10–18 days after the first menstruation following treatment courses 4 and 8

* Measurements in the last 2 weeks of treatment course 4

There were no changes in vital signs, including blood pressure and one subject experienced an unspecified increase of weight after treatment course 3.

## Discussion

The objective of the study was primarily to assess the safety of intermittent treatment courses of UPA 10 mg once daily, in women of reproductive age with symptomatic uterine fibroids bringing the total number of treatment courses to 8. This study provides, for the first time, evidence with regards to long term safety of UPA for the management of fibroids. Study participation was for a mean of 46 months (range 40 to 54 months) which corresponds to approximately 4 years of treatment and follow up. The general compliance and acceptability of treatment was high, as 83% of the subjects completed the study. In this study, only 4% of black subjects were enrolled, however two additional phase III studies are currently being conducted in the USA including a majority of subjects of African American origin which will allow to further assess the safety of this treatment in this ethnic group.

In previous studies, outcomes of up to 4 repeated intermittent treatment courses have been described [7] [8]. To date, this is the longest reported experience using a selective progesterone receptor modulator (SPRM) in the medical management of uterine fibroids.

Progesterone receptor modulator Associated Endometrial Changes (PAEC) is a phenomenon closely related to the use of SPRMs. It is a histological diagnosis dependent on the simultaneous appearance in SPRM-treated endometrium of several non-physiological changes, none of which is specific on its own. It has been previously demonstrated that these changes are rapidly reversible and the frequency of PAEC returns to baseline levels after 3 spontaneous menstrual bleeds following completion of 4 courses of 3 months each [7]. However, the potential endometrial effects of UPA after extended repeated use was unknown.

Previous studies suggested that following treatment with UPA, an increase in endometrial thickness from screening occurred even after one treatment course [3], but this increase subsequently decreased after return of menstruation following up to 4 repeated treatment courses [7]. The extension of treatment for up to 8 courses in the present study, demonstrated that after return of menstruation, the endometrial thickness was lower than seen at screening for the majority of subjects, with no evidence of increasing incidence of thickness > 16 mm.

At screening, before starting the first treatment course, non-physiological changes in endometrium were observed in 18 (10.9%) subjects from the initial population enrolled in PEARL III. Interestingly, when looking at the 64 subjects who continued to treatment courses 5 to 8, nine (18%) subjects had non-physiological changes reported in their original assessments at screening, so it is very likely that the higher frequency observed for the sub-group of subjects continuing on treatment, is due to a random effect or alternatively, this could also have been explained by a background level of non-physiological changes in their endometrium at baseline, that is unaffected by UPA, however none of these hypothesis can be confirmed in this study. It should be noted that non-physiological changes diminish in extent and severity after withdrawal of UPA treatment, and the histological diagnosis of PAEC can be difficult to make with certainty in this situation.

At the 3-month follow-up visit post-treatment course 8, biopsies were to be performed only if 1 or more pathologists had provided a diagnosis other than “benign physiologic endometrium” for the biopsy after treatment course 8 or if the biopsy was judged to be not adequate for histology review. A follow-up additional biopsy was provided by 24 subjects at the 3-month follow-up visit, and 22 biopsies were considered adequate for review. In this subgroup, non-physiological changes were observed by at least 2 pathologists in 9.1% of biopsies as shown in Table 2. However, taking into consideration that for more than half of all subjects no follow-up biopsy had been requested due to a diagnosis of “benign endometrium” from all 3 pathologists after treatment course 8 (separated by one menstrual bleed after treatment end), the observation of non-physiological changes in the full study population decreased to <5%, returning to a level which is no different from baseline and actually numerically lower in this study. This confirms the previously described rapid reversibility of non-physiological changes once treatment is stopped and menstruation returns. All biopsy samples had a diagnosis of benign endometrium by consensus review, with no cases of endometrial hyperplasia diagnosed in the study, which gives further re-assurance on the safety of repeated administration of UPA on the endometrial histology. Although Pipelle biopsy is not a reliable method to diagnose endometrial polyps, only 2 small benign polyps were identified prior to treatment course 5, and 2 after course 8. No polyps were identified on transvaginal ultrasound, and endometrial thickness was not increased. We acknowledge the fact that in some cases, consensus was not reached by all 3 pathologists and this should be subject of further investigation.

The frequency of on-treatment AEs observed was 31.3%; however, the most common AEs were headache and hot flush. Considering the long duration of the study, this seems to be acceptable. No SAEs were reported during the study.

The dose of 10 mg daily of UPA utilized in the current study was higher than the approved dose of 5 mg/day, however the safety profile of UPA for both doses has already been evaluated in previous studies [6].

Safety assessments including vital sign measurements, laboratory investigations, as well as AEs demonstrated that the extended repeated administration schedule, with a drug-free interval, was well tolerated and did not identify any new safety concerns.

We acknowledge the fact that this study has some limitations, such as a limited number of subjects, lack of a comparator arm, and that this optional extension study open to subjects who had previously completed 4 treatment courses could have biased the retention of those subjects with a positive response to treatment; In addition, as a result no formal statistical testing and therefore p-values were calculated for any of the parameters in the study and only descriptive statistics were created, to allow any potential safety signals to be identified, and to ensure the patient satisfaction with this type of long term intermittent treatment. However, none of these limitations were considered significant to impact the safety findings of this study which focusses on the endometrial findings.

In conclusion, the current study convincingly demonstrates that the extended intermittent administration of UPA 10 mg once daily for 3 months with drug-free intervals, bringing the total number treatment courses undertaken to 8, is well tolerated in women of reproductive age with symptomatic uterine myoma.

Repetition of treatment courses did not lead to any clinically relevant changes in endometrial histology, nor to increases in endometrial thickness. The frequency of non-physiological changes observed did not increase with repeated treatment courses and, as previously demonstrated, non-physiological changes were rapidly reversible.

## Supporting information

S1 Text Study protocol. (PDF)

S2 Text Clinical study report synopsis. (PDF)

S1 TREND Checklist TREND statement checklist. (DOC)

## References

[1] Bouchard P, Chabbert-Buffet N, Fauser BCJM. Selective progesterone receptor modulators in reproductive medicine: pharmacology, clinical efficacy and safety. Fertility and sterility. 2011; 96(5): 1175–89. 10.1016/j.fertnstert.2011.08.021 21944187

[2] Bestel E, Donnez J. The potential of selective progesterone receptor modulators for the treatment of uterine fibroids. Expert review of endocrinology & metabolism. 2014; 9:79–92 10.1586/17446651.2014.862495 30743741

[3] Donnez J, Tatarchuk TF, Bouchard P, Puscasiu L, Zakharenko NF, Ivanova T et al Ulipristal acetate versus placebo for fibroid treatment before surgery. The New England journal of medicine. 2012; 366:409–20. 10.1056/NEJMoa1103182 22296075

[4] Donnez J, Tomaszewski J, Vazquez F, Bouchard P, Lemieszczuk B, Baro F et al Ulipristal acetate versus leuprolide acetate for uterine fibroids. The New England journal of medicine. 2012; 366:421–32. 10.1056/NEJMoa1103180 22296076

[5] Mutter GL, Bergeron C, Deligdisch L, Ferenczy A, Glant M, Merino M et al The spectrum of endometrial pathology induced by progesterone receptor modulators. Modern Pathology. 2008; 21(5):591–8. 10.1038/modpathol.2008.19 18246050

[6] Williams AR, Bergeron C, Barlow DH, Ferenczy A. Endometrial morphology after treatment of uterine fibroids with the selective progesterone receptor modulator, ulipristal acetate. International journal of gynecological pathology. 2012; 31(6):556–69. 10.1097/PGP.0b013e318251035b 23018219

[7] Donnez J, Donnez O, Matule D, Ahrendt HJ, Hudecek R, Zatik J, et al Long-term medical management of uterine fibroids with ulipristal acetate. Fertility and sterility. 2016; 105:165–173 10.1016/j.fertnstert.2015.09.032 26477496

[8] Donnez J, Vazquez F, Tomaszewski J, Nouri K, Bouchard P, Fauser BC, et al Long-term treatment of uterine fibroids with ulipristal acetate. Fertility and sterility. 2014; 101:1565–73. 10.1016/j.fertnstert.2014.02.008 24630081

[9] Kurman RJ, Kaminski PF, Norris HJ. The behavior of endometrial hyperplasia. A long-term study of untreated hyperplasia in 170 patients. Cancer. 1985; 56:403–12. 4005805 10.1002/1097-0142(19850715)56:2<403::aid-cncr2820560233>3.0.co;2-x

[10] Lacey JV Jr, Sherman ME, Rush BB, Ronnett BM, Ioffe OB, Duggan MA, et al Absolute risk of endometrial carcinoma during 20-year follow-up among women with endometrial hyperplasia. Journal of Clinical Oncology. 2010; 28:788–92. 10.1200/JCO.2009.24.1315 20065186 PMC2834395

[11] Luyckx M, Squifflet JL, Jadoul P, Votino R, Dolmans MM, Donnez J. First series of 18 pregnancies after ulipristal acetate treatment for uterine fibroids. Fertility and sterility. 2014; 102: 1404–1409. 10.1016/j.fertnstert.2014.07.1253 25241376

