# Supplementary material for: Safety after extended repeated use of ulipristal acetate for uterine fibroids
Source: PLoS One. 2017 Mar 7;12(3):e0173523. doi: 10.1371/journal.pone.0173523 (PMC5340384; doi:10.1371/journal.pone.0173523)
Supplement: S1 Text — (PDF) [file pone.0173523.s001.pdf]

# CLINICAL STUDY PROTOCOL

| | |
|---|---|
| **Protocol Number:** | **PGL11-024** |
| **EudraCT Number:** | **2012-001465-33** |
| **Investigational Medicinal Product:** | **PGL4001 (ulipristal acetate)** |
| **Study Title:** | **A Phase III, multicentre, extension study investigating the efficacy and safety of repeated intermittent 3-month courses of open-label administration of ulipristal acetate, in subjects with symptomatic uterine myomas and heavy uterine bleeding.** |
| **Short Study Title:** | **PGL4001 Efficacy Assessment in Reduction of symptoms due to uterine Leiomyomata** |
| **Study Name:** | **PEARL extension 2** |
| **Final Protocol Date:** | **PGL11-024 Final 1.0 - 21.05.2012** |
| **Coordinating Investigator:** | **Pr. Jacques Donnez, M.D., PhD<br>Université Catholique de Louvain<br>Cliniques Universitaires St Luc<br>Gynaecology department<br>10 Avenue Hippocrate<br>1200 Brussels, Belgium** |
| **Study Sponsor:** | **PregLem S.A.<br>3, Chemin du Pré-Fleuri<br>1228 Plan-Les-Ouates, Geneva / Switzerland<br>Phone +41 22 884 03 40<br>Fax + 41 22 884 03 49** |
| **Project Director & Medical Responsible:** | **Elke Bestel, MD** |
| **Clinical Trial Manager** | **Annie Dacquin** |
| **Study Monitor:** | **ICON Clinical Research Limited<br>South County Business Park<br>Leopardstown<br>Dublin 18, Ireland** |

# SPONSOR AND CONTRACT RESEARCH ORGANISATION(S) SIGNATORY APPROVAL PAGE

The below signatories have read this trial protocol and agree with its principles. They agree to carry out the clinical trial in compliance with this protocol, with ICH Good Clinical Practices (ICH GCP), Good Laboratory Practices (GLP) and the applicable regulatory requirements.

**Sponsor:**

PregLem S.A., 3, Chemin du Pré-Fleuri, 1228, Plan-les-Ouates, Geneva, Switzerland

21.5. 2012

Signature
Elke Bestel, MD
Senior Vice President Clinical Operations / R&D Global Project Director/ Medical Responsible

Date of signature

21 MAY 2012

Signature
Annie Dacquin
Clinical Trial Manager

Date of signature

**Contract Research Organisation(s):**
ICON Clinical Research, 20 rue Troyon, 92310 Sevres, France

22 MAY 2012

Signature
Riad Hadjkali
Project Manager

Date of signature

# INVESTIGATOR ENDORSEMENT PAGE

I, the undersigned, am responsible for the conduct of the study at this site and agree to the following:

- I understand and will conduct the study according to the protocol, any approved protocol amendments, ICH GCP and all applicable regulatory authority requirements and national laws.
- I will not deviate from the protocol without prior written permission from the Sponsor and prior review and written approval from the Institutional Review Board or Independent Ethics Committee, except where necessary to prevent any immediate danger to the subject.
- I have read and understand fully the Investigator Brochure (IB) for PGL4001, and I am familiar with the Investigational Medicinal Product(s) (IMP) and its/their use according to this protocol.
- I have sufficient time to properly conduct and complete the trial within the agreed trial period, and I have available an adequate number of qualified staff and adequate facilities for the foreseen duration of the trial to conduct the trial properly and safely.
- I will ensure that any staff at my site(s) who are involved in the study conduct are adequately trained regarding the IMPs, the protocol and their responsibilities. In the case of delegating any of my study responsibilities I will provide the Sponsor with a Delegation of Activities certificate.

Signature Date of signature

*[Insert Name, academic qualifications]*

*[Insert Position (title)]*

*[Insert Address of Institution]*

# TABLE OF CONTENTS


SYNOPSIS ..... 11

1. BACKGROUND INFORMATION ..... 16

1.1. INTRODUCTION TO PGL4001 ..... 16
1.2. UTERINE MYOMA ..... 16
1.3. CONVENTIONAL TREATMENT OF UTERINE MYOMA ..... 17
1.4. SUMMARY OF PRE-CLINICAL STUDIES ..... 18
*1.4.1. Summary of Pharmacology ..... 18*
*1.4.2. Pharmacokinetics ..... 19*
*1.4.3. Safety Pharmacology ..... 19*
*1.4.4. Toxicology ..... 20*
1.5. SUMMARY OF CLINICAL STUDIES ..... 20
1.6. RATIONALE FOR THE CURRENT STUDY ..... 21
1.7. SUMMARY OF OVERALL RISK AND BENEFITS ..... 22

2. OBJECTIVES ..... 22

2.1. PRIMARY OBJECTIVE: ..... 22
2.2. SECONDARY OBJECTIVE: ..... 22
2.3. EXPLORATORY OBJECTIVES: ..... 23

3. ENDPOINTS ..... 23

3.1. PRIMARY ENDPOINTS: ..... 23
3.2. SECONDARY ENDPOINTS: ..... 23
3.3. EXPLORATORY ENDPOINTS: ..... 24

4. STUDY DESIGN ..... 24

5. STUDY POPULATION ..... 25

5.1. SUBJECTS ..... 25
5.2. ENTRY CRITERIA ..... 26
*5.2.1. Inclusion Criteria ..... 26*
*5.2.2. Exclusion Criteria ..... 26*

6. STUDY PROCEDURES AND ASSESSMENTS ..... 27

6.1. GENERAL INSTRUCTIONS ..... 27
6.2. OUTLINE OF STUDY PROCEDURES AND ASSESSMENTS ..... 28
*6.2.1. Visit I ..... 28*
*6.2.2. Visit II ..... 29*
*6.2.3. Visit III – Post-treatment period ..... 30*
*6.2.4. Visit IV – Follow-up visit ..... 31*
*6.2.5. Early termination visit ..... 32*
6.3. EFFICACY OBSERVATIONS AND MEASUREMENTS ..... 33
*6.3.1. Quality of life ..... 33*

6.3.2. *Pain* ... 33
6.3.3. *Myoma and uterine size* ... 33
6.3.4. *Uterine bleeding pattern* ... 34
6.4. SAFETY OBSERVATIONS AND MEASUREMENTS ... 34
6.4.1. *Body weight* ... 34
6.4.2. *Vital Signs* ... 34
6.4.3. *Gynaecological and Breast examination* ... 34
6.4.4. *Transvaginal ultrasound of ovary, uterus, endometrium and myomas* ... 34
6.4.5. *Laboratory Evaluation* ... 35
6.5. OTHER OBSERVATIONS AND MEASUREMENTS ... 35
6.5.1. *Endometrial Biopsy* ... 35
6.6. CONCOMITANT MEDICATIONS AND THERAPIES ... 36
6.6.1. *Permitted Medicines* ... 36
6.6.2. *Prohibited Medicines* ... 36
6.6.3. *Non-Drug Therapies* ... 36
6.7. SUBJECT COMPLETION AND WITHDRAWAL ... 37
6.7.1. *Subject Completion* ... 37
6.7.2. *Subject Withdrawal from Study* ... 37
6.7.3. *Subject Replacement* ... 38
6.8. PLANNED EXTENSION STUDIES ... 38

**7. INVESTIGATIONAL MEDICINAL PRODUCT AND OTHER DRUGS USED IN THE STUDY ... 38**

7.1. DESCRIPTION OF INVESTIGATIONAL MEDICINAL PRODUCTS ... 38
7.2. DOSAGE AND ADMINISTRATION ... 39
7.3. PACKAGING AND LABELLING ... 39
7.4. PREPARATION, HANDLING AND STORAGE ... 40
7.5. INVESTIGATIONAL MEDICINAL PRODUCT ACCOUNTABILITY ... 40
7.5.1. *Other drugs to be used in the study* ... 41
7.6. ASSIGNMENT TO TREATMENT GROUPS ... 41
7.7. ASSESSMENT OF INVESTIGATIONAL MEDICINAL PRODUCT COMPLIANCE ... 41
7.8. METHOD OF BLINDING ... 41
7.9. EMERGENCY UNBLINDING ... 42
7.10. TREATMENT OF OVERDOSE AND MISUSE ... 42

**8. ADVERSE EVENTS AND SERIOUS ADVERSE EVENTS ... 42**

8.1. ADVERSE EVENTS ... 42
8.1.1. *Definitions* ... 42
8.1.2. *Abnormal laboratory findings and other objective measurements* ... 43
8.1.3. *Baseline Medical Conditions* ... 44
8.1.4. *Exacerbation of uterine myoma* ... 44
8.1.5. *Adverse Events of Special Interest* ... 44
8.2. PROCEDURES FOR ELICITING, RECORDING AND REPORTING ADVERSE EVENTS ... 45
8.2.1. *Eliciting Adverse Events* ... 45
8.2.2. *Recording of Adverse Events in the CRF* ... 45
8.2.3. *Reporting of Adverse Events* ... 46

8.3. SERIOUS ADVERSE EVENTS ..... 46
*8.3.1. Definitions ..... 46*
*8.3.2. SAE urgent reporting procedure ..... 47*
*8.3.3. Study specific reporting procedure ..... 48*
8.4. REPORTING TO THE INDEPENDENT ETHICS COMMITTEE ..... 49
8.5. REPORTING PERIOD ..... 49
8.6. PREGNANCY AND IN UTERO DRUG EXPOSURE ..... 50
*8.6.1. Initial reporting of pregnancies: ..... 50*
*8.6.2. Follow-up of pregnancies: ..... 50*

**9. DATA ANALYSIS AND STATISTICS ..... 51**

9.1. TEST OF HYPOTHESES ..... 51
9.2. SAMPLE SIZE ..... 51
9.3. RANDOMISATION ..... 51
9.4. POPULATION FOR ANALYSIS ..... 51
9.5. DATA ANALYSIS ..... 52
*9.5.1. Baseline Assessment ..... 52*
*9.5.2. Primary Efficacy Analysis ..... 52*
*9.5.3. Secondary Efficacy Analysis ..... 52*
*9.5.4. Safety Analysis ..... 52*
*9.5.5. Exploratory Analysis ..... 53*
*9.5.6. Missing Data ..... 53*
9.6. STUDY SPECIFIC DATA ANALYSIS ..... 53
*9.6.1. Interim Analysis ..... 53*
*9.6.2. Final Analysis ..... 53*

**10. STUDY ADMINISTRATION ..... 53**

10.1. REGULATORY AND ETHICAL CONSIDERATIONS ..... 53
*10.1.1. Informed Consent ..... 53*
*10.1.2. Regulatory Authority Approval ..... 54*
*10.1.3. Independent Ethics Committee Requirements ..... 54*
*10.1.4. End of the study ..... 54*
10.2. INVESTIGATOR RESPONSIBILITIES ..... 54
*10.2.1. Coordinating Investigator ..... 54*
10.3. DATA MANAGEMENT ..... 55
10.4. STUDY MONITORING ..... 55
10.5. STUDY SUPERVISORY COMMITTEE (SSC) ..... 55
10.6. SUBJECT CONFIDENTIALITY ..... 55
10.7. QUALITY ASSURANCE ..... 56
10.8. STUDY OR SITE DISCONTINUATION ..... 56
10.9. RETENTION OF ESSENTIAL STUDY DOCUMENTS ..... 56

**11. REFERENCES ..... 57**

**12. APPENDICES** ..... **59**

APPENDIX A SCHEDULE OF STUDY ASSESSMENTS ..... 60
APPENDIX B PICTORIAL BLEEDING ASSESSMENT CHART ..... 62
APPENDIX C GLOBAL STUDY TREATMENT SATISFACTION QUESTIONNAIRE ..... 63
APPENDIX D UFS QUALITY OF LIFE QUESTIONNAIRE ..... 64
APPENDIX E EUROQUOL EQ-5D QUALITY OF LIFE QUESTIONNAIRE ..... 67
APPENDIX F VAS PAIN SCALE ..... 70
APPENDIX G LABORATORY PARAMETERS ..... 71
APPENDIX H PACKAGE INSERT OF PIPELLE DE CORNIER® ..... 72
APPENDIX I LIST OF CYP 3A4 INDUCERS AND STRONG INHIBITORS ..... 74
APPENDIX J WORLD MEDICAL ASSOCIATION DECLARATION OF HELSINKI ..... 75

# LIST OF ABBREVIATIONS

| | |
|---|---|
| AE | Adverse Event |
| AESI | Adverse Event of Special Interest |
| ALT | Alanine Transaminase |
| AST | Aspartate Transaminase |
| AUC | Area Under the Curve |
| Bcl-2 | B-Cell CLL/Lymphoma 2 |
| BMD | Bone Mineral Density |
| bpm | Beats Per Minute |
| CRA | Clinical Research Associate |
| CTM | Clinical Trial Manager |
| CYP | Cytochrome P450 |
| CYP3A4 | Cytochrome P450 3A4 |
| DNA | Deoxyribonucleic Acid |
| E2 | Estradiol |
| eCRF | electronic Case Report Form |
| EQ-5D | Questionnaire for measuring Health outcome from the EuroQol group |
| EU | European Union |
| FAS | Full Analysis Set |
| FDA | Food and Drug Administration |
| FSH | Follicle-stimulating Hormone |
| GCP | Good Clinical Practice |
| GGT | Gamma Glutamyl Transferase |
| GLP | Good Laboratory Practice |
| GMP | Good Manufacturing Practice |
| GnRH | Gonadotropin-Releasing Hormone |
| HDL | High Density Lipoprotein |

| | |
|---|---|
| IB | Investigator Brochure |
| ID | Subject Identification Number |
| ICH | International Conference on Harmonization |
| IEC | Independent Ethics Committee |
| IMP | Investigational Medicinal Product |
| INN | International Non-proprietary Name |
| LDL | Low Density Lipoprotein |
| LH | Luteinising Hormone |
| mmHg | Millimeter of mercury |
| NIH | National Institutes of Health |
| NOAEL | No Observed Adverse Effect Level |
| PAEC | Progesterone receptor modulator Associated Endometrial Changes |
| PAP | Papanikolaou test |
| PARP | Poly ADP ribose polymerase |
| PBAC | Pictorial Bleeding Assessment Chart |
| P-gp | P-glycoprotein |
| PGL4001 | Ulipristal Acetate (17α-Acetoxy-11β-(4-N,N-dimethylaminophenyl)-19-norpregna-4,9-diene-3,20-dione) |
| PR(-B) | Progesterone Receptor (type B) |
| PRM | Progesterone Receptor Modulator |
| PSF | Pregnancy Surveillance Form |
| PVC/PE/PVDC | Polyvinyl Chloride / Polyethylene / Polyvinylidene chloride |
| QoL | Quality of Life |
| QP | Qualified Person |
| SAE | Serious Adverse Event |
| SAP | Statistical Analysis Plan |
| SPRM | Selective Progesterone Receptor Modulator |
| SSC | Study Supervisory Committee |
| SUSAR | Suspected Unexpected Serious Adverse Reaction |

| | |
|---|---|
| UAE | Uterine Artery Embolization |
| UFS-QoL | Uterine Fibroid Symptom and health-related quality of life questionnaire |
| US | United States (of America) |
| VAS | Visual Analog Scale |
| WHO | World Health Organisation |

# SYNOPSIS

**Study Title:** A Phase III, multicentre, extension study investigating the efficacy and safety of repeated intermittent 3-month courses of open-label administration of ulipristal acetate, in subjects with symptomatic uterine myomas and heavy uterine bleeding.

| **Code/Name PregLem Investigational Drug:** PGL4001/ Ulipristal acetate | **Phase of Development:** III |
|---|---|

**Objectives:**

**Primary objective:**

- To assess the treatment satisfaction of patients receiving repeated intermittent ulipristal acetate treatment courses using a Global Study Treatment Satisfaction Questionnaire.

**Secondary objectives:**

Efficacy:

- To assess the treatment satisfaction of patients receiving repeated intermittent ulipristal acetate treatment courses using a Global Study Treatment Satisfaction Questionnaire (at additional time points)
- To assess quality of life (measured with EQ-5D and UFS-QoL questionnaires) and pain (using VAS).
- To assess the sustained efficacy of repeated intermittent 3-month treatment courses of daily administration of ulipristal acetate on myoma size.
- To assess uterine bleeding characteristics (using PBAC) at re-start of treatment at the beginning of the study in patients who have already completed 4 previous courses of ulipristal acetate treatment.

Safety:

- To assess the long-term safety of repeated intermittent 3-month administration of ulipristal acetate, specifically with regards to the endometrium and the occurrence of adverse events.

Exploratory:

- To assess the incidence of PAEC, 10-18 days after the first menstruation following the last treatment course with ulipristal acetate.
- To assess the incidence and type of surgery for uterine myomas during the study period

**Endpoints:**

**Primary endpoints:**

- Myoma symptom control assessed with a Global Study Treatment Satisfaction Questionnaire at visit II (Average score of the first 3 questions)
- Myoma symptom control assessed with a Global Study Treatment Satisfaction Questionnaire at visit III (Average Score of the first 3 questions).

**Secondary endpoints:**

Efficacy:

- Myoma symptom control assessed with a Global Study Treatment Satisfaction Questionnaire at visit I (Average score of the first 3 questions).
- Myoma symptom control assessed with a Global Study Treatment Satisfaction Questionnaire at visit IV (Average score of the first 3 questions).
- Myoma symptom control assessed with a Global Study Treatment Satisfaction Questionnaire at visits I, II, III and IV (Individual component scores).
- Change from visit 2 of Pearl III (PGL09-026) and visit F of Pearl III extension (PGL09-027) to visits II, III and IV in quality of life (using EQ-5D and UFS-QoL questionnaires) and in pain (using VAS).
- Change from visit 1 of Pearl III (PGL09-026) and visit F of Pearl III extension (PGL09-027)) to visits II, III and IV in myoma size, measured on the three largest myomas identified at visit 1 of PGL09-026, by transvaginal ultrasound.
- Change from the first menstruation at the start of Pearl III (PGL09-026) study to the first menstrual bleeding at re-start of the first ulipristal acetate treatment course (in PGL11-024) in strength of the bleeding, using the Pictorial Bleeding Assessment Chart (PBAC).

Safety:

- Number and proportion of subjects experiencing treatment-emergent adverse events including patient-reported adverse events and clinically significant changes in the parameters listed below:
  - Laboratory parameters (haematology, chemistry, lipids).

  - Change from visit F of Pearl III extension (PGL09-027) to visits II, III and IV in endometrial thickness assessed by transvaginal ultrasound.
  - Visit III endometrium biopsy (i.e. hyperplasia, adenocarcinoma).

Exploratory:

- Frequency of PAEC observed in the endometrial biopsy, on day 10-18 after the first menstruation following the last treatment course with ulipristal acetate.
- Proportion of subjects having surgery or any invasive procedure for myoma treatment at any time during the study (type of surgery/ procedure performed).

**Study Design:**

This is a phase III, multicentre, long-term open-label extension of the phase III study: Pearl III extension (PGL09-027). During Pearl III (PGL09-026) and subsequent Pearl III extension (PGL09-027), patients have been exposed to a total of 4 cycles of daily 3month open-label treatment with ulipristal acetate 10mg before entering the proposed study Pearl extension 2 (PGL11-024).

This proposed study consists of 4 further consecutive courses of 3 months (84 days) open label ulipristal acetate 10mg once daily treatment each separated by a drug free period.

The first treatment course in this study will be started at start of the first menstruation following visit I.

The subsequent 2$^{nd}$, 3$^{rd}$ and 4$^{th}$ treatment courses will be started at start of second menses after end of the previous treatment course.

Subjects having no return of menses for 6 months during a drug free period will be withdrawn.

**Study Population:**

The target population is composed of women of reproductive age, with moderate to severe symptomatic uterus myoma(s). All subjects will have completed the total of 4 treatment courses of Pearl III (PGL09-026) and its extension study (PGL09-027).

All subjects having completed Pearl III extension study (PGL09-027) without significant protocol deviations will be eligible to participate in this study.

About 70-90 subjects from Pearl III extension are expected to participate in this study.

**Main Eligibility Criteria:**

**Inclusion Criteria**

To be eligible for inclusion into this study, the subjects must **fulfill all** of the following criteria:

1. Provision of written informed consent prior to any study-related procedures.

2. Subject completed visit F – Follow-up of Pearl III extension study (PGL09-027) - without significant deviations.
3. Subject has no contra-indication to enter the long term extension study, based on the investigator’s judgment.
4. Females of childbearing potential are advised to practice a non-hormonal method of contraception among one of the following:
    a. Sexual abstinence
    b. Diaphragms
    c. Condom or having a partner with a vasectomy with either confirmed azoospermia or performed at least 6 months prior to the study.

**Exclusion Criteria**

To be eligible for inclusion in this study the subjects must **not** meet **any** of the following criteria:

1. Subject has a history of uterus surgery (e.g. hysterectomy, myomectomy) or uterine artery embolization in Pearl III extension (PGL09-027) or afterwards that would interfere with the study assessments.
2. Subject has taken or is likely to require treatment during the study with drugs that are not permitted by the study protocol: progestins (systemic or progestin releasing intra-uterine system), oestrogens, GnRH-Agonists/Antagonists, hormonal contraceptives, systemic glucocorticoids (oral and injectable), and/or treatments with potent inhibitors or inducers of CYP3A4. Wash-out periods prior to Visit I are as follows:
    a. Progestins and Oestrogens: 1 month
    b. GnRH-Agonists/Antagonists: 3 months
    c. Systemic glucocorticoids: 1 month (3 months after depot injections)
    d. CYP3A4 inhibitors or inducers: 2 weeks
3. Subject is lactating, has a positive pregnancy test at study start or is planning a pregnancy during the course of the study.
4. Subject has a current problem with alcohol or drug abuse.
5. Subject has a mental condition rendering her unable to understand the nature, scope and possible consequences of the study, and/or evidence of an uncooperative attitude.
6. Subject has abnormal baseline findings, any other medical condition(s) or laboratory finding that, in the opinion of the investigator, might jeopardise the subject’s safety or interfere with study evaluations.

**Investigational Medicinal Product(s):**

This study includes four treatment courses separated by a drug-free period, until the start of second

menses following the end of the previous treatment course.

Each treatment course is composed of three months (84 days) daily oral treatment with 10mg ulipristal acetate (PGL4001).

**Data Analysis and Statistics:**

No formal hypothesis tests have been pre-specified in the protocol. Descriptive statistics will be determined for all measured as well as derived endpoints. Data will be summarised for all subjects treated during PGL11-024, summarised by visit where applicable. The average score for the Global Study Treatment Satisfaction Questionnaire will be derived as the average scores of questions 1, 2 and 3.

# 1. BACKGROUND INFORMATION

## 1.1. INTRODUCTION TO PGL4001

PGL4001 ulipristal (International Nonproprietary Name (INN) acetate ulipristal is a steroid compound (17α-Acetoxy-11β-(4-N,N-dimethylaminophenyl)-19-norpregna-4,9-diene-3,20-dione) whose main pharmaco-dynamic property is to reversibly block the progesterone receptor in its target tissues and act as a potent, orally active progesterone receptor modulator. It belongs to the class of Selective Progesterone Receptor Modulators (SPRMs).

SPRMs express agonist / antagonist activities of progesterone based on the target tissue and absence/presence of progesterone. The predominant property of SPRMs is a progesterone antagonist activity [1,2]. Uterine myomas contain progesterone receptors and many increase in size in response to an increase in progesterone. In addition, SPRMs partially reduce oestrogen concentrations by inhibiting Follicle-Stimulating Hormone (FSH) and Luteinising Hormone (LH) surges depriving the myoma of its two potent stimulators of growth, progesterone and oestrogen. SPRMs also interact directly with progesterone receptors in the endometrium to induce amenorrhoea in the majority of women. In phase II studies, PGL4001 has shown to efficiently reduce or stop uterine bleeding, alleviate abdominal symptoms, and reduce myoma and uterine volume [3,4].

There is already a European marketing authorization (granted to HRA Pharma) for ulipristal acetate 30mg tablets single dose administration for emergency contraception (Ellaone® EU Marketing Authorization granted 15 May 2009, see http://www.ema.europa.eu/ema/index.jsp?curl=pages/medicines/human/medicines/001027/human_med_000758.jsp&murl=menus/medicines/medicines.jsp&mid=WC0b01ac058001d125&jsenabled=true).

PregLem first developed the compound for benign gynaecological indications including uterine myoma. Two Phase III studies were performed to assess the efficacy, safety, and tolerance of PGL4001 with 5 and 10 mg tablets in the pre-operative treatment of symptomatic uterine myoma. PregLem was granted a Marketing Authorization for Esmya® (PGL4001 5 mg tablets) on 23rd February 2012 in the indication of "*pre-operative treatment of moderate to severe symptoms of uterine fibroids in adult women of reproductive age. The treatment duration is limited to 3 months*". Results of the two Phase III studies were recently published in the New England Journal of Medicine (NEJM)[5,6]

A Phase III open label (PGL09-026) study assessing the safety and efficacy of three months treatment with 10mg dose was completed recently and the database was locked on 02 February 2012. Its long-term extension (PGL09-027) of three additional 3-month treatment courses with PGL4001 10mg is still ongoing.

## 1.2. UTERINE MYOMA

Uterine myomas are benign, monoclonal, hormone-sensitive, smooth muscle tumours of the uterus. They are the most common tumour of the female reproductive tract in pre-menopausal women and

mostly asymptomatic affecting approximately 40% of women between 35 and 55 years. When symptomatic, the main symptoms are heavy uterine bleeding, abdominal pressure, abdominal pain, increased urinary frequency and infertility. Anaemia may occur as a consequence of heavy bleeding. Besides causing physical morbidity, uterine myomas are a frequent cause of significant impairment of Quality of Life (QoL) [7]. They are also a leading cause of hysterectomy [8].

## 1.3. CONVENTIONAL TREATMENT OF UTERINE MYOMA

The current mainstay of symptomatic myoma treatment is surgery. The most common procedure is hysterectomy, but less invasive procedures have been developed especially when the patient wishes to preserve fertility (e.g. myomectomy by laparoscopy, hysteroscopy or laparotomy) or her uterus (e.g. uterine artery embolization (UAE), endometrial ablation). Short term complications and long term consequences of a hysterectomy can be significant, e.g. in the long term a significant increase of moderate and severe urinary incontinence has been reported [9].

Myomectomy maintains possible fertility but does not prevent the reoccurrence of fibroids.

Endometrial ablation is a treatment option for patients who do not wish to maintain fertility, if the dominant symptom is bleeding and the uterus volume is relatively small.

UAE is a lighter procedure than a hysterectomy and requires a shorter hospital stay, however there is a risk that myomas will reoccur and require further intervention to control symptoms. There is also a significant risk that a later hysterectomy will have to be performed due to complications arising from UAE. Pregnancies after UAE have been reported, however careful counselling of patients is needed as fertility may be seriously impacted.

Gonadotropin-Releasing Hormone (GnRH) agonists have been shown to be effective in reducing myoma-related bleeding, correcting anaemia when given concomitantly to iron therapy, reducing abdominal symptoms and reducing myoma as well as uterine volume ([10], [11]). Although registered for the pre-operative treatment of symptomatic myomas, the use of GnRH agonists has been relatively limited due to their sub-optimal side effect profile caused by suppression of oestrogen to castration levels resulting in florid symptoms of menopause such as hot flushes, depression, mood swings, loss of libido, nervousness and vaginitis (see Lupron label @ http://www.lupron.com/). In addition, GnRH agonists have a negative impact on bone mineralisation, with an estimated loss of 2.7% in bone mineral density (BMD) after three months of treatment. The loss of BMD is only partially and slowly reversible. As a consequence of the adverse safety profile, the use of GnRH agonist is limited to 3-6 months in this indication.

PGL4001 (5mg once daily) is licensed in the European Union in the pre-operative treatment of moderate to severe symptoms of uterine fibroids in adult women of reproductive age. The treatment duration is limited to 3 months.

There is currently no satisfactory medical long-term treatment for uterine myoma.

## 1.4. SUMMARY OF PRE-CLINICAL STUDIES

### 1.4.1. Summary of Pharmacology

Ulipristal acetate shows potent affinity for the progesterone and glucocorticoid receptors and acts mostly as an antagonist at both sites. In a range of in vitro pharmacological studies, its actions were compared to those of a reference SPRM i.e. mifepristone. Although ulipristal acetate and mifepristone showed similar affinity for progesterone and glucocorticoid receptors, this similarity was not reflected in measures of functional activity. Both compounds had similar potency in antagonising the progesterone-receptor mediated R5020 stimulation of endogenous alkaline phosphatase or transcriptional activity in T47D-CO cells, but the activity of ulipristal acetate in antagonising the glucocorticoid-receptor mediated dexamethasone stimulation of transcriptional activity in HepG2 cells was approximately 10-fold lower than that of mifepristone. Furthermore, while mifepristone functioned as an active antagonist in HeLa cells, ulipristal acetate functioned as a competitive antagonist since it showed high affinity binding to the receptor but the resultant ligand receptor complex was unable to bind DNA. In studies examining the localization of the glucocorticoid receptor ligand complex in COS-1 cells, mifepristone promoted an efficient translocation of the glucocorticoid receptor to the nucleus whereas ulipristal acetate was only partially active in this regard.

Regarding functional effects at the progesterone receptor, ulipristal acetate displayed agonist progesterone effects on cell growth via both PR isoforms and exerted antagonist activities on adhesion via PR-B. These dual properties were also displayed at the gene expression level – the compounds acted as agonists on cell cycle genes but exhibited antagonistic effects on cell adhesion genes.

Effects on human fibroid cells through inhibition of proliferation (via regulation of cell cycle genes), induction of apoptosis via regulation of caspase, PARP and Bcl-2, action on angiogenic growth factor and receptors were observed in vitro along with other non-proliferative effects in another cell model. These effects were not observed in myometrial cells, suggesting that ulipristal affects cell proliferation and apoptosis specifically in the cells of uterine fibroids. In a clinical setting, this may trigger a reduction of fibroid volume via suppression of cell proliferation and cell survival.

Specific effects on the endometrium were observed during in vivo studies, with ulipristal acetate acting through suppression of estrogen dependent endometrial proliferation and mitotic activity in response to progesterone in the estrogen-primed rabbit uterus. In women, this may stop uterine bleeding by reducing proliferation of the endometrium and inducing a mixed proliferative-secretory pattern causing amenorrhea. It is of note that clinical signs such as amenorrhea were also observed in toxicology work in the cynomolgus monkey. Ulipristal acetate was metabolized by CYP3A4 isoenzyme mediated oxidation to yield predominantly the mono-N-demethylated (PGL4002) and di-N-demethylated (PGL4004) derivatives. PGL4002 had a similar hormonal receptor binding profile to ulipristal acetate itself, although less potent, and PGL4004 was less potent still. In vivo, PGL4002 was approximately 4-fold less active than ulipristal acetate in the anti-Clauberg test after oral dosing whilst PGL4004 did not show any activity in the anti-McGinty test.

### 1.4.2. Pharmacokinetics

Ulipristal acetate was rapidly and completely absorbed after oral dosing. It is highly bound to plasma proteins (96.7-99.5%) across a range of species. Tissue distribution of radioactivity was widespread and a study in pigmented rats showed selective binding in pigmented tissues as compared to albino animals. Due to these findings, phototoxicity of ulipristal acetate was investigated in vitro and results indicated that the risk of phototoxicity associated with use of ulipristal acetate was negligible. The major route of biotransformation of ulipristal acetate was the oxidative demethylation of the aniline moiety of ulipristal acetate to N-monodemethylated ulipristal acetate (PGL4002) and subsequently to N-didemethylated ulipristal acetate (PGL4004) via CYP3A4 in the liver. In vivo metabolism investigations of 14C-ulipristal acetate in women identified PGL4002 and to a much lower extend PGL4004 as the major circulating metabolites. The metabolic profile of women closely resembled the monkey study due to other minor ulipristal acetate derivatives seen only in monkey and women but not in the rat. Major circulating human metabolites were observed in both rat and monkey indicating both toxicology species were appropriate. Based on the metabolite profiling analyses during the mass balance studies in rat, monkey and women, adequate safety cover for circulating metabolites was established in rats and monkeys compared to that expected clinically.

Excretion of radioactivity was predominantly faecal in both rats and monkeys with bile shown to play a major role in rats. This was similar to findings in women (PGL-09-015). Ulipristal acetate was also shown to be excreted in milk, but plasma levels could not be measured in pups.

Pharmacokinetic interaction studies in vitro indicated that ulipristal acetate and PGL4002 did not induce CYP1A2 or CYP3A4 activity in human hepatocytes or participate in inhibitory drug-drug interactions involving the major CYP metabolic pathways at clinically relevant concentrations. Ulipristal acetate and PGL4002 were not shown to be a P-glycoprotein (P-gp) substrate. However, ulipristal acetate was considered to be a potent P-gp inhibitor in Caco-2 cells. However a recently completed clinical interaction study with the P-gp substrate fexofenadine indicates no significant effect of ulipristal acetate (10mg) on the pharmacokinetics of fexofenadine.

For more information on pharmacokinetics, see section 2.3.2 of the PGL4001 investigator's brochure (IB).

### 1.4.3. Safety Pharmacology

A battery of safety pharmacology studies was conducted in accordance with ICH guidelines, supported by pharmacokinetic studies at the same dose levels (5, 25 and 125 mg/kg). Ulipristal acetate demonstrated no particular preclinical safety signal regarding cardiovascular, central nervous system and respiratory function at exposure largely exceeding the likely maximum exposure at the intended therapeutic dose of 5 and 10mg.

For more details, please refer to the IB.

### 1.4.4. Toxicology

Toxicology studies with ulipristal acetate were dominated by the consequences of its pharmacological activity. The observed effects of ulipristal acetate arose from disruption of the hypothalamic-pituitary-adrenal axis and reproductive systems which manifested as changes in the pituitary, adrenal and mammary glands as well as in the ovary and uterus, together with increases in serum levels of corticosterone/cortisol and prolactin. Overall, the toxicological profile of ulipristal acetate emerging from the repeated-dose toxicity studies appears to be a consequence of its pharmacological activity as a progesterone and glucocorticoid antagonist. A level of 25 mg/kg/day ulipristal acetate was shown to be a NOAEL (monkey) or as showing no overt toxicity (rat) and gave exposure (based on AUC) of 0.511 and 29.78 h.µg/mL, respectively.

Reproductive toxicity studies were especially limited in dose by the antiprogesterone effects of ulipristal acetate preventing the maintenance of pregnancy. In rat and rabbit embryo-foetal studies, interruptions of pregnancy were observed at ≥ 1 mg/kg/day. However, no indication of any teratogenic effect was observed. Rat peri and post-natal development work showed increased or total pre-natal loss at ≥ 0.3 mg/kg/day in the rat along with evidence of premature parturition. However, no effects on developmental or behavioural effects were seen.

No evidence of genotoxicity potential was found for ulipristal acetate as evaluated in a battery of in vitro and in vivo assays. For more details concerning toxicology, please refer to the IB.

## 1.5. SUMMARY OF CLINICAL STUDIES

At the time of this protocol finalization, clinical experience in completed studies with PGL4001 tablets includes 4843 healthy volunteers and patients exposed to a single dose of up to 200 mg, and 621 exposed to multiple doses testing 5mg or higher daily doses for more than 12 weeks. Thus, so far in multiple dose administration studies PGL4001 has shown a good safety profile with a very low dropout rate due to adverse events. For more details please refer to the PGL4001 IB.

In addition, this clinical phase III trial aims to establish the efficacy and safety of PGL4001 in the treatment of uterine myoma in approximately 70-90 subjects (that are anticipated to complete Pearl III extension (PGL09-027)) treated with PGL4001 10 mg for four further consecutive courses of 3 months.

Long-term treatment with Progesterone receptor modulators (PRM) results in changes to the endometrium. Current World Health Organisation (WHO) classification is not suitable to describe the non-physiological endometrial features observed during PRM treatment. In an expert meeting organized by the NIH on the endometrial effects of PRMs, a panel of pathologists was convened to evaluate endometrial changes associated with a minimum of three months of treatment with PRMs ([12]). The panel has designated these changes as PRM-associated endometrial changes (PAEC) ([13]). In the phase III program, endometrium biopsies are analysed by three independent pathologists experienced in evaluation of PAECs, and assessed with an appropriate rating scale developed in cooperation with four pathologists having participated to the NIH expert meeting. This rating scale is based on the US Food and Drug Administration (FDA) guideline

(http://www.fda.gov/downloads/Drugs/DrugSafety/InformationbyDrugClass/UCM135338.pdf) and includes both the standard WHO classification and the new PAEC classification. The same scale was used in both Phase III studies supporting the registration for pre-operative use (Studies PGL07-021 and PGL07-022).

## 1.6. RATIONALE FOR THE CURRENT STUDY

Ulipristal acetate 5mg dose was developed in the indication of pre-operative treatment of symptomatic uterine myoma and received Marketing Authorization in the European Economic Area on 23rd February 2012. After completing one three-month course of treatment, some subjects may decline to have surgery as they feel better or surgery may have to be delayed for a variety of reasons. In such circumstances it is important to confirm that ulipristal acetate maintains its efficacy on fibroid size and menstrual bleeding and that it is a safe medical treatment for intermittent use. Further it is of interest to demonstrate that any PAEC on the endometrium, which occurred in some subjects during ulipristal acetate treatment, are reversible and that after repeated cycles with ulipristal acetate, the endometrium resumes a physiological appearance after one or a few menstrual cycles without treatment.

In two Phase III studies PGL07-021 (PEARL I) and PGL07-022 (PEARL II), both the 5mg and 10mg doses were highly effective in improving the symptoms of myoma. A dose of 5 mg is the lowest effective dose to control the excessive bleeding of uterine myoma. From a purely clinical point of view, the 10 mg dose was associated with possible superior efficacy, especially in terms of proportions of patients who achieved amenorrhea. When taking in consideration the size of the database of exposed subjects, PregLem decided to take a conservative approach and to recommend the 5 mg dose for initial submission for the pre-operative treatment of uterine myoma whilst continuing to assess the tolerance and safety of ulipristal acetate 10 mg by conducting an additional phase III trial (PEARL III (PGL09-026) and Pearl III extension (PGL09-027)). In these studies, it was further investigated whether a 10 day course of a synthetic progestin may have an effect on the time needed for the reversibility of the PAEC. As the main activity of the PGL4001 is a progesterone antagonism, a 10-day course of progestins after administration of progesterone receptor modulators (PRMs) was thought to efficiently reduce the anti-progestational effect on the endometrium and facilitate scheduling the menstruation without countering the beneficial effect of PGL4001 on uterine myoma reduction.

In addition, for the development of the long-term intermittent use, PregLem is further investigating the efficacy and safety of the 5 and 10mg doses of PGL4001 in approximately 400 additional subjects for up to 12 weeks repeated four times (PEARL IV (PGL11-006)).

PregLem decided to further investigate the efficacy and safety of repeated intermittent treatment courses of ulipristal acetate over four additional 3-month courses. Patients who complete Pearl III extension (PGL09-027) study will be given the opportunity to enter this long-term open-label extension study in which four additional consecutive 3-month courses of ulipristal acetate treatment in the same strength as previously received will be offered. As early results from PGL09-026 demonstrated that the effect of the progestin course on the PAEC reversibility is only marginal, and PAEC reverses quickly with and without progestin, continuation with the progestin administration in further clinical studies was not deemed necessary.

The main objective of this study is to assess the sustained efficacy of intermittent 3- month treatment courses on treatment satisfaction of patients, on quality of life and pain, myoma size and uterine bleeding.

### 1.7. SUMMARY OF OVERALL RISK AND BENEFITS

Nonclinical and clinical studies have demonstrated that ulipristal acetate is a potent, orally-active progesterone receptor modulator that primarily antagonizes progesterone action in target tissues. Non-clinical testing supports testing in clinics beyond 26 weeks.

Ulipristal acetate is well-tolerated in animals at doses that well exceed the dose under investigation in humans and does not show potential for genotoxicity or reproductive toxicity.

A single dose of up to 200mg and a daily dose of up to 20 mg in women have been shown to be well tolerated.

Overall tolerance in clinical trials including Phase III trials with administration of 5 mg/day and 10 mg/day during 3 months was good, and the rate of the occurrence of any adverse events did not differ significantly in patients treated with 5 mg/day and 10 mg/day or placebo for 3 months. Headache and pooled data for breast pain, breast discomfort, or breast tenderness were the most common adverse events in the ulipristal acetate groups.

A Study supervisory committee is reviewing the safety in Pearl III extension study (PGL09-027) at regular meetings and so far, no new safety signal or reason of concern has emerged from this on-going study.

Overall, the ongoing benefit risk assessment supports considering long-term repeated intermittent treatment with PGL4001.

## 2. OBJECTIVES

### 2.1. PRIMARY OBJECTIVE:

- To assess the treatment satisfaction of patients receiving repeated intermittent ulipristal acetate treatment courses using a Global Study Treatment Satisfaction Questionnaire.

### 2.2. SECONDARY OBJECTIVE:

Efficacy:

- To assess the treatment satisfaction of patients receiving repeated intermittent ulipristal acetate treatment courses using a Global Study Treatment Satisfaction Questionnaire (at additional time points)

- To assess quality of life (measured with EQ-5D and UFS-QoL questionnaires) and pain (using VAS).
- To assess the sustained efficacy of repeated intermittent 3-month treatment courses of daily administration of ulipristal acetate on myoma size.
- To assess uterine bleeding characteristics (using PBAC) at re-start of treatment at the beginning of the study in patients who have already completed 4 previous courses of ulipristal acetate treatment.

Safety:

- To assess the long-term safety of repeated intermittent 3-month administration of ulipristal acetate, specifically with regards to the endometrium and the occurrence of adverse events.

### 2.3. EXPLORATORY OBJECTIVES:

- To assess the incidence of PAEC, 10-18 days after the first menstruation following the last treatment course with ulipristal acetate.
- To assess the incidence and type of surgery on uterine myomas during the study period.

## 3. ENDPOINTS

### 3.1. PRIMARY ENDPOINTS:

- Myoma symptom control assessed with a Global Study Treatment Satisfaction Questionnaire at visit II (Average score of the first 3 questions).
- Myoma symptom control assessed with a Global Study Treatment Satisfaction Questionnaire at visit III (Average score of the first 3 questions).

### 3.2. SECONDARY ENDPOINTS:

Efficacy:

- Myoma symptom control assessed with a Global Study Treatment Satisfaction questionnaire at visit I (Average score of the first 3 questions).
- Myoma symptom control assessed with a Global Study Treatment Satisfaction questionnaire at visit IV (Average score of the first 3 questions).
- Myoma symptom control assessed with a Global Study Treatment Satisfaction questionnaire at visits I, II, III and IV (Individual component scores).

- Change from visit 2 of Pearl III (PGL09-026) and visit F of Pearl III extension (PGL09-027) to visits II, III and IV in quality of life (using EQ-5D and UFS-QoL questionnaires) and in pain (using VAS).

- Change from visit 1 of Pearl III (PGL09-026) and visit F of Pearl III extension (PGL09-027) to visits II, III and IV in myoma size, measured on the three largest myomas identified at visit 1 of PGL09-026, by transvaginal ultrasound.

- Change from the first menstruation at the start of Pearl III (PGL09-026) study to the first menstrual bleeding at re-start of the first ulipristal acetate treatment course (in PGL11-024) in strength of the bleeding, using the Pictorial Bleeding Assessment Chart (PBAC).

Safety:

- Number and proportion of subjects experiencing treatment-emergent adverse events including patient-reported adverse events and clinically significant changes in the parameters listed below:

    - Laboratory parameters (haematology, chemistry, lipids).

    - Change from visit F of Pearl III extension (PGL09-027) to visits II, III and IV in endometrial thickness assessed by transvaginal ultrasound.

    - Visit III endometrium biopsy (i.e. hyperplasia, adenocarcinoma).

### 3.3. EXPLORATORY ENDPOINTS:

- Frequency of PAEC observed in the endometrial biopsy, on day10-18 after the first menstruation following the last treatment course with ulipristal acetate.

- Proportion of subjects having surgery or any invasive procedure for myoma treatment at any time during the study (type of surgery/ procedure performed).

## 4. STUDY DESIGN

This is a phase III, multicentre, long-term open-label extension of the phase III study: Pearl III extension (PGL09-027). During Pearl III (PGL09-026) and subsequent Pearl III extension (PGL09-027), patients have been exposed to a total of 4 cycles of daily 3-month open-label treatment with ulipristal acetate 10mg before entering the proposed study Pearl extension 2 (PGL11-024).

This proposed study consists of 4 further consecutive courses of 3 months (84 days), open label, ulipristal acetate 10mg once daily treatment each separated by a drug free period.

The first treatment course in this study will be started at start of the first menstruation following visit I.

The subsequent 2nd, 3rd and 4th treatment courses will be started at start of second menses after end of the previous treatment course.

Subjects having no return of menses for 6 months during a drug free period will be withdrawn.

**Figure 1: Pearl extension 2 design**

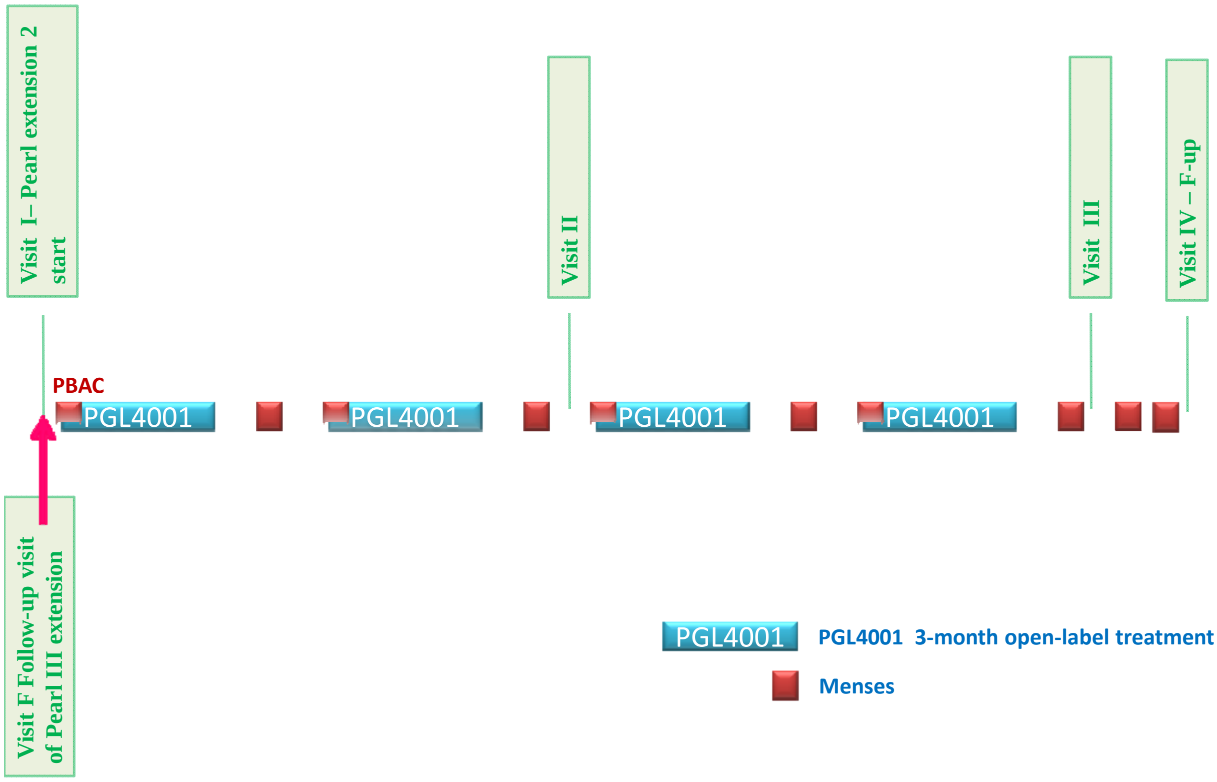


# 5. STUDY POPULATION

## 5.1. SUBJECTS

The target population is composed of women of reproductive age, with moderate to severe symptomatic uterus myoma(s). All subjects will have completed the total of 4 treatment courses of Pearl III (PGL09-026) and its extension study (PGL09-027).

All subjects having completed Pearl III extension study (PGL09-027) without significant protocol deviations will be eligible to participate in this study.

About 70-90 subjects from Pearl III extension are expected to participate in this study.

## 5.2. ENTRY CRITERIA

### 5.2.1. Inclusion Criteria

To be eligible for inclusion into this study, the subjects must **fulfill all** of the following criteria:

1. Provision of written informed consent prior to any study-related procedures.
2. Subject completed visit F – Follow-up of Pearl III extension study (PGL09-027) - without significant deviations.
3. Subject has no contra-indication to enter the long term extension study, based on the investigator's judgment.
4. Females of childbearing potential are advised to practice a non-hormonal method of contraception among one of the following:
    a. Sexual abstinence
    b. Diaphragms
    c. Condom or having a partner with a vasectomy with either confirmed azoospermia or performed at least 6 months prior to the study.

### 5.2.2. Exclusion Criteria

To be eligible for inclusion in this study the subjects must **not** meet **any** of the following criteria:

1. Subject has a history of uterus surgery (e.g. hysterectomy, myomectomy) or uterine artery embolization in Pearl III extension (PGL09-027) or afterwards that would interfere with the study assessments.
2. Subject has taken or is likely to require treatment during the study with drugs that are not permitted by the study protocol: progestins (systemic or progestin releasing intra-uterine system), oestrogens, GnRH-Agonists/Antagonists, hormonal contraceptives, systemic glucocorticoids (oral and injectable), and/or treatments with potent inhibitors or inducers of CYP3A4. Wash-out periods prior to Visit I are as follows:
    a. Progestins and Oestrogens: 1 month
    b. GnRH-Agonists/Antagonists: 3 months
    c. Systemic glucocorticoids: 1 month (3 months after depot injections)
    d. CYP3A4 inhibitors or inducers: 2 weeks
3. Subject is lactating, has a positive pregnancy test at study start or is planning a pregnancy during the course of the study.
4. Subject has a current problem with alcohol or drug abuse.

5. Subject has a mental condition rendering her unable to understand the nature, scope and possible consequences of the study, and/or evidence of an uncooperative attitude.
6. Subject has abnormal baseline findings, any other medical condition(s) or laboratory finding that, in the opinion of the investigator, might jeopardise the subject's safety or interfere with study evaluations.

# 6. STUDY PROCEDURES AND ASSESSMENTS

## 6.1. GENERAL INSTRUCTIONS

Before the start of the study, each subject will be provided with a subject information sheet giving details of the IMP, procedures and potential risks involved. They will be instructed that they are free to obtain further information from the Investigator at any time and that they are free to withdraw their consent and to discontinue their participation in the project at any time without prejudice.

Each potentially eligible subject will also be informed verbally of the study's objectives and overall requirements. Prior to conducting any of the study procedures, the Investigator or qualified delegate will explain the study fully. If the subject is willing to participate in the study, she will be requested to give written informed consent after being given sufficient time to consider her participation and the opportunity to ask for further details. The consent form will be signed and personally dated by both the subject and the investigator or qualified delegate. The original will be kept by the Investigator in the confidential investigator file, and one signed copy will be given to the subject. Although nursing staff may be involved in describing the trial to a subject, the investigator/sub-investigator must participate in discussions with the subject and sign and personally date the informed consent form.

Subjects will be asked if they authorise the investigator to notify their general practitioner of their participation in the trial.

Each subject will keep the subject identifier (ID) that was provided in the PGL09-027 study. This identifier consists of a 2-digit centre number (Centre No.) and a two-digit subject number (Subject No.).

During the whole study, the subject will be identified using the subject identification number (ID) for all documentation and discussion. The Subject Identification Number assigned to a subject in this way must only be used for that subject.

The Case Report Form for this study will be electronic. However the questionnaires (Global study treatment satisfaction questionnaire, UFS-QoL, EQ-5D and VAS for pain) will be completed by the subject at site on paper. Furthermore, subjects will complete paper diaries to collect medication intake and PBAC. The independent pathologists reading the endometrium biopsies will be given the option to complete the biopsy CRF pages on paper or electronically.

The ulipristal acetate treatment is given to all subjects in an open-label manner. Upon confirmation of eligibility at visit I, the investigator or delegate will enrol the subject in the study, give the subject two

quarterly kits of ulipristal acetate treatment and will stick the tear-off labels bearing the kit numbers on the subject log.

## 6.2. OUTLINE OF STUDY PROCEDURES AND ASSESSMENTS

### 6.2.1. Visit I

Visit I will take place approximately three months after the last treatment course in PGL09-027 study (or exceptionally more due to delayed start-up of the study).

Generally, the day of visit I will coincide with visit F (Follow-up visit of Pearl III extension (PGL09-027)). However, at beginning of the study exceptionally a longer time gap may be accepted to allow patients having already finished study PGL09-027 to enter the study.

The subject will be informed of the study's objectives and overall requirements, and written informed consent will be obtained before performing any study-specific procedures.

Upon signing of the informed consent, the following assessments/procedures will be performed to establish the subject's eligibility for the trial:

- Confirm inclusion/exclusion criteria
- Record medical history
- Vital signs
- For subjects having a gap of more than 3 months between visit F of PGL09-027 and the visit I of this study, a gynaecological examination including breast examination, will be performed
- Urine pregnancy test
- Record Global Study Treatment Satisfaction Questionnaire
- Record concomitant medication
- For subjects having a gap of more than 3 months between visit F of PGL09-027 and the visit I of this study, a transvaginal ultrasound of ovaries, uterine volume, total volume of the three largest myomas (identified at visit 1 in PGL09-026) and endometrium thickness, will be performed
- For subjects for whom visit I is on a different date than visit F (Follow-up visit in study PGL09-027), an endometrial biopsy is to be performed in the following three situations:

  a) visit E endometrial biopsy indicated the need to perform a visit F biopsy, however visit F biopsy could not be obtained

  b) visit F biopsy was obtained however evaluated as "not adequate"

c) for all subjects having a gap of more than 3 months between visit F of PGL09-027 and the visit I of this study.

If the subject is eligible and has consented to the study, then

- Dispense PGL4001 (2 quarterly kits for the first and second 3-month treatment courses)
- Dispense subject diaries (medication intake and PBAC) and instruct subjects how to complete them
- Dispense standardised tampons and sanitary towels for the PBAC assessment. Towels and sanitary towels will be provided to the sites by the sponsor (or delegate).

Subject will be instructed to begin the first 3-month (84 days) treatment course on the first day of her next menstrual bleeding following visit I.

Subject will be asked to record their bleeding pattern using the Pictorial Bleeding Assessment Chart (PBAC) (see APPENDIX B) for the first 8 days of the first menstruation at restart of the first treatment course.

Subject will be instructed to begin the second 3-month (84 days) treatment course the first day of the second menstruation following the end of the previous treatment course.

Subject must be instructed that once she has finished the two 3-month treatment courses, she must call the site on the first day of her menstruation following the end of the second treatment course to schedule the next visit II (10 to 18 days after the start of menstruation).

Subjects will be instructed to bring their used and if applicable their unused blisters and diary card to the next visit II.

**If menstrual periods have not returned for 6 months during a drug free period, the subject will be asked to attend an early termination visit (see section 6.2.5). During this visit, blood samples for FSH and E2 will be assessed.**

### 6.2.2. Visit II

This visit will be scheduled 10 to 18 days after the first day of menstruation following the end of the second 3-month treatment course.

During the visit, the following assessments/procedures will be performed:

- Vital signs
- Gynaecological examination including breast examination
- Urine pregnancy test
- Dispense PGL4001 (2 quarterly kits for the third and fourth 3-month treatment courses)

- Study drug compliance check
- Collect subject diary and dispense the next diary
- Record Global Study Treatment Satisfaction Questionnaire
- Record UFS Quality of Life
- Record EQ-5D Quality of Life
- Record VAS for pain
- Transvaginal ultrasound of ovaries, uterine volume, total volume of the three largest myomas (identified at visit 1 in PGL09-026) and endometrium thickness
- Perform endometrium biopsy if endometrium thickness at transvaginal ultrasound >18mm or if there are any other clinical reasons to do so
- Record any new or change in concomitant medication since the previous visit or new one
- Record adverse events

Subject will be instructed to begin the third 3-month (84 days) treatment course on the first day of her next menstrual bleeding following visit II.

Subject will be instructed to begin the fourth 3-month (84 days) treatment course on the first day of the second menstruation following the end of the previous treatment course.

Subject must be instructed that once she has finished the fourth 3-month treatment course, she must call the site on the first day of her menstruation following the end of the previous treatment course to schedule the next visit III (10 to 18 days after the start of menstruation). The appointment should be in the morning if possible for blood samples. As blood lipids will also be assessed, the subject will need to have a light meal in the evening and be fasting overnight prior to the visit III.

Subjects will be instructed to bring their used and if applicable their unused blisters and diary card to the next visit III.

**If menstrual periods have not returned for 6 months during a drug free period, the subject will be asked to attend an early termination visit (see section 6.2.5). During this visit, blood samples for FSH and E2 will be assessed.**

### 6.2.3. Visit III – Post-treatment period

This visit will be scheduled 10 to 18 days after the first day of menstruation following the end of the fourth 3-month treatment course. Subjects will need to be fasting overnight for this visit.

During the visit, the following assessments/procedures will be performed:

- Body weight

- Vital signs
- Gynaecological examination (including breast examination)
- Urine pregnancy test
- Study drug compliance check, all blisters should have been returned
- Collect subject diary
- Record Global Study Treatment Satisfaction Questionnaire
- Record UFS Quality of Life
- Record EQ-5D Quality of Life
- Record VAS for pain
- Transvaginal ultrasound of ovaries, uterine volume, total volume of the three largest myomas (identified at visit 1 in PGL09-026) and endometrium thickness
- Perform endometrium biopsy
- Record any new or change in concomitant medication since the previous visit or new one
- Record adverse events
- Blood samples for haematology, chemistry and lipids

Subject must be instructed that the next visit IV should take place 10-18 days after start of third menstruation following end of the fourth 3-month treatment courses **or** 3 months after last dose of the fourth 3-month treatment course (at least ten days after the last menstruation, if applicable), whatever arrives first.

#### 6.2.4. Visit IV – Follow-up visit

Visit IV should take place 10-18 days after start of third menstruation following end of the fourth 3-month treatment course **or** 3 months after last dose of the fourth 3-month treatment course (at least ten days after the last menstruation, if applicable), whatever arrives first.

During the visit, the following assessments/procedures will be performed:

- Vital signs
- Gynaecological examination including breast examination
- Urine pregnancy test
- Record Global Study Treatment Satisfaction Questionnaire

- Record UFS Quality of Life
- Record EQ-5D Quality of Life
- Record VAS for pain
- Transvaginal ultrasound of ovaries, uterine volume, total volume of the three largest myomas (identified at visit 1 in PGL09-026) and endometrium thickness
- Perform endometrium biopsy, if endometrium histology differs from “benign physiologic” endometrium in the biopsy performed at visit III
- PAP smear (if no PAP smear performed in the past 12 months)
- Record any new or change in concomitant medication since the previous visit or new one
- Record adverse events
- Complete end of study page

#### 6.2.5. Early termination visit

If the subject is withdrawn from the study at any point, she must complete an early termination visit. This visit, which includes the same assessments as visit IV, will take place 10-18 days after the beginning of menses, so that an endometrium biopsy can be taken, if applicable.

- Vital signs
- Gynaecological examination including breast examination
- Urine pregnancy test
- Record Global Study Treatment Satisfaction Questionnaire
- Record UFS Quality of Life
- Record EQ-5D Quality of Life
- Record VAS for pain
- Transvaginal ultrasound of ovaries, uterine volume, total volume of the three largest myomas (identified at visit 1 in PGL09-026) and endometrium thickness (if applicable)
- Perform endometrium biopsy, if applicable
- PAP smear (if no PAP smear performed in the past 12 months)
- Record any new or change in concomitant medication since the previous visit or new one
- Record adverse events

- Blood samples for FSH and E2 if the reason for the early termination visit is that no menstruation occurred for 6 months during a drug free period
- Study drug compliance check
- Complete end of study page, including reason for withdrawal

## 6.3. EFFICACY OBSERVATIONS AND MEASUREMENTS

### 6.3.1. Quality of life

Subjects will complete one paper Global Study Treatment Satisfaction Questionnaire (see APPENDIX C) at each visit to assess their treatment satisfaction.

Subjects will complete paper UFS Quality of Life (see APPENDIX D) and EQ-5D questionnaires (see APPENDIX E) at visits II, III and IV (and Early Termination, if applicable) to determine maintained Quality of life.

There are two Baseline scores for the Quality of Life questionnaires: Baseline UFS-Qol and EQ-5D scores will be the scores recorded at visit 2 (Baseline I) of Pearl III (PGL09-026) and at visit F (Baseline II) of Pearl III extension (PGL09-027).

### 6.3.2. Pain

A Visual Analog Scale (VAS) (see APPENDIX F) evaluating the pain over the last month will be completed by each subject at visits II, III and IV (and Early Termination, if applicable).

Baseline pain scores will be the scores recorded at visit 2 (Baseline I) of Pearl III (PGL09-026) and at visit F (Baseline II) of Pearl III extension (PGL09-027), similar to the quality of life questionnaires.

### 6.3.3. Myoma and uterine size

Total volume of the three largest myoma (identified at visit 1 of PGL09-026) and uterine size will be measured using transvaginal ultrasound at visits II, III and IV. Myoma volume will be measured by measuring three dimensions of the three largest myomas. For subjects having a gap of more than 3 months between visit F of PGL09-027 and the beginning of this study, a transvaginal ultrasound will be performed at visit I.

Myoma size will not be measured if the subject has undergone uterine surgery. If it is not possible to measure the size of a myoma that was present at a previous visit, it should be clarified whether this is because of technical problems or because the myoma has disappeared or shrunk in size to a point where measurement is not possible.

There are two Baselines for myoma and uterine size: myoma and uterine size recorded at visit 1(Baseline I) of Pearl III (PGL09-026) and at visit F (Baseline II) of Pearl III extension (PGL09-027).

If possible, the ultrasound should be performed by the same assessor at each visit.

#### 6.3.4. Uterine bleeding pattern

Subjects will record their bleeding pattern using the Pictorial Bleeding Assessment Chart (PBAC) (see APPENDIX B) for the first 8 days of the first menstruation at re-start of the first treatment course (in Pearl extension 2) in the subject diary.

The baseline for PBAC is the PBAC taken at the first menstruation (day 1-8) following visit 1 of the Pearl III (PGL09-026) study.

### 6.4. SAFETY OBSERVATIONS AND MEASUREMENTS

Adverse event data will be collected continuously during the study as described in Section 8.

AE data will be obtained at scheduled study visits based on body weight, vital signs measurement, gynaecological and breast examination, biological laboratory assessments (haematology, chemistry, lipids, hormones FSH and E2), transvaginal ultrasound of ovaries, uterus, endometrium and myomas, endometrium biopsy and any other specific assessment (see Schedule of Study Assessments in APPENDIX A). In addition, subjects may report AEs spontaneously to investigator at any time and/or through questioning.

Complete appropriate data on all AEs experienced for the duration of the reporting period, as defined in section 8.5, will be reported on an ongoing basis in the AE pages of the eCRF.

#### 6.4.1. Body weight

Body weight will be measured with the subject in ordinary indoor clothing and without shoes at visit III.

#### 6.4.2. Vital Signs

Systolic and diastolic blood pressure (mmHg) and pulse rate (beats per minute (bpm)) will be measured in the supine position after resting 5 minutes at each visit.

#### 6.4.3. Gynaecological and Breast examination

Gynaecological examinations, including breast examination, will be performed at visits I (if applicable), II, III and IV (and Early Termination, if applicable).

#### 6.4.4. Transvaginal ultrasound of ovary, uterus, endometrium and myomas

If possible, the transvaginal ultrasound should be performed by the same operator at each visit. If possible it should be performed prior to the endometrium biopsy, if applicable.

A transvaginal ultrasound of the ovaries, uterus size, myoma size and endometrium thickness will be performed at visits II, III and IV. For subjects having a gap of more than 3 months between visit F of PGL09-027 and the beginning of this study, a transvaginal ultrasound will be performed at visit I. At these visits the size (length, height and depth) of the three largest myomas will be measured (see section 6.3.3.). The same three largest myomas identified at visit 1 in PGL09-026 should be followed-up.

#### 6.4.5. Laboratory Evaluation

For laboratory safety examinations, blood samples will be collected by experienced staff members at visit III (See APPENDIX A). Subjects will be asked to fast overnight before blood sampling as blood lipids will also be assessed.

See APPENDIX G for the detail of laboratory parameters tested.

Blood samples will be analysed in the central clinical laboratory. Actual laboratory reference ranges will be filed at Investigator's site. All laboratory results will be assessed for clinical significance by the Investigator or one of the co-Investigators.

### 6.5. OTHER OBSERVATIONS AND MEASUREMENTS

#### 6.5.1. Endometrial Biopsy

The sample collection of endometrial tissue will be performed by a gynaecologist at visit III for all subjects. For the collection of the endometrium tissue sample, a Pipelle de Cornier® (see APPENDIX H) is recommended. In order to allow collection of sufficient biopsy material, the biopsy should be performed in the late proliferative or secretory phase of the menstrual cycle (i.e., at least 10 days after and ideally not later than 18 days after the start of menstruation).

Subjects for whom visit I is on a different date than visit F (Follow-up visit in study PGL09-027), an endometrial biopsy is to be performed in the following three situations:

- visit E endometrial biopsy indicated the need to perform a visit F biopsy, however visit F biopsy could not be obtained
- visit F biopsy was obtained however evaluated as "not adequate"
- for all subjects having a gap of more than 3 months between visit F of PGL09-027 and the visit I of this study.

At visit II an endometrium biopsy will be performed only if endometrium thickness measured by transvaginal ultrasound is greater than 18mm or if there is any other clinical reason to perform the assessment.

If there is a finding in the biopsy taken at visit III other than “benign physiologic” endometrium, an additional biopsy will be performed at visit IV.

These biopsies will be collected, shipped, centrally processed and reviewed by three independent pathologists, using a rating scale which they helped to develop for this purpose as part of a group of pathologists experienced in these specific endometrial changes. The rating scale is the same which was used in Pearl III (PGL09-026) and in Pearl III extension (PGL09-027).

Subjects, who discontinue the study prior to the end of the study and have been exposed to at least 4 weeks of ulipristal acetate treatment, will be asked to attend an early termination visit, which includes the same assessments as visit IV, preferably 10-18 days after the beginning of menses, if applicable, so that an endometrium biopsy can be taken and sent to the 3 independent expert pathologists.

Subjects having no return of menses for 6 months during a drug free period will be asked to attend an early termination visit including an endometrium biopsy (if not pregnant).

## 6.6. CONCOMITANT MEDICATIONS AND THERAPIES

### 6.6.1. Permitted Medicines

Any medications other than those excluded by the protocol, which are considered necessary for the subjects’ welfare and/or which will not interfere with the study medication, may be given at the discretion of the Investigator.

The Investigator will record in the appropriate section of the eCRF all concomitant medications taken by the subject during the study from the date of signature of informed consent and for the duration of the reporting period as defined in section 8.5.

### 6.6.2. Prohibited Medicines

Medications listed in exclusion criteria (5.2.2) will be prohibited during the entire study.

However, when a prohibited medication or treatment is necessary for the subject’s wellbeing, the sponsor must be notified and possible alternatives are to be discussed before administration of the prohibited medication or treatment whenever possible.

### 6.6.3. Non-Drug Therapies

Current non-drug therapies for uterine myoma include the following surgical procedures: hysterectomy, myomectomy and uterine artery embolization and are prohibited during the course of the study and will lead to subject withdrawal.

## 6.7. SUBJECT COMPLETION AND WITHDRAWAL

### 6.7.1. Subject Completion

A subject will be considered to have completed the study if they complete all study procedures up to Visit IV inclusive, as described in the protocol.

### 6.7.2. Subject Withdrawal from Study

Subjects will be informed that they have the right to withdraw from the study at any time, without prejudice to their medical care, and that they are not obliged to state the reason(s). Any withdrawal must be fully documented in the End of Study page of the CRF and should be followed up by the Investigator.

The Investigator may withdraw a subject at any time if this is considered to be in the subject's best interest.

During the course of a study, the subject may be withdrawn for the following reasons:

- **Lack of efficacy:** Investigator judgment only. If subject's opinion only, check Subject Request. Explain in the eComment section of the eCRF End of Study page.
- **Adverse event:** Includes clinically significant new or worsening existing condition as judged by the investigator. Documented in the AE form.
- **Subject request:** Consent withdrawal, subject moved, schedule conflicts, etc. Specify the reason in the eComment section of the eCRF End of Study page.
- **Protocol violation:** Major protocol violation which would confound interpretation of the results. Specify the protocol violation in the eComment section of the CRF End of Study page.
- **Lost to follow-up:** Two documented phone calls and a registered letter requesting acknowledgement of receipt without response. Document in the eComment section of the CRF End of Study page.
- **Pregnancy:** **Study drug must be immediately discontinued**. Complete the Pregnancy Surveillance Form (see section 8.6 of protocol).
- **Missing return of menstruation:** Subjects having no return of menses for 6 months during a drug free period will be withdrawn.

- **Other:** Specify in the eComments section on the eCRF End of Study page. This reason should only be used if the reason for discontinuation is not better accounted for by another category.

Subjects who undergo any type of surgery or intervention which would interfere with study endpoints (i.e. hysterectomy, myomectomy, uterine artery embolization (UAE), etc.) will be discontinued from the study.

Subjects, who discontinue after at least four weeks of treatment with ulipristal acetate, will be asked to undergo the same assessments as those planned during visit IV, plus an endometrium biopsy.

Subjects, who discontinue prior to four weeks of treatment with ulipristal acetate, will be asked to attend a follow-up visit, which will include the same assessments as visit IV, without an endometrium biopsy.

Myoma size will not be measured if the subject has undergone uterine surgery.

#### 6.7.3. Subject Replacement

Subjects who discontinue the study will not be replaced.

### 6.8. PLANNED EXTENSION STUDIES

No extension studies are planned.

## 7. INVESTIGATIONAL MEDICINAL PRODUCT AND OTHER DRUGS USED IN THE STUDY

The term "Investigational Medicinal Product" (IMP) will refer to either the PregLem investigational drug undergoing study or any comparator drug, including placebo.

### 7.1. DESCRIPTION OF INVESTIGATIONAL MEDICINAL PRODUCTS

PGL4001 (ulipristal acetate) is a steroid compound (17α-Acetoxy-11β-(4-N,N-dimethylaminophenyl)-19-norpregna-4,9-diene-3,20-dione). PGL4001 10 mg is a 300 mg round, biconvex, white to off-white tablet containing 10 mg drug substance per tablet engraved with "ES10" on one side. Tablets are manufactured using direct compression process and are for oral administration. The tablets are packaged in blister pack (PVC/PE/PVDC-aluminium) and should be stored at ambient conditions and protected from light.

**Study Drug: PGL4001**

INN: ulipristal (modified INN: ulipristal acetate)

Active: PGL4001
Form: Tablet
Strength: 10mg

Weight: 300mg

Manufacturer: Catalent Germany Schorndorf GmbH, and Gedeon Richter, Budapest, Hungary

## 7.2. DOSAGE AND ADMINISTRATION

All subjects will be asked to take ulipristal acetate 10 mg tablet orally daily for 3 months (84 days) for each treatment course. There are four 3-month treatment courses in this study.

## 7.3. PACKAGING AND LABELLING

The packaging and label will be in accordance with applicable local regulatory requirements.

Ulipristal acetate 10mg tablets will be supplied by PregLem SA through Gedeon Richter (Budapest, Hungary) and Catalent Germany Schorndorf GmbH (Schorndorf, Germany) which will manufacture, pack, label, and ship them according to the current Good Manufacturing Practice (GMP) and European guidelines.

Responsibilities for manufacturing and packaging are provided in Table 1.

**Table 1: Responsibilities for Manufacturing and Packaging of PGL4001 tablets**

| Name and Address | Responsibility concerning PGL4001 tablets |
|---|---|
| Gedeon Richter Plc.<br>1103 Budapest, Gyömrői út 19-21.<br>Hungary<br>Phone: +36 1 431 5419<br>Fax +36 1 260 2851 | Manufacturing, primary packaging, quality control |
| Catalent Germany Schorndorf GmbH<br>Steinbeisstrasse 1 and 2<br>73614 Schorndorf<br>Germany<br>Phone: +49 7181 7000 428 | Labelling, secondary packaging, QP release, storage and distribution |

Quarterly study drug kits containing 12 blisters of 7 tablets each will be provided together with the corresponding release documents by the Sponsor PregLem S.A. via Catalent Germany Schorndorf GmbH.

As ulipristal acetate treatment is open-label, all subjects will receive boxes containing 12 blisters of 7 tablets of ulipristal acetate 10mg (quarterly kit).

Label examples are provided in the study file at the Investigator's site.

Each quarterly kit has one tear-off label containing the study number and the kit number. The subject will be given 2 quarterly kits for the first and second 3-month treatment courses at visit I and the other 2 quarterly kits for the third and fourth 3-month treatment courses at visit II. The tear-off labels will be taken from the boxes given to the subject and stuck on the subject log at each of these visits.

## 7.4. PREPARATION, HANDLING AND STORAGE

Ulipristal acetate tablets storage and shipment conditions are ambient and in blister pack (PVC/PE/PVDC)-aluminium. The blisters have to remain in the outer carton in order to be protected from light.

The storage facility at site should be locked and temperature-controlled.

The study drugs may be dispensed by the pharmacist or by a member of staff specifically authorised by the Investigator.

Any major deviations from the recommended ambient storage conditions should be immediately reported to the Sponsor, and the IMPs should not be used until authorisation has been given by the Sponsor.

## 7.5. INVESTIGATIONAL MEDICINAL PRODUCT ACCOUNTABILITY

The Investigator is responsible for ensuring investigational medicinal product accountability, including reconciliation of drugs and maintenance of drug records.

- Upon Receipt of IMP the Investigator (or pharmacist) will check for accurate delivery and acknowledge receipt by signing (or initialling) and dating the documentation provided by the Sponsor (or delegate) and returning it to the Sponsor (or delegate). A copy will be retained for the Investigator File.
- The dispensing of the IMP will be carefully recorded on the appropriate drug accountability forms provided by the Sponsor (or delegate) and an accurate accounting will be available for verification by the study monitor at each monitoring visit.
- IMP accountability records will include:
    - Confirmation of IMP delivery to the trial site in non-compromised conditions
    - The inventory at the site of IMP provided by the Sponsor
    - The use of each dose by each subject

- The return of full, partly used and empty blisters of IMP by each subject
- The return to the Sponsor or alternative disposition of unused IMP.
- Dates of IMP receipt, dispensation and returns, quantities, batch numbers, expiry dates and study numbers assigned to the subjects.

• The Investigator should maintain records that adequately document:
  - The subjects were provided with the doses specified by the protocol/amendment(s)
  - All IMP provided by the Sponsor were fully reconciled.

Unused IMP must not be discarded or used for any purpose other than the present study. IMP that has been dispensed to a subject must not be re-dispensed to a different subject.

The Study monitor will periodically collect the IMP accountability forms and check all investigational medicinal product dispensations and returns (both unused and used treatments) during the entire study period and prior to making arrangements for their return to the Sponsor (or delegate) in agreement with the Sponsor.

#### 7.5.1. Other drugs to be used in the study

Not applicable.

### 7.6. ASSIGNMENT TO TREATMENT GROUPS

All subjects enrolled into the study will receive open-label treatment with ulipristal acetate. Each quarterly kit will bear a unique kit number to facilitate drug accountability. The blisters contained in the quarterly kit will bear the same kit number as the quarterly kit. Kit numbers will be 5 digits in length and will start with a leading V e.g. V0001, V0002, V0003 etc. The investigator will distribute 2 quarterly kits to the subject at visit I and the other 2 quarterly kits at visit II in a random order and, if applicable, dispense kits that will expire first.

### 7.7. ASSESSMENT OF INVESTIGATIONAL MEDICINAL PRODUCT COMPLIANCE

Subjects should be instructed to bring with them to each visit both opened and unopened Investigational Medicinal Product packages, in order to allow the assessment of compliance with study treatment. Investigational Medicinal Product administration must be recorded in the CRF (or the patient diary). The Study Clinical Research Associates (CRAs) will monitor subject’s compliance during the entire study period.

### 7.8. METHOD OF BLINDING

Ulipristal acetate will be administered to all subjects in an open-label manner.

### 7.9. EMERGENCY UNBLINDING

Not applicable.

### 7.10. TREATMENT OF OVERDOSE AND MISUSE

An overdose is defined as any dose (i.e. quantity of drug given per administration or per day) above the maximum dosage defined in the protocol.

Misuse is the term used if more precise information is not available and additional information is needed to determine if there was a “medication error”, “drug abuse” or “overdose”.

Any details of overdose or misuse must be recorded in the study medication section of the eCRF.

Any case of overdose or misuse must be reported using an AE form or a SAE form, as applicable.

# 8. ADVERSE EVENTS AND SERIOUS ADVERSE EVENTS

Comprehensive assessments of any apparent toxicity experienced by the subject will be performed throughout the course of the study from the time of subject’s signature of informed consent. Study site personnel will report any adverse event, whether observed by the investigator or reported by the subject (see section 8.2.1, Eliciting Adverse Events).

The safety profile of ulipristal acetate will be assessed through the recording, reporting and analysis of baseline medical conditions, adverse events, physical examination findings including vital signs, gynaecological and breast examination, ovary and endometrium ultrasound, endometrium biopsies and laboratory tests.

The recording period for adverse events is described in section 8.5.

### 8.1. ADVERSE EVENTS

#### 8.1.1. Definitions

**Adverse Event:**

An adverse event (AE) is defined as any untoward medical occurrence in a clinical trial subject administered an investigational medicinal product (IMP) and which does not necessarily have a causal relationship with this treatment. It can therefore be any unfavourable sign (including an abnormal laboratory finding), symptom, or disease temporally associated with the use of an IMP, whether or not considered related to the IMP.

For subjects for whom Visit F (in study PGL09-027) and Visit I of the current study are on different dates, events occurring prior to visit I and outside of study PGL09-027 are not to be reported as an adverse event in this study.

Events occurring after Visit I but prior to first IMP administration in the current study will be considered as adverse events due to previous administration of the IMP during previous study participation.

**Severity:**

The severity of AEs must be assessed by investigators according to the following definitions. The term “severity” is used to describe the intensity of a specific event. This has to be distinguished from the term “serious”.

| | |
|---|---|
| Mild: | The subject is aware of the event or symptom, but the event or symptom is easily tolerated (e.g. no reduction in daily activities is required). |
| Moderate: | The subject experiences sufficient discomfort to interfere with or reduce his or her usual level of activity. |
| Severe: | Significant impairment of functioning: the subject is unable to carry out usual activities and/or the subject’s life is at risk from the event. |

**Causality assessment:**

The causality assessment of an AE to the IMP will be rated as follows by the investigator:

| | |
|---|---|
| Not related: | There is no reasonable possibility of causal relationship between an AE and IMP. |
| Related: | There is at least a reasonable possibility of a causal relationship between an AE and an IMP. This means that there are facts (evidence) or arguments to suggest a causal relationship. |

### 8.1.2. Abnormal laboratory findings and other objective measurements

Abnormal laboratory findings and other objective measurements (e.g. blood pressure, endometrium biopsy, ultrasound measurements) must be reported as an Adverse Event only if assessed by the investigator as “clinically significant” e.g. meeting at least one of the following conditions:

1. The abnormality suggests a disease and/or organ toxicity AND this abnormality was not present at the baseline (visit F in PGL09-027) or is assessed as having evolved since the baseline visit.

2. The abnormality is a Serious Adverse Event

3. The abnormality results in discontinuation of the IMP

4. The abnormality requires medical intervention or concomitant therapy

The investigator must initial and date each laboratory report/CRF page and note directly on the report/CRF page whether or not each out-of-range laboratory result is clinically significant. The outcome of this assessment will be reported using an AE or SAE form, as appropriate.

When reporting an abnormal laboratory finding in the AE page of the eCRF, a clinical diagnosis should be recorded rather than the abnormal value itself, if available (for example, “anaemia” rather than “decreased red blood cell count”)

For all of these adverse events, whether or not related to the treatment, the laboratory test(s) will be followed-up as appropriate.

#### 8.1.3. Baseline Medical Conditions

Medical conditions present at the baseline visit (visit I in the current study PGL11-024) that do not worsen in severity or frequency during the study (PGL11-024) are defined as Baseline Medical Conditions. These medical conditions should be adequately documented on the “medical history page of the eCRF”. Baseline Medical Conditions, excluding the disease under study (refer to 8.1.4), that worsen in severity or frequency during the study should be recorded and reported as adverse events.

#### 8.1.4. Exacerbation of uterine myoma

In this protocol, symptoms and signs of exacerbation or worsening of uterine myoma will usually be captured in the context of efficacy assessment, and recorded in the relevant sections of the eCRF. Therefore, symptoms, exacerbation or worsening of uterine myoma will NOT be considered as adverse events nor captured on the AE page of the eCRF unless clinically significant and not consistent with the anticipated natural progression of the disease.

Lack of efficacy of the IMP is NOT considered as an adverse event.

#### 8.1.5. Adverse Events of Special Interest

An Adverse Event of Special Interest (AESI) is an adverse event of scientific or medical concern specific to the Sponsor or the particular product or program, for which ongoing monitoring and rapid communication by the investigator to the Sponsor may be appropriate. It may require further investigation in order to characterise and understand them. It could be serious or non-serious, and could include events that might be potential precursors or prodromes for more serious medical conditions in susceptible individuals.

The AEs of special interest for this protocol have been pre-defined as follows:

- Any intervention for endometrium thickening, which is undertaken outside the protocol (surgical intervention, endometrial biopsy or medical treatment).
- Any diagnosis of endometrium biopsy other than benign endometrium done by local pathologists (biopsy performed outside the protocol).
- Acute uterine bleeding requiring immediate intervention (dilatation, curettage, other surgery or blood transfusion).
- Drug Induced Liver Injury (DILI questionnaire to be filled also).

Any AESI should be reported to Sponsor as described in section 8.3.3.

## 8.2. PROCEDURES FOR ELICITING, RECORDING AND REPORTING ADVERSE EVENTS

### 8.2.1. Eliciting Adverse Events

Data on adverse events will be obtained at scheduled or unscheduled study visits, based on information spontaneously provided by the subject and/or through questioning of the subject.

Adverse event data may also be obtained from subject diary cards, but information thus collected must be reviewed and assessed medically before it is transcribed to the eCRF.

To elicit adverse events, questioning at each study visit should begin with simple non-leading questions. For example:

- How have you felt since your last visit?
- Have you had any health problems since you were here last?

If a subject is seen by a physician not involved with the study in relation to an adverse event, the Investigator should make every effort to contact the treating physician in a timely manner in order to obtain all information necessary for the appropriate reporting of the event.

### 8.2.2. Recording of Adverse Events in the CRF

As the quality and precision of acquired AE data are critical, Investigators should use the adverse event definitions provided in the above sections and should follow this guideline when completing the AE pages of the eCRF:

- Whenever possible, recognised medical terms should be used to describe AEs rather than colloquialisms (for example, ‘influenza’ rather than ‘flu’), and abbreviations should be avoided in provided AE term.
- Adverse events should be described using a specific clinical diagnosis, if this is available, rather than a list of component signs or symptoms (for example, ‘congestive heart failure’ rather than ‘dyspnoea, rales and cyanosis.’)
- However, signs and symptoms that are not linked (as "co-manifestations”) to an identified disease or syndrome, or for which an overall diagnosis is not available, should be reported as individual AEs in separate eCRF AE page(s).
- Provisional diagnosis (e.g. “suspected Myocardial Infarction”) are acceptable but should be followed up to a definite diagnosis, if finally available.

- Adverse events occurring secondary to other events (e.g. sequelae or complications) should be identified by the primary cause. A primary AE, if clearly identifiable, generally represents the most accurate clinical term to record in the CRF. The Investigator should be invited to provide his/her opinion of which is the primary AE.

#### 8.2.3. Reporting of Adverse Events

Complete and accurate data on all AEs experienced for the duration of the reporting period, as defined in section 8.5, will be reported on an ongoing basis in the AE pages of the eCRF.

It is important that each AE report includes a description of the event, whether it is considered serious (and if so the criterion satisfied), its duration (onset and resolution dates), its severity, its relationship to the IMP(s), any other potential causality factors, any treatment given or other action taken (including dose modification or discontinuation of the IMP) and its outcome.

### 8.3. SERIOUS ADVERSE EVENTS

#### 8.3.1. Definitions

A **Serious Adverse Event** (SAE) is defined as any untoward medical occurrence or effect that at any dose:

- **results in death,**

  i.e. the AE causes or contributes to the death.

- **is life-threatening,**

  i.e. the AE places the subject at immediate risk of death; it does not refer to AE which hypothetically might have caused death if it were more severe.

- **requires inpatient hospitalization or prolongation of existing hospitalization,**

  i.e. the AE requires at least an overnight admission or prolongs a hospitalisation beyond the expected length of stay. Hospital admissions for surgery planned before study entry, for social reasons, for any elective surgery (i.e. plastic surgery) or for normal disease management (including treatment adjustment) are NOT to be considered as SAE according to this criterion (i.e. if the protocol or the standard management of the disease under study requires planned hospitalisation).

  Subjects entering this study will most likely be eligible and may decide to undergo one of the following surgical interventions: i.e. hysterectomy or myomectomy. Scheduled hospitalisations related to these procedures will therefore **NOT** be considered a criterion of seriousness.

- **results in persistent or significant disability / incapacity,**

i.e. the AE resulted in a substantial disruption of the subject's ability to conduct normal activities.

- **is a congenital anomaly / birth defect,**

  i.e. an adverse outcome in a child or foetus of a subject exposed to the Investigational Medicinal Product before conception or during pregnancy.

- **is an important medical event, i.e. is medically significant**

  Medical and scientific judgment should be exercised in deciding whether an adverse event is serious in other situation. Important medical events that may not be immediately life-threatening or result in death or hospitalisation but may jeopardise the patient or may require intervention to prevent one of the other outcomes listed in the definition above should also be considered serious. Examples of such events are intensive treatment in an emergency room, or at home for allergic bronchospasm, blood dyscrasias or convulsions that do not result in hospitalisation, or development of drug dependency or drug abuse.

### 8.3.2. SAE urgent reporting procedure

If a SAE occurs from subject consent through end of study, regardless of relationship and expectedness, the investigator is to take prompt and appropriate therapeutic action, if necessary, to protect the safety of study subjects and report such SAE as following.

The Investigator must notify **UBC Safety Europe** (acting on behalf of PregLem Drug Safety & Pharmacovigilance) **WITHIN 24 HOURS** of awareness of a new SAE or of new information on a previously reported SAE (=follow-up).

To do so, the Investigator must complete a SAE report and send it directly to UBC Safety Europe (Geneva, Switzerland) by e-mail or facsimile, using the dedicated e-mail address or facsimile numbers specified below:

**Name: UBC Safety Europe / PregLem Drug Safety and Pharmacovigilance**
**E-mail: drugsafetypharmacovigilance@preglem.com**
**Facsimile: +41 22 596 44 46**

If the Investigator, prior sending SAE report, makes a **telephone call** to **+41 22 596 44 44 UBC Safety Europe** number (notifying Sponsor immediately about SAE), this should be followed with a written SAE report within 1 **working day**.

The SAE follow-up observation period, for the concerned subjects, will be jointly decided by the Investigator or one of the co-investigator (in case of Investigator's absence) and the Sponsor.

In addition, the Investigator must respond to any request for follow-up information or questions the Sponsor may have, regarding the SAE, within 1 working day for urgent queries or 5 working days for normal queries. SAE will be followed until the Investigator and PregLem agree that the event is satisfactorily documented and resolved.

For any new SAE, the following minimum information is required as initial notification:

- Clear identification of the Investigator/Reporter with full contact information or site number
- Subject identification details (study number, site number, subject's unique study identification number and date of birth)
- Investigational Medicinal Product(s) administration details (dose and dates)
- Diagnosis of the event (or a brief description of signs/symptoms/clinical course if the diagnosis is not available) and the date of onset
- Seriousness criteria, see 8.3.1
- Causal relationship (Investigator's opinion) of the event with the Investigational Medicinal Product(s) or with the trial procedures.

### 8.3.3. Study specific reporting procedure

With any **AE of Special Interest (AESI)** occurring during this trial (see section 8.1.5), regardless of causal relationship and expectedness, the Investigator is to follow the reporting procedure as follows:

- Take prompt and appropriate medical action, if necessary, the safety of study subjects is the first priority.
- Complete the AE form in the eCRF. Complete as appropriate the AESI form or SAE form (and DILI form, if applicable), E-mail (or fax) the completed and signed form(s), accompanied by the CRF pages of demographic, medical history, previous treatments, non-drug therapy and concomitant treatments pages of the eCRF, and laboratory results (as appropriate) immediately to **UBC Safety Europe**, **(contact numbers in section 8.3.2)**, but **not later than 24 hours.**
- Monitor and record the progress of the event until it resolves or reaches a clinically stable outcome, with or without sequelae. All additional follow-up evaluations must be reported by the site to **UBC Safety Europe** as soon as possible but **not later than 24 hours after notice**. AESIs will be followed until the Investigator and Sponsor agree that the event is satisfactorily resolved.
- Obtain and maintain sufficient source documentation of pertinent medical records, relevant information, and medical judgments from colleagues who assisted in the treatment and follow-up of the subject. If necessary, contact the subject's personal physician or hospital staff to obtain further details.

## 8.4. REPORTING TO THE INDEPENDENT ETHICS COMMITTEE

The Investigator must comply with any applicable requirement related to the reporting of SAEs involving his/her subjects to the Independent Ethics Committee (IEC) that approved the study. In particular, all deaths must be promptly reported to the IEC that approved the study.

PregLem will comply with the applicable regulatory requirements related to the expedited reporting of suspected unexpected serious adverse reactions (SUSARs) to the regulatory authorities (e.g. Health Authority, Central Ethics Committee).

In accordance with ICH GCP guidelines, the Sponsor will inform the Investigator of “findings that could affect adversely the safety of subjects, impact the conduct of the trial or alter the IEC’s approval/favourable opinion to continue the trial”. In particular and in line with respective regulations, the Sponsor will inform the Investigator of adverse events that are both serious and unexpected (i.e. as per the ulipristal acetate Investigator Brochure) and are considered by Investigator or Sponsor, to have a reasonable possibility of causal relationship between the administered IMP and adverse event. The Investigator will keep copies of these safety reports in the Investigator’s file. National regulations with regards to safety reports notifications to Investigators will be taken into account.

Unless clearly defined otherwise by national or site-specific regulations, and duly documented, the responsible Investigator will promptly notify the concerned IEC of any safety reports provided by the Sponsor and provide copies of all related correspondence to the Sponsor. Only when specifically required by regulations, will the Sponsor (or delegate) provide appropriate safety reports directly to the concerned IEC and maintain records of these notifications.

## 8.5. REPORTING PERIOD

Adverse Events (AEs) are collected on an ongoing basis from the day of signed informed consent.

All new AEs and updates on all ongoing AEs or AEs with an unknown outcome, must be recorded until the final visit IV.

A last batch of queries will be sent after last study visit if remaining ongoing/unknown outcomes of reported AEs are pending. After the last batch of queries with all collected data have been fully processed, eCRFs and database will no longer be updated. Only SAEs and medically relevant ongoing/unknown outcome AEs will be followed-up until resolution or stabilisation, under UBC Safety Europe responsibility.

Any SAE the site becomes aware of following end of study and that is considered to be related to the study treatment or study participation, as assessed by the Investigator, should be recorded and reported immediately, to the Sponsor.

If a subject is documented as lost-to follow-up, ongoing/unknown outcome of AE will not be followed-up.

## 8.6. PREGNANCY AND IN UTERO DRUG EXPOSURE

Only pregnancies considered related to study treatment by the Investigator (i.e. resulting from a drug interaction with a contraceptive medication) are considered as adverse events.

### 8.6.1. Initial reporting of pregnancies:

All pregnancies occurring from the date of Informed Consent signature until end of study must be recorded by convention using the **Pregnancy Surveillance Form** – **Part I** (History and Start of Pregnancy; PSF-part I).

The Investigator must notify the Sponsor in an expedited manner (same procedure as for SAE reporting, see section 8.3.2) of any pregnancy occurring during the above-mentioned period (see section 8.5), by completing the **PSF-part I**.

If pregnancy occurs in a clinical trial subject during treatment or post-treatment period, the Investigator is to withdraw the subject from the study.

This initial form (PSF-part I) should be sent to **UBC Safety Europe** as per the same procedures and timelines described for expedited AE reporting in section 8.3.2. This form should be accompanied, as needed, by printouts of the eCRF medical history, previous and concomitant therapy and the End of Study pages.

### 8.6.2. Follow-up of pregnancies:

Investigators must actively follow-up, document and report to **UBC Safety Europe** the progress by **tri-monthly updates up to the final outcome of the pregnancy** using the **Pregnancy Surveillance Form – Part II**: (Course of Pregnancy; PSF-part II). If the subject can no longer be reached (lost to follow-up), documentation of the non-response (contact with two phone calls and a registered letter, with acknowledgement of receipt) is required.

**Pregnancy outcomes** must be reported to **UBC Safety Europe** by completing the **Pregnancy Surveillance Form – Part III**: (Outcome of Pregnancy; PSF-part III). Timelines vary according to the nature of the pregnancy outcome:

- For normal outcomes, **UBC Safety Europe** should be notified within 45 days of birth/delivery using the PSF-Part III.
- For abnormal outcomes, the fully completed PSF-Part III. must be sent to **UBC Safety Europe** according to the same procedures and timelines described for expedited AE reporting in section 8.3.2 (within 24 hours of awareness of this outcome). A SAE report form must be completed when either the mother, foetus or child sustains an event. Abnormal outcomes are defined as:
    - Abnormality of the baby (birth defect): in this case please complete in supplement to the PSF-III a SAE form for the child.

- Abnormality during the pregnancy (spontaneous abortion, stillbirth) or abnormality for the birth itself which could fulfil criteria of serious adverse events (e.g. prolongation of hospitalization due to caesarean section complications): in this case please complete in supplement to the PSF-III a SAE form for the mother.

# 9. DATA ANALYSIS AND STATISTICS

## 9.1. TEST OF HYPOTHESES

As only a descriptive analysis is planned for this open-label single treatment (ulipristal acetate 10mg) study, no formal hypothesis tests have been pre-specified in the protocol.

## 9.2. SAMPLE SIZE

No formal sample size calculation has been performed. All subjects will have previously completed Pearl III (PGL09-026) and its extension (PGL09-027). It is not possible to estimate precisely how many subjects will elect to participate in this study though it is anticipated to be about 70 to 90 subjects.

## 9.3. RANDOMISATION

Not applicable.

## 9.4. POPULATION FOR ANALYSIS

The Full Analysis Set (FAS) is defined as all subjects who are enrolled into PGL11-024 and who receive at least one dose of PGL4001. Both safety and efficacy will be evaluated on the FAS. Sub-group analyses may be conducted based on subjects who have fully completed 2 treatment cycles (attended visit II) or all 4 treatment cycles (attended visit III) of PGL11-024.

The Completer's Set is defined as all subjects who complete all four cycles of treatment in PGL11-024, who do not have surgery performed up to visit III and who do not withdraw prior to study completion (defined as attending visit IV). If applicable, two summaries of visit IV will be produced for the Completer's Population. One will include subjects that had surgery performed between visit III and visit IV, and the other will exclude those subjects in the Completer's Population that had surgery performed between visit III and visit IV.

All subjects who have had at least one visit I assessment conducted but who have not subsequently received open-label PGL4001 medication in PGL11-024 at least once will be included in the not treated population.

## 9.5. DATA ANALYSIS

Further details of the proposed statistical analysis will be documented in a statistical analysis plan (SAP) which will be written following finalisation of the protocol and finalised prior to database lock.

A descriptive analysis is planned to be performed. Descriptive statistics will be determined for all measured as well as derived endpoints. For continuous data and for ordered categorical data, if appropriate, the number of non-missing observations, mean, standard deviation, minimum, median, and maximum will be calculated. For ordered categorical data and nominal data, absolute and relative frequencies (in %) will be calculated.

Data will be summarised for all subjects treated during PGL11-024, summarised by visit where applicable. Summaries will typically include data collected at visits made in PGL09-026 and PLG09-027. In studies PGL09-026 and PGL09-027 subjects were randomised and exposed to 10 days of treatment with either norethisterone acetate or placebo immediately after each three month course of PGL4001. It is not planned to differentiate between these two treatment groups for the summaries produced in this extension study.

Raw data will be listed.

### 9.5.1. Baseline Assessment

Baseline data (i.e. data collected prior to the first administration of PGL4001 in PGL 09-026) will be summarised by descriptive statistics for the FAS and the Completer’s Set.

### 9.5.2. Primary Efficacy Analysis

The average score for the Global Study Treatment Satisfaction Questionnaire will be derived as the average scores of questions 1, 2 and 3 and will be summarised by descriptive statistics as a continuous variable at visits II and III for the FAS and the Completer’s Set.

### 9.5.3. Secondary Efficacy Analysis

All secondary efficacy endpoints will be summarised by descriptive statistics at each visit for the FAS and Completers Set. The average score for the Global Study Treatment Satisfaction Questionnaire at visits I and IV will be derived as the average scores of questions 1, 2 and 3. The profile of the Global Study Treatment Satisfaction Questionnaire (both average score and individual component scores) across visits I, II, III and IV will be examined.

### 9.5.4. Safety Analysis

All safety endpoints will be summarised by descriptive statistics at each visit for the FAS and Completers Set. Adverse events will be summarised overall and by treatment cycle.

#### 9.5.5. Exploratory Analysis

All exploratory endpoints will be summarised by descriptive statistics at each visit for the FAS and Completers Set.

#### 9.5.6. Missing Data

In general missing data will not be imputed or carried forward to the time point of interest. Data collected at early termination visits may be used as replacements of missing scheduled study visits dependent on the timing of the study withdrawal and the subsequent early termination visit.

### 9.6. STUDY SPECIFIC DATA ANALYSIS

#### 9.6.1. Interim Analysis

An interim analysis of study data may be performed at the request of regulatory authorities or to support regulatory submissions. No other interim analysis of any study data is planned although safety data will be reviewed on a regular basis.

#### 9.6.2. Final Analysis

After the last subject has completed the follow-up visit and all data have been entered into the clinical database, cleaned and locked, the results for the study will be analysed and described in a Clinical Study Report which will be submitted to the regulatory authorities.

## 10. STUDY ADMINISTRATION

### 10.1. REGULATORY AND ETHICAL CONSIDERATIONS

This study is to be performed in accordance with the protocol, the Declaration of Helsinki (see APPENDIX J), the ICH Harmonised Tripartite Guideline for GCP, and all applicable local regulatory requirements.

#### 10.1.1. Informed Consent

Before a subject can participate in the study, he or she must give written informed consent. The informed consent process will be in accordance with ICH GCP, the Declaration of Helsinki and local regulatory requirements.

Subject Information Leaflets/Informed Consent Forms will be based on a master document provided by the Sponsor, and must be approved by the Sponsor before submission to the IEC. The Sponsor must approve any changes requested by the IEC before the documents are used.

#### 10.1.2. Regulatory Authority Approval

Before the study is initiated at a site, the Sponsor (or its delegate) will obtain approval to conduct the study from the appropriate regulatory authority in accordance with any applicable country-specific regulatory requirements.

#### 10.1.3. Independent Ethics Committee Requirements

Before initiation of the study at a given centre, written approval of the protocol, Informed Consent Form and any information presented to potential subjects must be obtained from the appropriate Independent Ethics Committee. If any amendments to any of these documents occur during the study, notification or written approval as appropriate must be obtained prior to their implementation. The Investigator is responsible for ensuring that these actions occur.

Where required by local regulations, the Sponsor (or its delegate) is responsible for ensuring IEC approval of the study.

#### 10.1.4. End of the study

For administrative and safety reporting purposes the end of the study will be defined as the date of the final clinical database lock. This provides for a single and conservative definition across all study sites.

### 10.2. INVESTIGATOR RESPONSIBILITIES

The Investigator must be familiar with and conduct the study according to ICH GCP guidelines, “the EU Clinical Trial Directive” and applicable local laws and regulations.

#### 10.2.1. Coordinating Investigator

The Coordinating Investigator for this Study will be Professor Jacques Donnez, Université Catholique de Louvain, Cliniques Universitaires St Luc, 10 Avenue Hippocrate, 1200 Brussels, Belgium. The coordinating Investigator will:

- act as a representative for Investigators for decisions and discussions regarding this Study as required
- provide expert medical input and advice relating to study design and execution
- be responsible for the primary review and sign-off of the Final Report on behalf of all Investigators

Where required by local regulations, national level coordinating Investigators may also be appointed. Their responsibilities are outlined in a separate agreement with the sponsor.

### 10.3. DATA MANAGEMENT

The Investigator or designee will be responsible for entering study data in the electronic CRF provided by the Sponsor. It is the Investigator's responsibility to ensure the accuracy of the data entered in the eCRFs.

The data will be entered into a validated database. MDSL International data management will be responsible for data processing, in accordance with Sponsor (or delegate) data management procedures. Database lock will occur once quality assurance procedures have been completed. Copies of the data captured in the eCRFs will be sent to the Investigator at the completion of the study. The database will not be locked before all data clarifications have been resolved and monitored and the decision on subject evaluation has been completed.

### 10.4. STUDY MONITORING

The Investigator must ensure that the electronic CRFs are completed in a timely manner and must allow a Sponsor representative (CRA) periodic access to eCRFs, subject records and all study-related materials. The frequency of monitoring visits will be determined by factors such as the design of the study, the frequency of subject visits and the site enrolment rate. In order to verify that the study is conducted in accordance with ICH GCP, regulatory requirements, and the study protocol and that the data are authentic, accurate and complete, the study monitor will review eCRFs and other study documents and will conduct source data verification. The CRA will have the possibility to access the eCRFs remotely during the course of study.

Upon study completion, the Sponsor CRA or monitor will visit the site to conduct a Study termination visit. This will involve collection of any outstanding documentation.

### 10.5. STUDY SUPERVISORY COMMITTEE (SSC)

A study supervisory committee (SSC) will oversee the study. The SSC will be composed of both Sponsor Representatives and Sponsor independent participants. Composition, responsibilities, rules for decision and procedures for the SSC will be described in more details in a specific SSC charter.

The SSC will be responsible for safeguarding the interest of trial participants and monitoring the safety of ulipristal acetate on-off treatment during the trial on a regular basis. The SSC will review study progress and a set of safety data which will be detailed in the SSC Charter.

### 10.6. SUBJECT CONFIDENTIALITY

The Investigator and the CRA (monitor) representing the Sponsor must ensure that the subjects' anonymity is maintained. On the CRFs or other documents submitted to the Sponsor, subjects should not be identified by their names, but by their assigned identification number. If subject names are included on copies of documents submitted to the Sponsor, the names must be obliterated and the assigned subject numbers added to the documents.

The Investigator should keep a separate log of subjects' identification numbers, names, addresses, telephone numbers and hospital numbers (if applicable). Documents not for submission to the Sponsor, such as signed Informed Consent Forms, should be maintained in strict confidence by the Investigator.

### 10.7. QUALITY ASSURANCE

In compliance with ICH GCP and regulatory requirements, the Sponsor, a third party acting on behalf of the Sponsor, regulatory agencies or IECs may conduct quality assurance audits at any time during or following a study. The Investigator must agree to allow auditors direct access to all study-related documents including source documents, and must agree to allocate his or her time and the time of his or her study staff to the auditors in order to discuss findings and issues.

### 10.8. STUDY OR SITE DISCONTINUATION

The Sponsor may temporarily or permanently discontinue the study at a single site or at all sites for safety, ethical, compliance or other reasons. If this is necessary, the Sponsor will endeavour to provide advance notification to the site. If the site or study is suspended or discontinued, the Investigator will be responsible for promptly informing the IEC.

Where required by local regulations, the Sponsor (or delegate) will be responsible for informing the IEC of study or site discontinuation. In such cases, all study data and unused Investigational Medicinal Products must be returned to the Sponsor.

### 10.9. RETENTION OF ESSENTIAL STUDY DOCUMENTS

Essential documents as defined by ICH GCP include the signed protocol and any amendment(s), electronic copies of the completed CRFs on compact discs, signed Informed Consent Forms from all subjects who consented, hospital records, diary cards and other source documents, IEC approvals and all related correspondence including approved documents, drug accountability records, study correspondence and a list of the subjects' names and addresses.

The Investigator must retain copies of the essential documents for the period specified by ICH GCP and by applicable regulatory requirements.

The Investigator will inform the Sponsor of the storage location of the essential documents, and must contact the Sponsor for approval before disposing of any. The Investigator should take measures to prevent accidental or premature destruction of these documents.

# 11. REFERENCES


(1) Chabbert-Buffet, N.; Meduri, G.; Bouchard, P.; Spitz, I. M. Selective Progesterone Receptor Modulators and Progesterone Antagonists: Mechanisms of Action and Clinical Applications. *Hum. Reprod. Update.* **2005,** *11,* 293-307.

(2) Chwalisz, K.; Perez, M. C.; Demanno, D.; Winkel, C.; Schubert, G.; Elger, W. Selective Progesterone Receptor Modulator Development and Use in the Treatment of Leiomyomata and Endometriosis. *Endocr. Rev.* **2005,** *26,* 423-438.

(3) HRA pharma. Prospective Randomized Multicenter Phase II Study of the Dose-Response Effects of Continuous Administration of Low-Dose VA2914 on Parameters of the Hypothalamic-Pituitary-Gonadal Axis and the Endometrium. Final Study Report 2914-002 / hra2914-510. 2008. 2006.

(4) Levens, E. D.; Potlog-Nahari, C.; Armstrong, A. Y.; Wesley, R.; Premkumar, A.; Blithe, D. L.; Blocker, W.; Nieman, L. K. CDB-2914 for Uterine Leiomyomata Treatment: a Randomized Controlled Trial. *Obstet. Gynecol.* **2008,** *111,* 1129-1136.

(5) Donnez, J.; Tatarchuk, T. F.; Bouchard, P.; Puscasiu, L.; Zakharenko, N. F.; Ivanova, T.; Ugocsai, G.; Mara, M.; Jilla, M. P.; Bestel, E.; Terrill, P.; Osterloh, I.; Loumaye, E. Ulipristal Acetate Versus Placebo for Fibroid Treatment Before Surgery. *N. Engl. J. Med.* **2012,** *366,* 409-420.

(6) Donnez, J.; Tomaszewski, J.; Vazquez, F.; Bouchard, P.; Lemieszczuk, B.; Baro, F.; Nouri, K.; Selvaggi, L.; Sodowski, K.; Bestel, E.; Terrill, P.; Osterloh, I.; Loumaye, E. Ulipristal Acetate Versus Leuprolide Acetate for Uterine Fibroids. *N. Engl. J. Med.* **2012,** *366,* 421-432.

(7) Spies, J. B.; Coyne, K.; Guaou, G. N.; Boyle, D.; Skyrnarz-Murphy, K.; Gonzalves, S. M. The UFS-QOL, a New Disease-Specific Symptom and Health-Related Quality of Life Questionnaire for Leiomyomata. *Obstet. Gynecol.* **2002,** *99,* 290-300.

(8) Jacobson, G. F.; Shaber, R. E.; Armstrong, M. A.; Hung, Y. Y. Hysterectomy Rates for Benign Indications. *Obstet. Gynecol.* **2006,** *107,* 1278-1283.

(9) Brown, J. S.; Sawaya, G.; Thom, D. H.; Grady, D. Hysterectomy and Urinary Incontinence: a Systematic Review. *Lancet* **2000,** *356,* 535-539.

(10) Lethaby, A.; Vollenhoven, B.; Sowter, M. Pre-Operative GnRH Analogue Therapy Before Hysterectomy or Myomectomy for Uterine Fibroids. *Cochrane. Database. Syst. Rev.* **2001,** CD000547.

(11) Stovall, T. G.; Muneyyirci-Delale, O.; Summitt, R. L., Jr.; Scialli, A. R. GnRH Agonist and Iron Versus Placebo and Iron in the Anemic Patient Before Surgery for Leiomyomas: a Randomized Controlled Trial. Leuprolide Acetate Study Group. *Obstet. Gynecol.* **1995,** *86,* 65-71.

(12) Horne, F. M.; Blithe, D. L. Progesterone Receptor Modulators and the Endometrium: Changes and Consequences. *Hum. Reprod. Update.* **2007,** *13,* 567-580.

(13) Mutter, G. L.; Bergeron, C.; Deligdisch, L.; Ferenczy, A.; Glant, M.; Merino, M.; Williams, A. R.; Blithe, D. L. The Spectrum of Endometrial Pathology Induced by Progesterone Receptor Modulators. *Mod. Pathol.* **2008**.

(14) Janssen, C. A. A Simple Visual Assessment Technique to Discriminate Between Menorrhagia and Normal Menstrual Blood Loss. *Eur. J. Obstet. Gynecol. Reprod. Biol.* **1996,** *70,* 21-22.

# 12. APPENDICES

| | |
|---|---|
| APPENDIX A | SCHEDULE OF STUDY ASSESSMENTS |
| APPENDIX B | PICTORIAL BLEEDING ASSESSMENT CHART (PBAC) |
| APPENDIX C | GLOBAL STUDY TREATMENT SATISFACTION QUESTIONNAIRE |
| APPENDIX D | UFS QUALITY OF LIFE QUESTIONNAIRE |
| APPENDIX E | EUROQOL EQ-5D QUESTIONNAIRE |
| APPENDIX F | VAS PAIN SCALE |
| APPENDIX G | LABORATORY PARAMETERS |
| APPENDIX H | PACKAGE INSERT OF PIPELLE DE CORNIER |
| APPENDIX I | LIST OF CYP 3A4 INDUCERS AND STRONG INHIBITORS |
| APPENDIX J | WORLD MEDICAL ASSOCIATION DECLARATION OF HELSINKI |

# APPENDIX A SCHEDULE OF STUDY ASSESSMENTS

| | Visit I<br>Same day as follow-up Visit F of Pearl III extension and 10-18 days after start of menses (exception is possible for patients who have already finished PGL09-027 prior to a possible start of PGL11-024) | Visit II<br>10-18 days after start of menses following the 2nd 3-month treatment course | Visit III<br>10-18 days after start of menses following the 4th 3-month treatment course | Visit IV<br>Follow-up visit<br>3 months after last dose of ulipristal acetate and 10-18 days after start of menses | Early termination visit |
|---|---|---|---|---|---|
| **CLINICAL ASSESSMENTS** | | | | | |
| Signed Informed Consent | X | | | | |
| Inclusion/Exclusion Criteria | X | | | | |
| Medical History | X | | | | |
| Body weight | | | X | | |
| Vital signs | X | X | X | X | X |
| Gynaecological Examination (incl. Breast examination) | X[1] | X | X | X | X |
| Urine pregnancy test | X | X | X | X | X |
| Ulipristal acetate dispensing | X | X | | | |
| Study drug compliance check | | X | X | | X |
| Subject diary dispensed | X | X | | | |
| Subject diary collected | | X | X | | |
| Dispense towels and tampons for PBAC | X | | | | |
| Specific Patient satisfaction questionnaire | X | X | X | X | X |
| UFS-QoL and EQ-5D questionnaires | | X | X | X | X |
| Pain (VAS) | | X | X | X | X |

| | Visit I<br>Same day as follow-up Visit F of Pearl III extension and 10-18 days after start of menses (exception is possible for patients who have already finished PGL09-027 prior to a possible start of PGL11-024) | Visit II<br>10-18 days after start of menses following the 2nd 3-month treatment course | Visit III<br>10-18 days after start of menses following the 4th 3-month treatment course | Visit IV<br>Follow-up visit<br>3 months after last dose of ulipristal acetate and 10-18 days after start of menses | Early termination visit |
|---|---|---|---|---|---|
| **CLINICAL ASSESSMENTS** | | | | | |
| Transvaginal ultrasound : myoma size, uterus size, ovary, endometrium thickness | X[1] | X | X | X | X[4] |
| Endometrium Biopsy | X[1,*] | X[2] | X | X[3] | X[4] |
| PAP smear | | | | X[5] | X[5] |
| Concomitant medication | X | X | X | X | X |
| Adverse event monitoring | | X | X | X | X |
| End of study page | | | | X | X |
| **CENTRAL LABORATORY** | | | | | |
| Hematology, Chemistry, Lipids (after overnight fasting) | | | X | | |
| Hormones E2 and FSH | | | | | X[6] |

[1] For subjects having a gap of more than 3 months between visit F of PGL09-027 and the beginning of this study

[*] For subjects for whom visit I is on a different date than visit F (but with a gap of less than 3 months) and for whom visit F biopsy could not be obtained or was evaluated as “not adequate”

[2] If endometrium thickness >18mm or if there are any other clinical reasons to do so

[3] If abnormal finding at visit III

[4] If applicable

[5] If no PAP smear performed in the past 12 months

[6] If menstruation has not returned for 6 months during a drug free period

# APPENDIX B PICTORIAL BLEEDING ASSESSMENT CHART

PBAC is one of the current standard methods used to objectively estimate menstrual blood loss and diagnose menorrhagia. The method which was developed and validated by Higham and Janssen[14] defines excessive bleeding as a PBAC score >100.

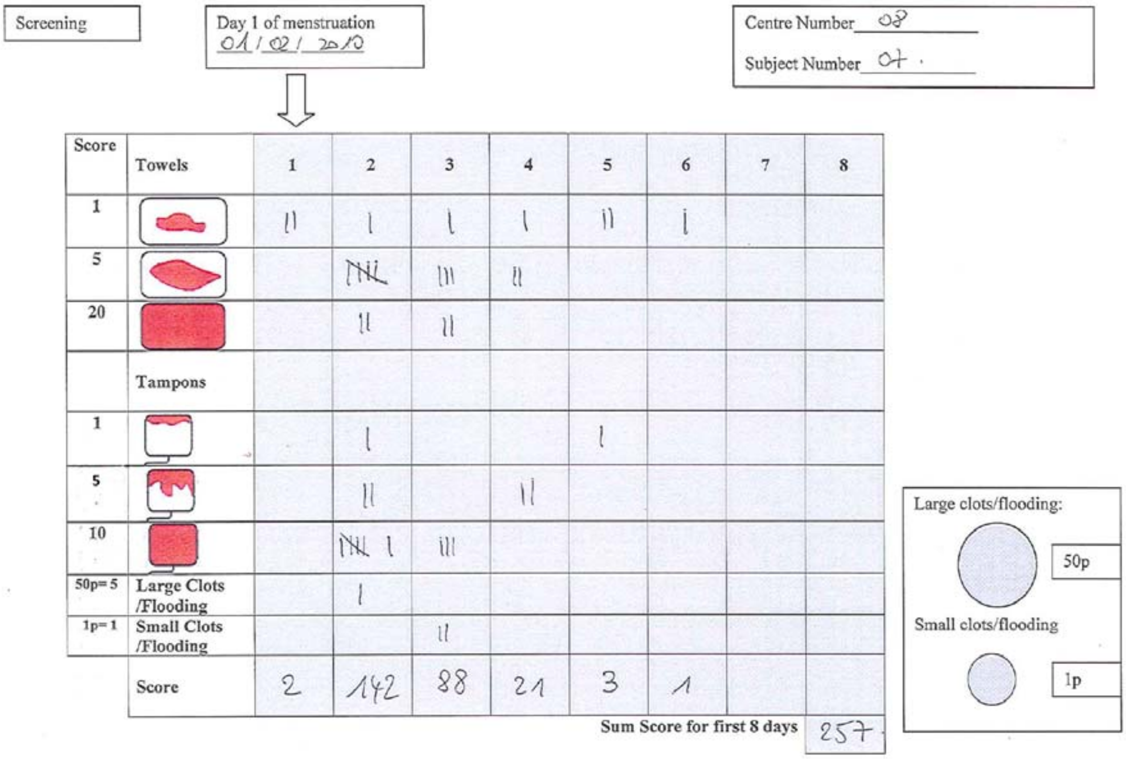

Screening

Day 1 of menstruation 01/02/2010

Centre Number 08

Subject Number 07.

| Score | Towels | 1 | 2 | 3 | 4 | 5 | 6 | 7 | 8 |
|---|---|---|---|---|---|---|---|---|---|
| 1 | | II | I | I | I | II | I | | |
| 5 | | | 卌 | III | II | | | | |
| 20 | | | II | II | | | | | |
| | Tampons | | | | | | | | |
| 1 | | | I | | | I | | | |
| 5 | | | II | | II | | | | |
| 10 | | | 卌 I | III | | | | | |
| 50p= 5 | Large Clots /Flooding | | I | | | | | | |
| 1p= 1 | Small Clots /Flooding | | | II | | | | | |
| | Score | 2 | 142 | 88 | 21 | 3 | 1 | | |
| | | | | | | | | Sum Score for first 8 days | 257 |

Large clots/flooding: 50p

Small clots/flooding: 1p

# APPENDIX C GLOBAL STUDY TREATMENT SATISFACTION QUESTIONNAIRE

**Subject Number:_______________ Centre Number: _________________________**
**Date Questionnaire completed: ____________**
**Please tick the appropriate visit:**

☐ Visit I (start of the study)
☐ Visit II (after two 3-month treatment courses)
☐ Visit III (after four 3-month treatment courses)
☐ Visit IV (follow-up visit)

**In the context of ulipristal acetate treatment, please complete this questionnaire. Questions should be answered in the order of appearance. Only one answer per question is allowed (tick only one box).**

**1. How satisfied or dissatisfied are you with the ability of the study drug to prevent or treat your fibroid symptoms?**

☐ 1 Extremely Satisfied
☐ 2 Very Satisfied
☐ 3 Satisfied
☐ 4 Somewhat Satisfied
☐ 5 Dissatisfied
☐ 6 Very Dissatisfied
☐ 7 Extremely Dissatisfied

**2. How satisfied or dissatisfied are you with the way the study drug relieves the uterine bleeding due to your fibroids symptoms?**

☐ 1 Extremely Satisfied
☐ 2 Very Satisfied
☐ 3 Satisfied
☐ 4 Somewhat Satisfied
☐ 5 Dissatisfied
☐ 6 Very Dissatisfied
☐ 7 Extremely Dissatisfied

**3. Taking all things into account, how satisfied or dissatisfied are you with this study drug?**

☐ 1 Extremely Satisfied
☐ 2 Very Satisfied
☐ 3 Satisfied
☐ 4 Somewhat Satisfied
☐ 5 Dissatisfied
☐ 6 Very Dissatisfied
☐ 7 Extremely Dissatisfied

**4. How do you estimate your menstrual bleeding now compared to before the very first intake of this study drug?**

☐ 1 Much less heavy
☐ 2 A little less heavy
☐ 3 About the same
☐ 4 A little heavier
☐ 5 Much heavier

# APPENDIX D UFS QUALITY OF LIFE QUESTIONNAIRE

The UFS QOL was developed and validated for assessing symptom severity and impact on health related quality of life for women with myoma. The questionnaire consists of eight symptoms questions and 29 health-related quality of life questions with six subscales. The UFS QOL questionnaire subscales discriminate not only from normal controls but also among myoma patients with varying degrees of symptom severity.

**UTERINE FIBROID SYMPTOM AND HEALTH-RELATED QUALITY OF LIFE QUESTIONNAIRE (UFS-QOL)**

**Listed below are symptoms experienced by women who have uterine fibroids. Please consider each symptom as it relates to your uterine fibroids or menstrual cycle. Each question asks how much distress you have experienced from each symptom during the previous 3 months.**

**There are no right or wrong answers. Please be sure to answer every question by checking (✓) the most appropriate box. If a question does not apply to you, please mark "not at all" as a response.**

| During the previous 3 months, how distressed were you by… | Not at all | A little bit | Some-what | A great deal | A very great deal |
|---|---|---|---|---|---|
| 1. Heavy bleeding during your menstrual period | ☐ | ☐ | ☐ | ☐ | ☐ |
| 2. Passing blood clots during your menstrual period | ☐ | ☐ | ☐ | ☐ | ☐ |
| 3. Fluctuation in the duration of your menstrual period compared to your previous cycle | ☐ | ☐ | ☐ | ☐ | ☐ |
| 4. Fluctuation in the length of your monthly cycle compared to your previous cycles | ☐ | ☐ | ☐ | ☐ | ☐ |
| 5. Feeling tightness or pressure in your pelvic area | ☐ | ☐ | ☐ | ☐ | ☐ |
| 6. Frequent urination during the daytime hours | ☐ | ☐ | ☐ | ☐ | ☐ |
| 7. Frequent nighttime urination | ☐ | ☐ | ☐ | ☐ | ☐ |
| 8. Feeling fatigued | ☐ | ☐ | ☐ | ☐ | ☐ |

**The following questions ask about your feelings and experiences regarding the impact of uterine fibroid symptoms on your life. Please consider each question as it relates to your experiences with uterine fibroids during the previous 3 months.**

**There are no right or wrong answers. Please be sure to answer every question by checking (✓) the most appropriate box. If the question does not apply to you, please check "none of the time" as your option.**

| During the previous 3 months, how often have your symptoms related to uterine fibroids... | None of the time | A little of the time | Some of the time | Most of the time | All of the time |
|---|---|---|---|---|---|
| 9. Made you feel anxious about the unpredictable onset or duration of your periods? | ☐ | ☐ | ☐ | ☐ | ☐ |
| 10. Made you anxious about traveling? | ☐ | ☐ | ☐ | ☐ | ☐ |
| 11. Interfered with your physical activities? | ☐ | ☐ | ☐ | ☐ | ☐ |
| 12. Caused you to feel tired or worn out? | ☐ | ☐ | ☐ | ☐ | ☐ |
| 13. Made you decrease the amount of time you spent on exercise or other physical activities? | ☐ | ☐ | ☐ | ☐ | ☐ |
| 14. Made you feel as if you are not in control of your life? | ☐ | ☐ | ☐ | ☐ | ☐ |
| 15. Made you concerned about soiling underclothes? | ☐ | ☐ | ☐ | ☐ | ☐ |
| 16. Made you feel less productive? | ☐ | ☐ | ☐ | ☐ | ☐ |
| 17. Caused you to feel drowsy or sleepy during the day? | ☐ | ☐ | ☐ | ☐ | ☐ |
| 18. Made you feel self-conscious of weight gain? | ☐ | ☐ | ☐ | ☐ | ☐ |
| 19. Made you feel that it was difficult to carry out your usual activities? | ☐ | ☐ | ☐ | ☐ | ☐ |
| 20. Interfered with your social activities? | ☐ | ☐ | ☐ | ☐ | ☐ |
| 21. Made you feel conscious about the size and appearance of your stomach? | ☐ | ☐ | ☐ | ☐ | ☐ |
| 22. Made you concerned about soiling bed linen? | ☐ | ☐ | ☐ | ☐ | ☐ |

| During the previous 3 months, how often have your symptoms related to uterine fibroids... | None of the time | A little of the time | Some of the time | Most of the time | All of the time |
|---|---|---|---|---|---|
| 23. Made you feel sad, discouraged, or hopeless? | ☐ 1 | ☐ 2 | ☐ 3 | ☐ 4 | ☐ 5 |
| 24. Made you feel down hearted and blue? | ☐ 1 | ☐ 2 | ☐ 3 | ☐ 4 | ☐ 5 |
| 25. Made you feel wiped out? | ☐ 1 | ☐ 2 | ☐ 3 | ☐ 4 | ☐ 5 |
| 26. Caused you to be concerned or worried about your health? | ☐ 1 | ☐ 2 | ☐ 3 | ☐ 4 | ☐ 5 |
| 27. Caused you to plan activities more carefully? | ☐ 1 | ☐ 2 | ☐ 3 | ☐ 4 | ☐ 5 |
| 28. Made you feel inconvenienced about always carrying extra pads, tampons, and clothing to avoid accidents? | ☐ 1 | ☐ 2 | ☐ 3 | ☐ 4 | ☐ 5 |
| 29. Caused you embarrassment? | ☐ 1 | ☐ 2 | ☐ 3 | ☐ 4 | ☐ 5 |
| 30. Made you feel uncertain about your future? | ☐ 1 | ☐ 2 | ☐ 3 | ☐ 4 | ☐ 5 |
| 31. Made you feel irritable? | ☐ 1 | ☐ 2 | ☐ 3 | ☐ 4 | ☐ 5 |
| 32. Made you concerned about soiling outer clothes? | ☐ 1 | ☐ 2 | ☐ 3 | ☐ 4 | ☐ 5 |
| 33. Affected the size of clothing you wear during your periods? | ☐ 1 | ☐ 2 | ☐ 3 | ☐ 4 | ☐ 5 |
| 34. Made you feel that you are not in control of your health? | ☐ 1 | ☐ 2 | ☐ 3 | ☐ 4 | ☐ 5 |
| 35. Made you feel weak as if energy was drained from your body? | ☐ 1 | ☐ 2 | ☐ 3 | ☐ 4 | ☐ 5 |
| 36. Diminished your sexual desire? | ☐ 1 | ☐ 2 | ☐ 3 | ☐ 4 | ☐ 5 |
| 37. Caused you to avoid sexual relations? | ☐ 1 | ☐ 2 | ☐ 3 | ☐ 4 | ☐ 5 |

# APPENDIX E EUROQUOL EQ-5D QUALITY OF LIFE QUESTIONNAIRE

**Health Questionnaire**

*English version for the UK*
*(validated for Ireland)*

SAMPLE

*© 1990 EuroQol Group EQ-5D™ is a trade mark of the EuroQol Group*

By placing a tick in one box in each group below, please indicate which statements best describe your own health state today.

**Mobility**

I have no problems in walking about ❑

I have some problems in walking about ❑

I am confined to bed ❑

**Self-Care**

I have no problems with self-care ❑

I have some problems washing or dressing myself ❑

I am unable to wash or dress myself ❑

**Usual Activities** *(e.g. work, study, housework, family or leisure activities)*

I have no problems with performing my usual activities ❑

I have some problems with performing my usual activities ❑

I am unable to perform my usual activities ❑

**Pain/Discomfort**

I have no pain or discomfort ❑

I have moderate pain or discomfort ❑

I have extreme pain or discomfort ❑

**Anxiety/Depression**

I am not anxious or depressed ❑

I am moderately anxious or depressed ❑

I am extremely anxious or depressed ❑

SAMPLE



*© 1990 EuroQol GroupEQ-5D™ is a trade mark of the EuroQol Group*

To help people say how good or bad a health state is, we have drawn a scale (rather like a thermometer) on which the best state you can imagine is marked 100 and the worst state you can imagine is marked 0.

We would like you to indicate on this scale how good or bad your own health is today, in your opinion. Please do this by drawing a line from the box below to whichever point on the scale indicates how good or bad your health state is today.

**Your own health state today**

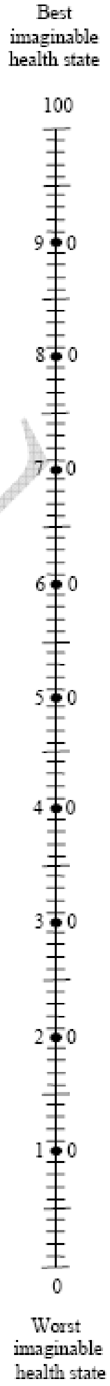


SAMPLE


*© 1990 EuroQol GroupEQ-5D™ is a trade mark of the EuroQol Group*

## APPENDIX F VAS PAIN SCALE

**Please place a mark on the line at the point indicating the severity of your pain over the last month.**

**NO PAIN** **WORST POSSIBLE PAIN**

# APPENDIX G LABORATORY PARAMETERS

<table>
<tr>
<td>Routine Haematology<br>Hemoglobin<br>Hematocrit<br>Red cell count<br>White cell count<br>Neutrophils<br>Lymphocytes<br>Monocytes<br>Eosinophils<br>Basophils<br>Platelets<br>Ferritine</td>
<td rowspan="2">Blood Chemistry<br>Sodium<br>Potassium<br>Calcium<br>Urea<br>Creatinine<br>Bilirubin total<br>Indirect Bilirubin<br>Total protein<br>Albumin<br>AST (SGOT)<br>ALT (SGPT)<br>Alkaline phosphatase<br>GGT<br>Creatine kinase<br>Lactate dehydrogenase (LDH)</td>
<td>Lipids<br>Total cholesterol<br>HDL cholesterol<br>LDL cholesterol<br>Triglycerides</td>
</tr>
<tr>
<td>Urine pregnancy test (dipstick)</td>
<td>Hormones<br>FSH<br>E2</td>
</tr>
</table>

All the above listed tests are to be performed at the frequencies indicated in Appendix A.

All above listed tests will be performed by a central laboratory (except for the urinary pregnancy test).

Please consult the central laboratory instructions manual for the preparation and handling of the blood samples to be drawn to perform these tests. This manual also explains the methodology used for measuring of each biological parameter listed above.

Blood samples for central analysis of bioanalytical measurements will be collected at the time points indicated in Appendix A. Subjects will be instructed that they must be fasting preceding blood sampling time point, if lipids are to be measured.

# APPENDIX H PACKAGE INSERT OF PIPELLE DE CORNIER®

## Pipelle de Cornier®

### I - DESCRIPTION

***the Pipelle de Cornier® includes:***

- A flexible, transparent polypropylene sheath, 3.10 mm in external diameter, 2.6 mm in internal diameter and 23.5 cm long, with a lateral orifice 2.1 mm in diameter in its distal portion and four markings 4, 7, 8 and 10 cm from this extremity. Its proximal end is indented to stop the plunger.
- An internal EVA plunger, which slides up and down when pushed by a flexible acetal resin shaft.

- Single use.
- Latex free.
- Individual packaging.
- Sterilized with ethylene oxide.

### II - INDICATIONS

The Pipelle de Cornier® generally requires no local anesthesia or cervical dilatation. As the sampling procedure is painless, the Pipelle de Cornier® can be used for systematic screening programs in women at risk.

***The Pipelle de Cornier® is indicated for the following procedures:***

- Systematic screening for endometrial cancer and hyperplasia
- Detection of luteal phase insufficiency
- Monitoring endometrial effects of hormone treatments
- Menometrorrhagia with or without HRT
- Screening in premopausal or postmenopausal women
- Investigate endometrial hypertrophy detected by ultrasonography
- Investigate polyps
- Monitor Tamoxifen treatment
- Bacteriological culture to identify pathogens.

### III - CONTRAINDICATIONS

***Suspected pregnancy:***

Evidence of an on-going pregnancy provided by an ultrasound examination or serum hCG levels is an absolute contraindication to using the Pipelle de Cornier®. As a precautionary measure, it is therefore advisable to rule out a pregnancy in women with childbearing potential and not using an effective method of contraception, by performing a serum hCG assay and an ultrasound examination less than 15 days prior to the endometrial biopsy.

***Suspected infection of the upper genital tract:***

In patients with an infection of the upper genital tract, the Pipelle de Cornier® may be used to sample endometrial tissue, to diagnose a secondary infection of neoplastic tissue, or just to collect pus for bacteriological culture. In this case, special care is warranted to avoid, even more than usual, any risk of perforation of the uterus. Ultrasound examination before or during the procedure is strongly recommended. No force must be applied if unusual resistance is met when introducing the Pipelle de Cornier®.

***Cervical stenosis:***

In many women on HRT the cervix is stenosed. In women bleeding on SERM therapy or with abnormal or atrophic endometrium, local anesthesia using a small dilator (up to CH 4) can be helpful. In most cases, the sampling procedure will cause no discomfort provided it is performed gently and slowly to allow enough time for adequate dilation.

***Very large uterus:***

If the uterus is very large (over 15 cm by hysterometry, or corpus uteri length over 10 cm), the screening procedure is less reliable. In this case, ultrasonographic examination of the uterus to determine its position, shape, and size prior to the attempt is recommended, when available.

### IV - INSTRUCTIONS FOR USE

- The Pipelle de Cornier® can be shaped before taking it out of its sterile packaging. The resilience of the material helps the device retain a given convexity to fit a uterine anteflexion or retroflexion.
- Disinfect the cervix thoroughly.
- In most cases, Pozzi forceps are not necessary. In post-menopausal women, a local anesthesia with xylocaine

## Pipelle de Cornier®

helps to clear a stenosed cervix.

- Slide the Pipelle de Cornier® gently through the cervix up to the uterine fundus. The 4 guide-mark indicates the beginning of the uterine cavity. The 7 guide-mark will generally indicate that the fundus has been reached.
- Draw back the piston to the end of the biopsy cannula until it self locks to create a negative pressure.
- Sweep the uterine fundus slowly several times up to the internal orifice of the cervix, using regular to-and-fro movements while rotating the sampler to include the whole uterine cavity in the specimen.
- Continue until fragments of uterine mucosa appear within the sheath, which generally takes 30 seconds. If the Pipelle de Cornier® "slips" before the end of the procedure, it means the sheath is full. In this case, a second Pipelle de Cornier® must be used to explore the rest of the cavity.
- Remove the Pipelle de Cornier® fully.
- To recover the histology specimen, push the plunger to release the whole content in a vial containing the fixative solution.

**V - REFERENCE**

**REF 1.103.000 PIPELLE DE CORNIER® box of 25 units**

Manufactured by **PRODIMED**
60530 – Neuilly-en-Thelle – FRANCE

Imported by CCD International
88 Eliot Street,
NATICK, MA 01760

Distributed by SEPAL Reproductive Devices, Inc.
201 South Street, 6th Floor Boston MA 02111

www.ccd-international.com

C.C.D. International
*Expertise in women's health*

## Pipelle de Cornier®

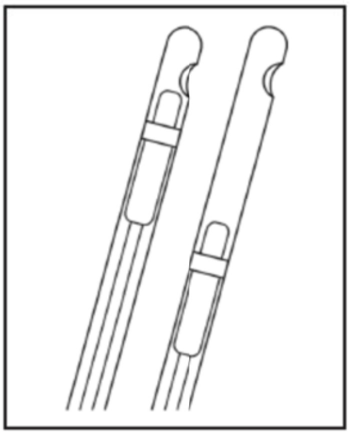

5°C 50°C

LATEX

STERILE EO

C.C.D. International
*Expertise in women's health*

Made in France
X2998 Ed.: Mars 2007

# APPENDIX I LIST OF CYP 3A4 INDUCERS AND STRONG INHIBITORS

| Inducers | Strong Inhibitors |
|---|---|
| carbamazepine | clarithromycin |
| phenobarbital | indinavir |
| phenytoin | itraconazole |
| rifampin | ketoconazole |
| pioglitazone | nefazodone |
| rifabutin | nelfinavir |
| St. John's wort | ritonavir |
| troglitazone | |

http://medicine.iupui.edu/clinpharm/ddis/clinicalTable.aspx
(Last updated on March 06, 2012) Link accessed on April 11, 2012.

# APPENDIX J WORLD MEDICAL ASSOCIATION DECLARATION OF HELSINKI

## WORLD MEDICAL ASSOCIATION (WMA) DECLARATION OF HELSINKI
## Ethical Principles for Research Involving Human Subjects

Adopted by the 18th WMA General Assembly, Helsinki, Finland, June 1964 and amended by the:
29th WMA General Assembly, Tokyo, Japan, October 1975
35th WMA General Assembly, Venice, Italy, October 1983
41st WMA General Assembly, Hong Kong, September 1989
48th WMA General Assembly, Somerset West, Republic of South Africa, October 1996
52nd WMA General Assembly, Edinburgh, Scotland, October 2000
53th WMA General Assembly, Washington, 2002 (note of clarification on Paragraph 29 added)
55th WMA General Assembly, Tokyo, 2004 (note of clarification on Paragraph 30 added)
59th WMA General Assembly, Seoul, October 2008

### A. INTRODUCTION

1. The World Medical Association (WMA) has developed the Declaration of Helsinki as a statement of ethical principles for medical research involving human subjects, including research on identifiable human material and data.

   The Declaration is intended to be read as a whole and each of its constituent paragraphs should not be applied without consideration of all other relevant paragraphs.
2. Although the Declaration is addressed primarily to physicians, the WMA encourages other participants in medical research involving human subjects to adopt these principles.
3. It is the duty of the physician to promote and safeguard the health of subjects, including those who are involved in medical research. The physician's knowledge and conscience are dedicated to the fulfilment of this duty.
4. The Declaration of Geneva of the WMA binds the physician with the words, “The health of my patient will be my first consideration,” and the International Code of Medical Ethics declares that, “A physician shall act in the patient's best interest when providing medical care.”
5. Medical progress is based on research that ultimately must include studies involving human subjects. Populations that are underrepresented in medical research should be provided appropriate access to participation in research.
6. In medical research involving human subjects, the well-being of the individual research subject must take precedence over all other interests.
7. The primary purpose of medical research involving human subjects is to understand the causes, development and effects of diseases and improve preventive, diagnostic and therapeutic interventions (methods, procedures and treatments). Even the best current interventions must be evaluated continually through research for their safety, effectiveness, efficiency, accessibility and quality.

8. In medical practice and in medical research, most interventions involve risks and burdens.
9. Medical research is subject to ethical standards that promote respect for all human subjects and protect their health and rights. Some research populations are particularly vulnerable and need special protection. These include those who cannot give or refuse consent for themselves and those who may be vulnerable to coercion or undue influence.
10. Physicians should consider the ethical, legal and regulatory norms and standards for research involving human subjects in their own countries as well as applicable international norms and standards. No national or international ethical, legal or regulatory requirement should reduce or eliminate any of the protections for research subjects set forth in this Declaration.

## B. PRINCIPLES FOR ALL MEDICAL RESEARCH

11. It is the duty of physicians who participate in medical research to protect the life, health, dignity, integrity, right to self-determination, privacy, and confidentiality of personal information of research subjects.
12. Medical research involving human subjects must conform to generally accepted scientific principles, be based on a thorough knowledge of the scientific literature, other relevant sources of information, and adequate laboratory and, as appropriate, animal experimentation. The welfare of animals used for research must be respected.
13. Appropriate caution must be exercised in the conduct of medical research that may harm the environment.
14. The design and performance of each research study involving human subjects must be clearly described in a research protocol. The protocol should contain a statement of the ethical considerations involved and should indicate how the principles in this Declaration have been addressed. The protocol should include information regarding funding, sponsors, institutional affiliations, other potential conflicts of interest, incentives for subjects and provisions for treating and/or compensating subjects who are harmed as a consequence of participation in the research study. The protocol should describe arrangements for post-study access by study subjects to interventions identified as beneficial in the study or access to other appropriate care or benefits.
15. The research protocol must be submitted for consideration, comment, guidance and approval to a research ethics committee before the study begins. This committee must be independent of the researcher, the sponsor and any other undue influence. It must take into consideration the laws and regulations of the country or countries in which the research is to be performed as well as applicable international norms and standards but these must not be allowed to reduce or eliminate any of the protections for research subjects set forth in this Declaration. The committee must have the right to monitor ongoing studies. The researcher must provide monitoring information to the committee, especially information about any serious adverse events. No change to the protocol may be made without consideration and approval by the committee.
16. Medical research involving human subjects must be conducted only by individuals with the appropriate scientific training and qualifications. Research on patients or healthy volunteers requires the supervision of a competent and appropriately qualified physician or other health care professional. The responsibility for the protection of research subjects must always rest with the

physician or other health care professional and never the research subjects, even though they have given consent.

17. Medical research involving a disadvantaged or vulnerable population or community is only justified if the research is responsive to the health needs and priorities of this population or community and if there is a reasonable likelihood that this population or community stands to benefit from the results of the research.
18. Every medical research study involving human subjects must be preceded by careful assessment of predictable risks and burdens to the individuals and communities involved in the research in comparison with foreseeable benefits to them and to other individuals or communities affected by the condition under investigation.
19. Every clinical trial must be registered in a publicly accessible database before recruitment of the first subject.
20. Physicians may not participate in a research study involving human subjects unless they are confident that the risks involved have been adequately assessed and can be satisfactorily managed. Physicians must immediately stop a study when the risks are found to outweigh the potential benefits or when there is conclusive proof of positive and beneficial results.
21. Medical research involving human subjects may only be conducted if the importance of the objective outweighs the inherent risks and burdens to the research subjects.
22. Participation by competent individuals as subjects in medical research must be voluntary. Although it may be appropriate to consult family members or community leaders, no competent individual may be enrolled in a research study unless he or she freely agrees.
23. Every precaution must be taken to protect the privacy of research subjects and the confidentiality of their personal information and to minimize the impact of the study on their physical, mental and social integrity.
24. In medical research involving competent human subjects, each potential subject must be adequately informed of the aims, methods, sources of funding, any possible conflicts of interest, institutional affiliations of the researcher, the anticipated benefits and potential risks of the study and the discomfort it may entail, and any other relevant aspects of the study. The potential subject must be informed of the right to refuse to participate in the study or to withdraw consent to participate at any time without reprisal. Special attention should be given to the specific information needs of individual potential subjects as well as to the methods used to deliver the information. After ensuring that the potential subject has understood the information, the physician or another appropriately qualified individual must then seek the potential subject's freely-given informed consent, preferably in writing. If the consent cannot be expressed in writing, the non-written consent must be formally documented and witnessed.
25. For medical research using identifiable human material or data, physicians must normally seek consent for the collection, analysis, storage and/or reuse. There may be situations where consent would be impossible or impractical to obtain for such research or would pose a threat to the validity of the research. In such situations the research may be done only after consideration and approval of a research ethics committee.
26. When seeking informed consent for participation in a research study the physician should be particularly cautious if the potential subject is in a dependent relationship with the physician or

may consent under duress. In such situations the informed consent should be sought by an appropriately qualified individual who is completely independent of this relationship.

27. For a potential research subject who is incompetent, the physician must seek informed consent from the legally authorized representative. These individuals must not be included in a research study that has no likelihood of benefit for them unless it is intended to promote the health of the population represented by the potential subject, the research cannot instead be performed with competent persons, and the research entails only minimal risk and minimal burden.
28. When a potential research subject who is deemed incompetent is able to give assent to decisions about participation in research, the physician must seek that assent in addition to the consent of the legally authorized representative. The potential subject's dissent should be respected.
29. Research involving subjects who are physically or mentally incapable of giving consent, for example, unconscious patients, may be done only if the physical or mental condition that prevents giving informed consent is a necessary characteristic of the research population. In such circumstances the physician should seek informed consent from the legally authorized representative. If no such representative is available and if the research cannot be delayed, the study may proceed without informed consent provided that the specific reasons for involving subjects with a condition that renders them unable to give informed consent have been stated in the research protocol and the study has been approved by a research ethics committee. Consent to remain in the research should be obtained as soon as possible from the subject or a legally authorized representative.
30. Authors, editors and publishers all have ethical obligations with regard to the publication of the results of research. Authors have a duty to make publicly available the results of their research on human subjects and are accountable for the completeness and accuracy of their reports. They should adhere to accepted guidelines for ethical reporting. Negative and inconclusive as well as positive results should be published or otherwise made publicly available. Sources of funding, institutional affiliations and conflicts of interest should be declared in the publication. Reports of research not in accordance with the principles of this Declaration should not be accepted for publication.

## C. ADDITIONAL PRINCIPLES FOR MEDICAL RESEARCH COMBINED WITH MEDICAL CARE

31. The physician may combine medical research with medical care only to the extent that the research is justified by its potential preventive, diagnostic or therapeutic value and if the physician has good reason to believe that participation in the research study will not adversely affect the health of the patients who serve as research subjects.
32. The benefits, risks, burdens and effectiveness of a new intervention must be tested against those of the best current proven intervention, except in the following circumstances:
    - The use of placebo, or no treatment, is acceptable in studies where no current proven intervention exists; or
    - Where for compelling and scientifically sound methodological reasons the use of placebo is necessary to determine the efficacy or safety of an intervention and the patients who

receive placebo or no treatment will not be subject to any risk of serious or irreversible harm. Extreme care must be taken to avoid abuse of this option.

33. At the conclusion of the study, patients entered into the study are entitled to be informed about the outcome of the study and to share any benefits that result from it, for example, access to interventions identified as beneficial in the study or to other appropriate care or benefits.
34. The physician must fully inform the patient which aspects of the care are related to the research. The refusal of a patient to participate in a study or the patient’s decision to withdraw from the study must never interfere with the patient-physician relationship.
35. In the treatment of a patient, where proven interventions do not exist or have been ineffective, the physician, after seeking expert advice, with informed consent from the patient or a legally authorized representative, may use an unproven intervention if in the physician's judgement it offers hope of saving life, re-establishing health or alleviating suffering. Where possible, this intervention should be made the object of research, designed to evaluate its safety and efficacy. In all cases, new information should be recorded and, where appropriate, made publicly available.
