# Supplementary material for: Safety after extended repeated use of ulipristal acetate for uterine fibroids
Source: PLoS One. 2017 Mar 7;12(3):e0173523. doi: 10.1371/journal.pone.0173523 (PMC5340384; doi:10.1371/journal.pone.0173523)
Supplement: S2 Text — (PDF) [file pone.0173523.s002.pdf]

PGL11-024

Study Title: A Phase III, multicentre, extension study investigating the efficacy and safety of repeated intermittent 3-month courses of open-label administration of ulipristal acetate, in subjects with symptomatic uterine myomas and heavy uterine bleeding.

PGL11-024 (PEARL extension 2): Extension of study PGL09-027 (itself the extension of study PGL09-026)

Name of Investigational Medicinal Product: PGL4001 (ulipristal acetate, INN: ulipristal)

Short Study Name: PGL4001 Efficacy Assessment in Reduction of symptoms due to uterine Leiomyomata

Indication studied: Repeated intermittent treatment of symptomatic uterine myomas in subjects with heavy uterine bleeding.

**Protocol Identification Number:**	PGL11-024
**Development Phase of the Study:**	Phase III
**Study Initiation Date:**	13-JUL-2010
**Study Completion Date:**	12-MAR-2015
**EudraCT Number:**	2012-001465-33
**Study Sponsor:**	PregLem SA<br>3 Chemin du Pré-Fleuri<br>1228 Plan-les-Ouates, Geneva, Switzerland<br>Phone: +41 22 884 03 40<br>Fax: + 41 22 884 03 49
**Clinical Trial Manager:**	Annie Dacquin
**Global Project Director**	Frédéric Monnot
**Responsible Medical Officer:**	Elke Bestel, MD
**Coordinating Investigator:**	Jacques Donnez, MD, PhD<br>Société de Recherche pour l'Infertilité<br>143, Avenue Grand Champs<br>1150 Brussels, Belgium.
**Date of the report:**	07-JUL-2015

The aim of this extension study PGL11-024 was to investigate the efficacy and safety of repeated intermittent treatment courses of PGL4001 over 4 additional 3-month courses assessing treatment satisfaction of subjects, quality of life and pain, myoma size and uterine bleeding. This study was an optional extension study open to subjects who had completed the 4 treatment courses in PGL09-026 (PEARL III) and its extension study PGL09-027 (PEARL III extension).

The following reports concerning the studies for which PGL11-024 was the extension have been issued:

PGL09-026

A Phase III, multicentre, clinical study investigating the efficacy and safety of 3 months open-label treatment with PGL4001, followed by a randomized, double-blind placebo controlled period of 10 days treatment with progestin, in subjects with myomas and heavy uterine bleeding: Interim Safety Report PGL09-026, dated 12 May 2011.

A Phase III, multicentre, clinical study investigating the efficacy and safety of 3-months open-label treatment with PGL4001, followed by a randomised, double-blind placebo controlled period of 10 days treatment with progestin, in subjects with myomas and heavy uterine bleeding: Updated Interim Safety Report PGL09-026, dated 4 August 2011.

A Phase III, multicentre, clinical study investigating the efficacy and safety of 3-months open-label treatment with PGL4001, followed by a randomised, double-blind placebo controlled period of 10 days treatment with progestin, in subjects with myomas and heavy uterine bleeding: Clinical Study Report PGL09-026, dated 26 June 2012.

PGL09-027

A Phase III, multicentre, clinical study investigating the efficacy and safety of three successive periods of 3-month open-label PGL4001 treatment, each followed by ten days of double-blind treatment with progestin or placebo and a drug-free period until return of menses, in subjects with myomas and heavy uterine bleeding. Interim Safety Report PGL09-027, dated 12 December 2012.

A Phase III, multicentre, clinical study investigating the efficacy and safety of three successive periods of 3-month open-label PGL4001 treatment, each followed by ten days of double-blind treatment with progestin or placebo and a drug-free period until return of menses, in subjects with myomas and heavy uterine bleeding. Clinical Study Report PGL09-027, dated 21 May 2013.

An interim safety report for PGL11-024 has been issued:

PGL11-024

A Phase III, multicentre, extension study investigating the efficacy and safety of repeated intermittent 3-month courses of open-label administration of ulipristal acetate, in subjects with symptomatic uterine myomas and heavy uterine bleeding. Interim Safety Report PGL11-024, dated 25 November 2014.

1 TITLE PAGE

A Phase III, multicentre, extension study investigating the efficacy and safety of repeated intermittent 3-month courses of open-label administration of ulipristal acetate, in subjects with symptomatic uterine myomas and heavy uterine bleeding.

Study No: PGL11-024

Final Report: 07 July 2015

Sponsor:

PregLem SA, 3 Chemin du Pré-Fleuri, 1228, Plan-les-Ouates, Geneva, Switzerland

08/07/15

Signature
Frédéric Monnot
Global Project Director

Date of signature

8/7/2015

Signature
Elke Bestel, MD
Responsible Medical Officer

Date of signature

08.07.2015

Signature
Annie Dacquin,
Clinical Trial Manager

Date of signature

Contract Research Organisation:

Lead Biostatistician
CROS NT Limited, 24 The Broadway, Maidenhead, Berkshire, SL6 1NW, United Kingdom

P.G. Terrill

9 JUL 2015

Signature
Paul Terrill
Project Statistician

Date of signature

This study was designed, conducted, recorded, and reported in compliance with the principles of Good Clinical Practice (GCP) guidelines. The Investigators and Sponsor will store the trial documents for the time period defined in the GCP guidelines and international legislation. These guidelines are stated in United States Federal Regulations as well as "Guidance for GCP," International Conference on Harmonisation (ICH) of Technical Requirements for Registration of Pharmaceuticals for Human Use.

2 SYNOPSIS

NAME OF COMPANY: PregLem SA	Individual Study Table Referring to Part of the Dossier:<br><br>Volume:<br><br>Page:	(FOR NATIONAL AUTHORITY USE ONLY)
NAME OF FINISHED PRODUCT: PGL4001		
NAME OF ACTIVE INGREDIENT: ulipristal acetate		

TITLE OF STUDY: A Phase III, multicentre, extension study investigating the efficacy and safety of repeated intermittent 3-month courses of open-label administration of ulipristal acetate, in subjects with symptomatic uterine myomas and heavy uterine bleeding.

COORDINATING INVESTIGATOR: Jacques Donnez, MD, PhD

STUDY CENTRES: 16 sites (all having participated in PGL09-026 and PGL09-027)

PUBLICATION (REFERENCE): NA

**STUDIED PERIOD:** 13-Jul-2010 to 12-Mar-2015	**PHASE OF DEVELOPMENT:** Phase III

OBJECTIVES:

Primary objective:

- To assess the treatment satisfaction of subjects receiving repeated intermittent ulipristal acetate treatment courses using a Global Study Treatment Satisfaction Questionnaire (GSTSQ).

Secondary objectives:

Efficacy:
- To assess the treatment satisfaction of subjects receiving repeated intermittent ulipristal acetate treatment courses using a GSTSQ (at additional time points).
- To assess quality of life (measured with EuroQol Group Health Outcomes Questionnaire of 5 Dimensions [EQ-5D] and Uterine Fibroid Symptom and Health-Related Quality of Life Questionnaire [UFS-QoL]) and pain (using visual analogue scale [VAS]).
- To assess the sustained efficacy of repeated intermittent 3-month treatment courses of daily administration of ulipristal acetate on myoma size.
- To assess uterine bleeding characteristics (using the Pictorial Bleeding Assessment Chart [PBAC]) at restart of treatment at the beginning of the study in subjects who have already completed 4 previous courses of ulipristal acetate treatment.

Safety:
- To assess the long-term safety of repeated intermittent 3-month administration of ulipristal acetate, specifically with regards to the endometrium and the occurrence of adverse events.

Exploratory objectives:

- To assess the incidence of Progesterone receptor modulator Associated Endometrial Changes (PAEC), 10-18 days after the first menstruation following the last treatment course with ulipristal acetate (visit III).
- To assess the incidence and type of surgery on uterine myomas during the study period.

METHODOLOGY:

Study population: Women of reproductive age, with moderate to severe symptomatic uterus myoma(s). All subjects had completed a total of 4 treatment courses in PGL09-026 (PEARL III) and its extension study PGL09-027 (PEARL III extension).

Study design: This was the optional, long-term, open-label extension of the Phase III study, PGL09-027 (PEARL III extension). During PGL09-026 (PEARL III) and subsequent extension study PGL09-027 (PEARL III extension), subjects had been exposed to a total of 4 treatment courses of 3-months open-label treatment with PGL4001 10 mg once daily, before entering this study, PGL11-024 (PEARL extension 2).

PGL11-024 consisted of 4 further consecutive courses of 3 months (84 days) open-label PGL4001 10 mg once daily treatment, each separated by a drug-free period.

NUMBER OF SUBJECTS (PLANNED AND ANALYSED):

Of the 99 subjects who completed PGL09-026 and PGL09-027, 64 subjects opted to continue in to this second extension study (PGL11-024).

A total of 64 (100.0%), 62 (96.9%), 56 (87.5%) and 54 (84.4%) subjects started PGL4001 treatment courses 5, 6, 7 and 8, respectively. The end of study follow-up visit IV was attended by 53 (82.8%) subjects.

A total of 11 (17.2%) subjects discontinued the study at any time. The reasons for discontinuation were: lack of efficacy (N=2), subject request (N=2), surgery or procedure for fibroids (N=2), pregnancy (N=2), adverse event (not PGL4001-related) (N=1), missing return to menstruation (N=1) and ‘other reason’ (N=1).

MAIN ELIGIBILITY CRITERIA:

To be eligible for inclusion into this study, the subjects fulfilled all of the following criteria:

- provision of written informed consent prior to any study related procedures.
- subject completed visit F – i.e. Follow-up visit of PGL09-027 (PEARL III extension study) - without significant deviations.
- subject had no contra-indication to enter the long term extension study, based on the Investigator’s judgment.
- females of childbearing potential were advised to practice a non-hormonal method of contraception among one of the following:
    - sexual abstinence
    - diaphragms
    - condom or having a partner with a vasectomy with either confirmed azoospermia or performed at least 6 months prior to the study

Subjects who fulfilled any of the following criteria were excluded from participation:

- subject had a history of uterus surgery (e.g. hysterectomy, myomectomy) or uterine artery embolisation in PGL09-027 (PEARL III extension) or afterwards that would interfere with the study assessments.
- subject had taken or was likely to require treatment during the study with drugs that were not permitted by the study protocol: progestins (systemic or progestin releasing intra-uterine system), oestrogens, gonadotropin-releasing hormone (GnRH)-agonists/antagonists, hormonal contraceptives, systemic glucocorticoids (oral and injectable), and/or treatments with potent

inhibitors or inducers of cytochrome P450 3A4 (CYP3A4). Wash-out periods prior to Visit I were as follows:
  - progestins and oestrogens: 1 month
  - GnRH-agonists/antagonists: 3 months
  - systemic glucocorticoids: 1 month (3 months after depot injections)
  - CYP3A4 inhibitors or inducers: 2 weeks
- subject was lactating, had a positive pregnancy test at study start or was planning a pregnancy during the course of the study.
- subject had a current problem with alcohol or drug abuse.
- subject had a mental condition rendering her unable to understand the nature, scope and possible consequences of the study, and/or evidence of an uncooperative attitude.
- subject had abnormal baseline findings, any other medical condition(s) or laboratory finding that, in the opinion of the Investigator, might have jeopardised the subject's safety or interfered with study evaluations.

TEST PRODUCT, DOSE, AND MODE OF ADMINISTRATION; BATCH NUMBER:
All subjects enrolled into the study received open-label treatment with PGL4001 10 mg tablets (batches U19010 and U21016), administered orally once daily.

DURATION OF TREATMENT:
This study included 4 treatment courses. Each treatment course consisted of 3-months (84 days) once daily oral treatment with PGL4001 10 mg. Treatment courses were separated by a drug-free period, until the start of second menses following the end of the previous treatment course.

CRITERIA FOR EVALUATION:

Primary endpoints:
- Myoma symptom control assessed with a GSTSQ at visit II (average score of the first 3 questions).
- Myoma symptom control assessed with a GSTSQ at visit III (average score of the first 3 questions).

Secondary endpoints:
Efficacy:
- Myoma symptom control assessed with a GSTSQ at visit I (average score of the first 3 questions).
- Myoma symptom control assessed with a GSTSQ at visit IV (average score of the first 3 questions).
- Myoma symptom control assessed with a GSTSQ at visits I, II, III and IV (individual component scores).
- Change from visit 2 of PGL09-026 (PEARL III) and visit F of PGL09-027 (PEARL III extension) to visits II, III and IV in quality of life (using EQ-5D and UFS-QoL) and in pain (using VAS).
- Change from visit 1 of PGL09-026 (PEARL III) and visit F of PGL09-027 (PEARL III extension) to visits II, III and IV in myoma size, measured on the three largest myomas identified at visit 1 of PGL09-026, by transvaginal ultrasound.
- Change from the first menstruation at the start of PGL09-026 (PEARL III) study to the first menstrual bleeding at re-start of the first ulipristal acetate treatment course (in PGL11-024) in strength of the bleeding, using the PBAC.

Safety:
- Number and proportion of subjects experiencing treatment-emergent adverse events including subject reported adverse events and clinically significant changes in the parameters listed below:
  - Laboratory parameters (haematology, chemistry, lipids).
  - Change from visit F of PGL09-027 (PEARL III extension) to visits II, III and IV in endometrial thickness assessed by transvaginal ultrasound.
  - Visit III endometrium biopsy (i.e. hyperplasia, adenocarcinoma).

Exploratory endpoints**:**

- Frequency of PAEC observed in the endometrial biopsy, on day 10-18 after the first menstruation following the last treatment course with ulipristal acetate (visit III).
- Proportion of subjects having surgery or any invasive procedure for myoma treatment at any time during the study (type of surgery/procedure performed).

STATISTICAL METHODS:

Data from all subjects that were enrolled in PGL11-024 are presented in individual subject data listings and tables. In general, data from PGL09-026 and PGL09-027 are included in the individual subject data listings, for subjects that have entered this extension study PGL11-024. Summaries by visit also include visits from PGL09-026 and PGL09-027. Treatment courses are numbered as treatment course 1 (PGL09-026), treatment courses 2 to 4 (PGL09-027) and treatment courses 5 to 8 (PGL11-024) wherever data are presented by treatment course. In studies PGL09-026 and PGL09-027 subjects were randomised and exposed to 10 days of treatment with either norethisterone acetate (NETA) or placebo immediately after each 3-month course of PGL4001, whereas in PGL11-024 no NETA/placebo treatment was given. Consequently there is no differentiation between the NETA/placebo treatment groups for the summaries produced in this extension study.

Categorical (qualitative) assessments are summarised in contingency tables provided by visit. Categorical assessments are tabulated using frequencies and percentages. Continuous (quantitative) assessments are summarised via descriptive statistics by visit. Changes (and/or percent changes) respective to baseline are evaluated where applicable. Continuous assessments are tabulated using the following summary statistics: number of values, mean, standard deviation, median, the first quartile (Q1), the third quartile (Q3) and the minimum and maximum values.

Data were summarised for all subjects treated during PGL11-024.

SUMMARY

EFFICACY RESULTS:

A total of 64 subjects opted to continue in to this second extension study, PGL11-024, from the 99 subjects who completed studies PGL09-026 and PGL09-027, and more than 80% of these subjects remained in the study until the 3 month follow-up visit following treatment course 8. The FAS comprised 64 subjects, and the Completers Set comprised 53 subjects; the outcome of efficacy analyses for the Completers Set was similar to those for the FAS.

Myoma symptom control assessed with a Global Study Treatment Satisfaction Questionnaire.

Subjects completed the GSTSQ at every PGL11-024 visit: visit I (approximately 3 months after treatment course 4), visit II (10-18 days after the first menstruation following treatment course 6), visit III (10-18 days after the first menstruation following treatment course 8) and visit IV (3 month follow-up after last dose of treatment course 8). The GSTSQ consists of 4 questions as follows:

1. How satisfied or dissatisfied are you with the ability of the study drug to prevent or treat your fibroid symptoms?
2. How satisfied or dissatisfied are you with the way the study drug relieves the uterine bleeding due to your fibroids symptoms?
3. Taking all things into account, how satisfied or dissatisfied are you with this study drug?
4. How do you estimate your menstrual bleeding now compared to before the very first intake of this study drug?

The mean responses (graded from 1=extremely satisfied to 7=extremely dissatisfied) to the first 3 questions concerning control of fibroid symptoms, control of uterine bleeding and overall satisfaction showed that, on average, subjects were very satisfied with the treatment and this satisfaction was maintained throughout the study and up to 3 months following the end of treatment. At visit I the mean (interquartile range) response was 2.06 (1.33, 2.50), at visit II it was 2.04 (1.33, 2.67), at visit III it was 1.93 (1.00, 2.67) and at visit IV (3 month follow-up visit) it was 1.95 (1.00, 2.67) (FAS).

Individual responses to all 4 questions for the GSTSQ were also summarised (FAS):
Question 1: How satisfied or dissatisfied are you with the ability of the study drug to prevent or treat your fibroid symptoms?
For the control of fibroid symptoms question, the percentage of subjects who reported being either very satisfied or extremely satisfied were 76.6% at visit I, 68.3% at visit II, 67.3% at visit III and 70.6% at visit IV. Only one subject at each of visits II and IV reported being dissatisfied and no subjects reported being very dissatisfied or extremely dissatisfied.
Question 2: How satisfied or dissatisfied are you with the way the study drug relieves the uterine bleeding due to your fibroids symptoms?
For the control of uterine bleeding question, the percentage of subjects who reported being either very satisfied or extremely satisfied were 76.6% at visit I, 78.3% at visit II, 83.6% at visit III and 76.5% at visit IV. Only one subject at visit I reported being dissatisfied and no subjects reported being very dissatisfied or extremely dissatisfied.
Question 3: Taking all things into account, how satisfied or dissatisfied are you with this study drug?
For the overall satisfaction question, the percentage of subjects who reported being either very satisfied or extremely satisfied were 70.3% at visit I, 63.3% at visit II, 71.4% at visit III and 72.6% at visit IV. No subjects reported being dissatisfied, very dissatisfied or extremely dissatisfied.
Question 4: How do you estimate your menstrual bleeding now compared to before the very first intake of this study drug?
For the question evaluating menstrual bleeding compared to before first intake of study drug (before starting study PGL09-026, PEARL III), responses ranged from 1=much less heavy to 5=much heavier. The percentage of subjects who reported either much less heavy or a little less heavy were 79.7% at visit I, 93.3% at visit II, 95.9% at visit III and 92.2% at visit IV. Two subjects reported bleeding being a little heavier at visit IV, and one subject reported bleeding being much heavier at visit I.
In summary, the majority of subjects (>75%) were either very or extremely satisfied with control of uterine bleeding throughout the study, and menstrual bleeding compared to before the first intake of PGL4001 was less heavy (either much less or a little less heavy) in almost 80% of subjects at the start of PGL11-024 and increased to almost 96% of subjects at visit III (10-18 days after first menstruation following treatment course 8). Between 60-80% of all subjects reported being either very or extremely satisfied with the control of fibroid symptoms and overall satisfaction questions at all visits including visit IV (3 month follow-up), demonstrating sustained improvement of symptoms.

<u>Quality of Life</u>
An important improvement in quality of life was recorded during treatment, reaching scores reported for healthy individuals. Quality of life was measured using the general EQ-5D questionnaire, and also by the specific UFS-QoL, which measured symptom severity and HRQL.
For the EQ-5D, the most problems reported at baseline and throughout the study were in the pain/discomfort and anxiety/depression dimensions. At baseline, moderate pain/discomfort was reported by 78.1% of subjects; this decreased following treatment and at the follow-up visit of PGL09-027 moderate pain/discomfort was reported by only 28.1% of subjects (FAS). During PGL11-024, the percentage of subjects reporting moderate pain/discomfort was 27.1% at visit II (following the second treatment course in PGL11-024, sixth treatment course overall), 24.5% at visit III (following the fourth treatment course in PGL11-024, eighth treatment course overall) and 25.0% at visit IV (3 month follow-up). One subject reported extreme pain/discomfort at visit II.
Moderate anxiety/depression was reported by 60.9% of subjects at baseline, and extreme anxiety/depression by 3 (4.7%) subjects. This showed improvement at all subsequent visits during PGL09-026 and PGL09-027. During PGL11-024, 29.3%, 32.7% and 36.5% of subjects reported moderate anxiety/depression at visits II, III and IV respectively, with no subjects reporting extreme anxiety/depression.
In the UFS-QoL assessment, the mean (median) symptom severity score at baseline was 47.27 (50.00) (FAS). The average symptom severity score showed an improvement (decrease) at the end of treatment course 1 in study PGL09-026, with a mean (median) change from baseline of -35.86 (-40.63). This improvement was maintained following treatment courses 2, 3 and 4 in study PGL09-027. The improvement from baseline was less on average at the follow-up visit in study PGL09-027 (visit F), at -23.39 (-21.88). At visits II and III of PGL11-024, mean (median) change from baseline was -29.18 (-31.25) and -31.65 (-34.38), respectively, similar to seen following earlier treatment courses during PGL09-026 and PGL09-027. At visit IV (3 month follow-up) the mean (median) change from baseline was -25.7 (-25.0).

The average symptom severity score in healthy women is 22.5. The mean (median) symptom severity scores from the end of treatment course 1 in PGL09-026 through to the follow-up visit in PGL11-024 indicated that, on average, symptom severity was restored to the level of healthy women after the first course of PGL4001 treatment and was sustained until 3 months after the end of the eighth treatment course.

The pattern of improvement was similar for the HRQL scales (improvement demonstrated by increase). The mean (median) HRQL total score at baseline was 57.29 (54.67) for the FAS. In a population of healthy women the average HRQL score is 86.4. The mean (median) HRQL total score at the end of treatment course 1 in study PGL09-026 was 90.35 (94.83), with a change from baseline of 33.06 (32.33). As with symptom severity, the improvement was maintained following treatment courses 2, 3 and 4. The improvement from baseline was slightly less on average at follow-up visit in study PGL09-027 (visit F), with a mean (median) change from baseline of 24.49 (23.71). In PGL11-024, the subsequent mean (median) change from baseline at visits II and III were 28.61 (30.60) and 27.75 (29.31), respectively. At visit IV (3 month follow-up) the mean (median) change from baseline was 23.15 (23.28) and mean (median) total HRQL score was 82.27 (86.64).

Pain assessment

Overall, pain, assessed by VAS, was substantially reduced from baseline during the study and this reduction was sustained until the PGL11-024 follow-up visit 3 months after end of treatment.

The mean (median) pain VAS score at baseline was 34.7 (30.5) for the FAS. The average pain VAS score showed an improvement (decrease) at the end of treatment course 1 (study PGL09-026) with a mean (median) change from baseline of -26.1 (-17.0). This improvement was maintained following treatment courses 2, 3 and 4 from study PGL09-027.

In study PGL11-024, at visit II (10-18 days after the first menstruation following treatment course 6) and visit III (10-18 days after the first menstruation following treatment course 8); the mean (median) change from baseline in pain VAS score was -23.9 (-20.0) and -30.2 (-24.0), respectively.

At visit IV (3 month follow-up) the mean (median) change from baseline was -25.3 (-23.0), demonstrating the sustained improvement at 3 months after the end of treatment.

The number of subjects showing an improvement from baseline of ≥30% and ≥50% supported the pattern of improvement in pain which was sustained to 3 months after the end of treatment. At the end of treatment course 1, 51 (79.7%) subjects reported having an improvement from baseline of ≥30% and 47 (73.4%) subjects reported having an improvement from baseline of ≥50%. This improvement was maintained at visits B, C and D (10-18 days after the first menstruation following treatment courses 2, 3 and 4 in study PGL09-027).

In study PGL11-024, 44 (74.6%) and 40 (81.6%) subjects reported having an improvement from baseline of ≥30% at visits II and III (10-18 days after the first menstruation following treatment courses 6 and 8) respectively, and 42 (71.2%) and 35 (71.4%) subjects reported having an improvement from baseline of ≥50%.

At visit IV (3 month follow-up visit), 39 (75.0%) and 38 (73.1%) subjects reported having an improvement from baseline of ≥30% and ≥50%, respectively.

Myoma volume reduction

The total volume of the 3 largest myomas identified at screening was shown to decrease following the first treatment course, with the decrease maintained in most subjects up to visit F/I. At visit F/I the reduction from baseline was reduced, which may reflect the gap of 3 months or longer between the end of treatment course 4 and the start of PGL11-024 (the mean [median] gap between follow-up visit F of PGL09-027 and visit I of PGL11-024 was 103.9 [91.0] days). During PGL11-024 the reduction in total myoma volume was maintained for most of the subjects up to the 3 month follow-up visit.

For the FAS, the mean (median) total volume of the three largest myomas at screening was 68.13 cm^3 (41.39 cm^3). At visit 5 (end of treatment course 1 in study PGL09-026), the average total volume of the three largest myomas had reduced from screening with the mean (median) total volume being 34.64 cm^3 (13.55 cm^3). The mean (median) percent change from screening was -53.83% (-59.19%). The median reduction was maintained at visit 6 (10-18 days after the first menstruation following treatment course 1 from study PGL09-026) and further decreased at visits B, C and D (10-18 days after the first menstruation following treatment courses 2, 3 and 4 from study PGL09-027). The largest reduction was seen at visit D, with a mean (median) percent change from screening of -56.55% (-77.30%).

The mean (median) reduction from screening at visit F (approximately 3 months after treatment end, follow-

up visit in study PGL09-027) was -33.63% (-63.91%). Of the 34 subjects who had an assessment at visit I (start of study PGL11-024), the mean (median) percent change from screening was 6.7% (-48.06%). These subjects had undergone a reassessment of myoma volume at visit I in addition to visit F, as they had a gap of >3 months between visit F and the start of PGL11-024.
Overall, for all subjects attending visits II, III (10-18 days after the first menstruation following treatment courses 6 and 8, respectively) and follow-up visit IV, the mean (median) percent change from screening was -1.06% (-68.87%), -28.46% (-67.10%) and -15.16% (-70.34%), respectively. The distribution between mean and median results were due to skewed distributions, with a few outlying subjects having a larger influence on the mean values than the medians.
In terms of the percentage of subjects with ≥25% and ≥50% reduction in the total volume of the 3 largest myomas identified at screening, at visit 5, 53 (84.1%) subjects had a volume reduction of ≥25% and 37 (58.7%) subjects had a volume reduction of ≥50%. The percentage of subjects with a volume reduction from screening of ≥25% was maintained at visits 6 and B, C and D whilst the percentage of subjects with a volume reduction from screening of ≥50% increased. At visit D, the number (percent) of subjects with a volume reduction from screening of ≥25% was 52 (86.7%) and with a volume reduction of ≥50% was 48 (80.0%). At visit F, 46 (73.0%) subjects had a volume reduction of ≥25% and 39 (61.9%) subjects had a volume reduction from screening of ≥50%. The percentages did not change much for the remainder of the study. At visit IV (3 month follow-up), 39 (76.5%) subjects had a volume reduction from screening of ≥25% and 34 (66.7%) subjects had a volume reduction of ≥50%.

Comparison of uterine bleeding characteristics (using the PBAC) at re-start of treatment in PGL11-024 compared with the first menstruation at the start of PGL09-026.
The first menses post-screening in study PGL09-026, days 1 to 8, had a mean (median) PBAC score of 284.1 (205.5). There was a reduction, on average, for the 8 day PBAC score for the first menstruation post-treatment courses 1, 2, 3 and 4, with a mean (median) PBAC score of 131.6 (89.0), 85.5 (48.5), 79.9 (36.0) and 65.4 (31.0), respectively. The mean (median) PBAC score of the first menstruation at re-start of treatment in PGL11-024 had increased compared to those post-treatment courses 1, 2, 3 and 4, with a mean (median) of 148.0 (84.0). However, this was still lower than the PBAC score for the first menses post-screening, with a mean (median) change of -129.9 (-88.5).

Surgery
At the start of the PGL09-026 study there was no surgery planned for 30 (46.9%) subjects (FAS). During PGL11-024, surgery was performed on 4 out of 64 (6.3%) subjects (FAS); for 3 of these, surgery was not planned at the start of PGL09-026. Of the 4 subjects that had surgery performed, 2 had a laparotomic hysterectomy, 1 had an abdominal hysterectomy and 1 had surgery but the procedure was ‘not known’ (the subject had planned to undergo laparoscopic myomectomy). The decision drivers for the 4 subjects that had surgery performed were reported as ‘mainly driven by subject request’ for 3 subjects and ‘equal influence’ for 1 subject (whose surgery was unplanned). Three subjects reported the main reason for surgery as ‘insufficient efficacy of treatment’, and for one subject no reason was provided except the comment ‘abdominal hysterectomy’.

Uterine volume reduction
The uterine volume as measured by US was shown to decrease following the first treatment course in study PGL09-026. The mean (median) uterine volume at screening was 222.35 cm^3 (168.30 cm^3) (FAS). At the end of treatment course 1, the average uterine volume had reduced from screening with a mean (median) percent change from screening of -30.81% (-36.92%). This reduction in uterine volume was maintained following treatment courses 2, 3 and 4 in study PGL09-027.
The reduction from screening was less on average at visit F (approximately 3 months after treatment end, follow-up visit in study PGL09-027) but was still present, with a mean (median) percent change from baseline of -20.31% (-30.14%). Of the 34 subjects who had an assessment at visit I (start of study PGL11-024) the mean (median) percent change from baseline in uterine volume was -4.68% (-16.15%). These subjects had undergone a reassessment of uterine volume at visit I in addition to visit F, as they had a gap of >3 months between visit F and the start of PGL11-024, which may explain the reduction in change from screening compared to that seen at previous and subsequent visits.
Overall, for subjects attending visits II, III and follow-up visit IV, the mean (median) percent change from baseline was -15.87% (-34.21%), -6.88% (-26.47%) and -2.40% (-20.08%), respectively. The mean (median)

percent change from visit F/I was -4.36% (-3.65%), -1.55% (-2.12%) and 0.77% (-1.91%) at visits II, III and follow-up visit IV, respectively. A few subjects saw an increasing uterine volume at visits I, II, III and IV compared to previous visits, in particular subjects 30/16, 32/06 and 38/05.
The percentage of subjects with ≥25% and ≥50% reduction from visit 1 (screening/baseline) (and from visit F/I) in uterine volume was also assessed. At visit 5, 38 (59.4%) subjects had a volume reduction from baseline of ≥25% and 14 (21.9%) subjects had a volume reduction from baseline of ≥50%. This reduction was maintained at visit 6 (post treatment course 1 from study PGL09-026) and at visits B, C and D (post treatment courses 2, 3 and 4 in study PGL09-027). The largest reduction in uterine volume was seen at visit D, when 44 (73.3%) subjects had a volume reduction from baseline of ≥25% and 29 (48.3%) subjects had a volume reduction from baseline of ≥50%. At visit F, 35 (54.7%) subjects had a volume reduction from baseline of ≥25% and 16 (25.0%) subjects had a volume reduction from baseline of ≥50%.
At visit II (10-18 days after first menstruation following treatment course 6), 34 (55.7%) subjects had a volume reduction from baseline of ≥25% and 15 (24.6%) subjects had a volume reduction from baseline of ≥50%, and a similar reduction was seen at visit III (10-18 days after first menstruation following treatment course 8).
At visit IV (follow-up visit), 24 (46.2%) subjects had a volume reduction from baseline of ≥25% and 12 (23.1%) subjects had a volume reduction from baseline of ≥50%.

SAFETY RESULTS:
A total of 64 (100.0%), 62 (96.9%), 56 (87.5%) and 54 (84.4%) subjects started PGL4001 treatment courses 5, 6, 7 and 8, respectively, and 53 (82.8%) subjects completed the study and attended the end of study follow-up visit. Study PGL09-026 began in July 2010 and study PGL11-024 was completed in March 2015, hence the majority of subjects who completed PGL11-024 were study participants for approximately 4 years. Safety data for PGL4001 treatment course 1 has been reported in the PGL09-026 CSR, for treatment courses 2-4 in the PGL09-027 CSR, and these data have not been discussed again unless of relevance to interpretation of changes seen during PGL11-024. In both of these studies PGL4001 treatment was followed by 10 days of treatment with either placebo or NETA; this was not continued in PGL11-024 as a 10-day course of NETA treatment was not shown to add significant benefit.
Safety has been determined by evaluation of a number of endpoints, summarised below:

<u>Treatment Emergent Adverse Events</u>
There have been no deaths during studies PGL09-026, PGL09-027 or PGL11-024. Treatment emergent adverse events were reported as either on- or off-treatment, depending on timing in relation to each PGL4001 treatment course.
Overall, during treatment courses 1-8 of the study, 47 (73.4%) subjects reported 189 on-treatment TEAEs. The incidence of on-treatment TEAEs reported was highest during treatment course 1, 68 events reported by 32 (50%) subjects, decreasing to 15 (23.4%) subjects reporting 17 and 27 TEAEs during treatment courses 2 and 3, respectively. Thirty-one TEAEs were reported by 22 (34.4%) subjects during treatment course 4. In PGL11-024, during treatment course 5, a total of 14 TEAEs were reported by 10 (15.6%) of the 64 subjects who started treatment course 5; during treatment course 6, a total of 15 TEAEs were reported by 12 (19.4%) of the 62 subjects; during treatment course 7, eleven TEAEs were reported by 8 (14.3%) of the 56 subjects, and during treatment course 8, six TEAEs were reported by 5 (9.3%) of the 54 subjects.
Overall, following treatment courses 1-8 of the study, 43 (67.2%) subjects reported 107 off-treatment TEAEs. No trends were observed in frequency or type of TEAEs with repetition of treatment courses.
No SAEs were reported during PGL11-024.
One subject was diagnosed as pregnant during treatment course 5, with an outcome of ectopic kidney in the child. The outcome for the child was reported as an SAE recorded in the Sponsor's safety database, but as the child was not a subject within the study this SAE is not captured in tables produced for PGL11-024.
One subject withdrew from PGL11-024 approximately 2 months following the end of treatment course 7, as she chose to undergo an abdominal hysterectomy.
One AESI of "endometrial thickening" (PT: endometrial hypertrophy) of mild intensity was reported during treatment course 7; the TEAE was considered related to PGL4001 and was resolved 6 weeks later.
Overall, during treatment courses 1-8, the majority (98.4%) of all on-treatment TEAEs were rated as being of mild or moderate intensity; of the 3 TEAEs of severe intensity only one occurred during PGL11-024, a report of bronchitis during treatment course 6, which was considered unrelated to PGL4001. Following treatment courses 1-8, the majority (95.3%) of all off-treatment TEAEs were rated as being of mild or moderate

intensity; of the 5 TEAEs of severe intensity only one occurred during PGL11-024, a report of sciatica following treatment course 8, which was considered unrelated to PGL4001.
During treatment courses 1-8, the most commonly reported on-treatment TEAE was headache, with the greatest incidence during treatment course 1: headache was reported by 7 (10.9%) subjects during treatment course 1, none during treatment course 2, by 3 (4.7%) subjects during treatment course 3, by 2 (3.1%) subjects during treatment courses 4 and 5, then by 3 (4.8%), 2 (3.6%) and 2 (3.7%) subjects during treatment courses 6, 7 and 8, respectively. Overall, following treatment courses 1-8, the most commonly reported off-treatment TEAE was dysmenorrhoea, in 5 (7.8%) subjects.
A total of 20 (31.3%) subjects experienced at least one on-treatment TEAE considered to be PGL4001-related. These were most frequently reported during treatment course 1, when 13 (20.3%) subjects reported 24 on-treatment, PGL4001-related TEAEs including hot flush and headache (3 [4.7%] subjects each). During treatment courses 2-8 only single incidences of PGL4001-related on-treatment TEAEs were reported with the exception of hot flush (3 [4.7% subjects), and headache (2 [3.1%] subjects) during treatment course 3, and hot flush (2 [3.6%] subjects) during treatment course 7.
Following treatment courses 1-8, a total of 8 (12.5%) subjects experienced 9 off-treatment TEAEs considered to be PGL4001-related, including hot flush (2 subjects).
Overall, there was no evidence that any specific TEAE increased in frequency or intensity as the number of PGL4001 treatment courses increased, nor were any unexpected TEAEs reported during PGL11-024.

Laboratory Parameters
Evaluation of laboratory parameters, including changes from screening (assessed during PGL09-026) was undertaken as part of the review of safety. No changes in the number and type of laboratory results falling outside of the normal ranges were observed as the number of PGL4001 treatment courses increased.
As seen in previous studies investigating PGL4001 in subjects with uterine myoma associated with heavy uterine bleeding, Hb levels and associated haematology parameters increased during PGL09-027 compared with screening (PGL09-026), and the increase was maintained at visit III (10-18 days after the start of menses following treatment course 8) of PGL11-024.
Mean levels of AST and ALT did not alter during PGL11-024, compared with levels at screening. Only one subject had AST levels >ULN (<2 x ULN) during PGL11-024, 3 subjects had raised GGT values (all <2 x ULN), but no TEAEs concerning biochemistry parameters were reported.
At screening, 55.6% of subjects had a total cholesterol level >ULN. For this reason, many subjects continued with high cholesterol levels throughout the study, and a similar percentage was seen at visit III (53.1%); no TEAEs of hypercholesterolaemia or hypertriglyceridaemia were reported during PGL11-024. The ratio of total cholesterol/HDL cholesterol did not change, and remained below the level at which the risk of cardiovascular disease is considered to be increased (<3.5).

Vital Signs
There were no changes in vital signs, including blood pressure, during PGL11-024.

Endometrium thickness
The median endometrium thickness during PGL11-024 remained below that seen at screening, with no evidence of an increase in the number of subjects with a thickness >16 mm as the number of PGL4001 treatment courses increased. At screening (PGL09-026), for the 62 subjects with data available, the median endometrium thickness was 9.0 mm (range 3-21 mm), and 2 (3.2%) subjects had thickness >16 mm. At visit III (10-18 days after the start of menses following treatment course 8), for the 49 subjects with data available, the median endometrium thickness was 7.0 mm (range 3-23 mm), and 1 (2.0%) subject had thickness >16 mm. At visit IV (follow-up visit approximately 3 months after last dose of PGL4001), for the 53 subjects with data available, the median endometrium thickness remained at 7.0 mm (range 1-16 mm), and no subjects had thickness >16 mm.

Endometrium histology
Endometrium biopsy samples were evaluated by the same 3 independent pathologists in study PGL11-024, as performed for studies PGL09-026 and PGL09-027. A consensus diagnosis of benign endometrium was made for all (100%) endometrium biopsy samples adequate for histology review, taken from screening of study PGL09-026 (96.2% adequate biopsies) through to visit IV of study PGL11-024 (91.7% adequate biopsies). All 3 individual pathologists provided a diagnosis of benign endometrium in all evaluations with the

exception of one observation of complex, non-atypical, hyperplasia seen in one biopsy taken following treatment course 4 in PGL09-027, and not seen subsequently in individual biopsy assessments.

No additional observations were reported for biopsy samples during study PGL11-024 except the presence of polyps, seen for 2 subjects at visit I and 2 subjects at visit III. At visit I, hyperplasic polyp was diagnosed for one subject and benign polyp for one subject; for both of these a visit II biopsy was not done, but the polyps were absent at the visit III biopsy. At visit III, benign polyp was diagnosed for 2 subjects (absent at visit IV biopsy).

Summaries of non-physiological changes observed by at least 2 of the 3 pathologists suggested that after screening, when non-physiological changes were observed in 18.0% of biopsies, the incidence in the full study population was highest in biopsies taken following treatment course 1, seen in 35.0% of biopsies, and then subsequently decreased. Following treatment course 4 (visit E in PGL09-027), at least 2 pathologists observed non-physiological changes in 21.4% of biopsies. At visit III in PGL11-024 (10-18 days after the first menstruation following treatment course 8) non-physiological changes were observed in 16.3% of biopsies, which is comparable to baseline frequency.

At visit IV (follow-up visit) biopsies were to be performed only if 1 or more pathologists had provided a diagnosis other than "benign physiologic endometrium" for the biopsy at visit III or if the biopsy was judged to be not adequate for histology review. A follow-up additional biopsy was provided by 24 subjects at visit IV, and 22 biopsies were considered adequate for review. In this subgroup, non-physiological changes were observed by at least 2 pathologists in 9.1% of biopsies. However, taking into consideration that for more than half of all subjects no follow-up biopsy had been requested due to a diagnosis of "benign endometrium" from all 3 pathologists at visit III, the observation of non-physiological changes in the full study population decreased to <5%, which is below baseline frequency and confirms the previously described rapid reversibility of non-physiological changes once treatment is stopped and menstruation returns.

CONCLUSION:

In summary, this extension study PGL11-024 has demonstrated that the intermittent administration of an additional 4 treatment courses of PGL4001 10 mg once daily for 3 months with drug-free intervals, bringing the total number of PGL4001 treatment courses undertaken to 8, is well tolerated in women of reproductive age with symptomatic uterine myoma (studies PGL09-026, PGL09-027 and PGL11-024). Repetition of PGL4001 treatment courses did not lead to any changes of concern in endometrial histology, nor to increases in endometrial thickness. The frequency of non-physiological changes observed did not increase with repeated treatment courses and, as previously demonstrated, non-physiological changes were rapidly reversible. Control of fibroid symptoms, uterine bleeding and overall satisfaction assessed using the GSTSQ showed that, on average, subjects were very satisfied with the treatment and this satisfaction was maintained throughout the study and up to 3 months following the end of the eighth treatment course. In addition, an important improvement in quality of life was observed following the first treatment course which was sustained thereafter over the 8 treatment courses until study follow-up, reaching scores reported for healthy individuals.

DATE OF THE REPORT: 07 July 2015
